# Particle characterization and quantification of organic and inorganic compounds from Chinese and Iranian aerosol filter samples using scanning laser desorption/ionization mass spectrometry

**DOI:** 10.1007/s00216-022-04275-1

**Published:** 2022-09-01

**Authors:** Christof Barth, Klaus-Peter Hinz, Bernhard Spengler

**Affiliations:** Institute of Inorganic and Analytical Chemistry, Justus Liebig University Giessen, 35392 Giessen, Hessen, Germany

**Keywords:** Aerosol composition, Laser desorption ionization mass spectrometry, Pollution, Inorganic particles, Organic particles, Megacities

## Abstract

Besides their influence on climate and cloud formation, many organic and inorganic substances in aerosol particles pose a risk to human health. Namely, polycyclic aromatic hydrocarbons (PAH) and heavy metals are suspected to be carcinogenic or acutely toxic. The detection and quantification of such compounds is difficult if only small amounts of particulate matter (PM) are available. In addition, filter samples are often complex and time-consuming to prepare for chromatographic measurements and elemental analysis. Here, we present a method based on high-resolution atmospheric pressure laser desorption ionization mass spectrometry imaging (AP-LDI-MSI) and statistical analysis which allows the analysis and characterization of very small sample quantities (< 30 µg) without any sample preparation. The power and simplicity of the method is demonstrated by two filter samples from heavily polluted mega cities. The samples were collected in Tehran (Iran) and Hangzhou (China) in February 2018. In the course of the measurement, more than 3200 sum formulae were assigned, which allowed a statistical evaluation of colocalized substances within the particles on the filter samples. This resulted in a classification of the different particle types on the filters. Finally, both megacities could be distinguished based on characteristic compounds. In the samples from Tehran, the number of sulphur-containing organic compounds was up to 6 times as high as the samples from Hangzhou, possibly due to the increasing efforts of the Chinese government to reduce sulphur emissions in recent years. Additionally, quantification of 13 PAH species was carried out via standard addition. Especially, the samples from Tehran showed elevated concentrations of PAHs, which in the case of higher-molecular-weight species (> *m*/*z* 228) were mostly more than twice as high as in Hangzhou. Both cities showed high levels of heavy metals and potentially harmful organic compounds, although their share of total particulate matter was significantly higher in the samples from Tehran. The pre-treatment of the samples was reduced to a minimum with this method, and only small amounts of particles were required to obtain a comprehensive picture for a specific filter sample. The described method provides faster and better control of air pollution in heavily polluted megacities.

**Graphical abstract:**

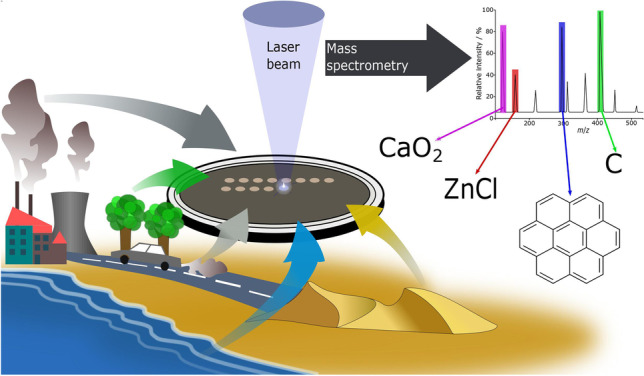

**Supplementary Information:**

The online version contains supplementary material available at 10.1007/s00216-022-04275-1.

## Introduction

Increasing air pollution through the emission of gases and particles is becoming a major problem in many heavily populated regions of the world [1]. Urban aerosols, especially in heavily polluted megacities, are complex mixtures of organic and inorganic substances which, in addition to their effect on the climate, are of particular relevance because of their harmful effects on human health. Their emission leads to an increase in mortality among the population of many megacities around the world through lung cancer, chronic obstructive pulmonary diseases, ischemic heart disease, stroke and respiratory tract infections [2–4]. Calculations show that about 3.3 million premature deaths worldwide can be attributed to outdoor air pollution [5]. Emissions from industry, traffic and private households dominate the air pollution in larger cities [6]. Some megacities, for example on the Arabian Peninsula or in China, are an exception, as the strong seasonal influence of desert sand transport, and thus the proportion of mineral dust, can become dominant here. At times, the proportion of mineral dust can exceed 40% of the total particle mass with industrial pollutants adding to mineral components [7, 8].

Globally, about 82% of the population is exposed to annual mean levels of PM_2.5_ or PM_10_ that exceed World Health Organization (WHO) guidelines [5, 9]. The most polluted cities worldwide, like for example Varanasi (India), Shijiazhuang (China) and Peshawar (Pakistan), are located in East Asia and on the Arabian Peninsula [9]. In many countries such as China, India, Pakistan and some Arab countries, the focus is on increasing productivity at low cost rather than on air pollution control through expensive and complex filter technologies and low-emission processes [10–12]. The most important factors affecting an increase in air pollution and the emission of climate-damaging gases are the coal-dominated energy sector and traffic emissions [13]. Both are a direct consequence of population increase, rising urbanisation and the desire for more technology and prosperity [14]. However, while China and India are trying to reduce emissions of industrial waste, heavy metals in exhaust gases or sulphates in the flue gas of power plants through emission control measures, there is a lack of such regulations in Iran [11].

Commonly used methods for characterizing air quality are based on measuring particle number concentrations with condensation nucleus counters (CNC), optical particle counters (OPC), total particulate mass concentrations by using particle filter samples or impactors or measurements of black carbon concentrations by an aethalometer [15–19]. However, these methods only provide information on how many particles of a certain type or a certain size are present in the air and whether they can penetrate deep into the lungs to cause damage. It is not possible to make statements about the toxicity of the particles with these methods. For more detailed statements on air quality, it is therefore essential to know the chemical composition of atmospheric particles [20]. This allows for a better understanding of the danger of the particles, their origin and possible chemical changes caused by reactions in the atmosphere. Nowadays, the detection and identification of various inorganic and organic compounds is usually carried out using mass spectrometric techniques. Numerous methods based on mass spectrometry (MS), typically coupled with chromatographic separation techniques, are available. These allow a comprehensive chemical characterization of particle samples. Among others, high-performance liquid chromatography–mass spectrometry (HPLC–MS), gas chromatography–mass spectrometry (GC–MS), inductively coupled plasma–mass spectrometry (ICP-MS) and ion chromatography–mass spectrometry (IC-MS) offer the possibility to analyze aerosol compounds with high quantitative accuracy [21–24]. However, these methods go along with comprehensive and time-consuming filter preparation steps and pre-separation of the substances or substance classes prior to mass analysis [25, 26]. On the other hand, it is not possible to attribute the measured chemical properties to individual particles with these methods. Furthermore, it is possible that the particles on the filter surface are modified during sampling, transport, storage and processing of the sample, effects known as sampling artifacts [27]. Extraction and digestion of the filter samples typically takes hours and is associated with a high consumption of chemicals and often low recovery rates, about 20% for resolvable organic compounds [28].

More meaningful is the measurement of the particle composition in its natural environment, preferably at the single-particle level [29–31]. These real-time measurements allow particle-based characterization without any particle modification, but are typically not quantitative and suffer from poor mass resolution and mass accuracy in the case of mass spectrometry [32]. Both factors make the exact identification of the detected substances difficult and do not allow a detailed statement about the original amount of the often highly fragmented compounds.

The aim of this study was to combine the advantages of both methods, high-resolution atmospheric pressure laser desorption/ionization mass spectrometry and single-particle measurement, in order to identify as many substances as possible in the sample and to correctly assign them nearly on the level of individual particles. Another aspect was to reduce sample preparation compared to common analytical methods or to avoid it completely if possible. This was made possible by using a high-resolution mass spectrometer with an autofocusing imaging laser ablation source combined with a statistical evaluation of the image data. An approach for quantification of polycyclic aromatic hydrocarbons (PAH) with very low sample preparation via standard addition calibration is also shown. The samples were taken during winter at two heavily polluted megacities, Tehran and Hangzhou in Iran and China. Both cities are among the 500 most polluted cities in the world in 2018 according to the WHO [9].

## Materials and methods

### Particle sampling

Particles were sampled in Tehran (Iran) and in Hangzhou (China) on quartz filters in February 2018 for 2 days. The four samples were named as follows, FPI17B0807 and FPI17B0811 for Hangzhou on days 1 and 2, and 287 and 289 for Tehran on days 1 and 2. Sampling in Tehran took place at the Sharif University which is 500 m away from Sheikh Fazl-allah Nouri Expressway and 300 m from Yadegar-e-Emam Expressway, both heavily trafficked main roads in downtown. Sampling was done with a low-volume sampling device at 20 L/min for 24 h each. In Hangzhou, the filters were also sampled on consecutive days for 23 h at 16.67 L/min. The location of the sampling was on the rooftop of a company headquarters in a business park, a few kilometres south of Hangzhou downtown. The filters were weighed until a constant mass was obtained (< 0.04 mg). Filters were stored in a fridge at 5 °C and protected from light until the measurements took place (January 2019–July 2019).

### Filter measurements

Filters were cut into pieces, and particulate matter on the filters was directly measured with an AP-SMALDI5 AF imaging ion source (TransMIT GmbH, Giessen, Germany) on a Q Exactive HF mass spectrometer (Thermo Fisher Scientific GmbH, Bremen, Germany) at 50 µm pixel resolution in a mass range of *m*/*z* 50–750. For laser desorption and ionization, a diode-pumped solid-state laser at a wavelength of 343 nm with 50 pulses per pixel at 100 Hz was used. High-resolution MS imaging data of the filter surfaces at a mass resolution (R) of 240,000 in positive- and negative-ion mode were employed by using the autofocusing function of the ion source. The measured sample area for each filter was 37.5 mm^2^ for each polarity, corresponding to 150 × 100 pixels. Microscopic images of the measured versus unmeasured filter area and height information of the filter surface are shown in SI Fig. 1. For the quantification of PAHs, a dilution series of EPA 525 PAH mix A from Sigma-Aldrich (St. Louis, MO, USA), containing acenaphthylene (AcPy) at *m*/z 152.062; fluorene (Flu) at *m*/*z* 166.078; phenanthrene (PA), anthracene (Ant) at *m*/*z* 178.078; pyrene (Pyr) at m/z 202.078; benz[a]anthracene (BaA), chrysene (Chr) at m/z 228.094; benzo(b)fluoranthene (BbF), benzo[k]fluoranthene (BkF), benzo(a)pyrene (BaP) at *m*/*z* 252.094; benzo(g,h,i)perylene (BghiP), indeno(1,2,3-cd)pyrene (IND) at *m*/*z* 276.094 and dibenz(a,h)anthracene (DBA) at *m*/*z* 278.109 in dichloromethane, was prepared with concentrations of 43.03, 19.60, 3.90 and 0.37 µg/mL. Dried droplet application of 1 µL of each dilution, followed by drying for 1 h and consecutive mass measurement with the same ion source in a mass window of *m*/*z* 150–500 was carried out for the complete spotted area and additional area without a spotted standard for blank measurement.

### Data evaluation

For characterization of the filter samples, selection of *m*/*z* signals from the imaging raw data was conducted by our in-house developed software package MIRION at a bin size of 0.003 Da with a minimum image coverage of 0.05% [33]. A list of mass spectrometric features with maximum intensities for each *m*/*z* bin in each sample and for both polarities was extracted and filtered for signals above a relative threshold of 0.1% for negative ions and 0.4% for positive ions. Not all signals detected using the threshold criteria could be assigned to sum formulae based on mass deviation, Kendrick mass defect and isotope patterns. The signals that could not be assigned, mostly had intensities that were too low to detect corresponding isotope patterns and thus to use mass defects as criteria for assignment. Furthermore, the signals with very low intensities often showed strong variances in their mass deviation. The proportion of unassignable signals ranged from 1.4% for positively charged ions in Hangzhou to 6.6% for negatively charged ions in Tehran. Extraction of pixelwise intensities for the selected *m*/*z* signals was carried out by MSiReader at a mass window of ± 2.5 ppm [34]. Sum formula assignments for each signal were done manually via the XCalibur software (Thermo Fisher Scientific, San Jose, CA) based on measured mass, mass error and isotopic patterns (if available). For validation of mass assignments, additional criteria were used, such as Kendrick mass defect of CH_2_ and colocalization of sum formulas. A statistical approach was used to find colocalized signals that help to identify particles within the ablation area by correlating *m*/*z* signals. A hierarchical clustering (HCA) algorithm with *k*-means preprocessing within the Perseus software package (Version 1.6.14.0) allowed us to group signals that are found at the same positions on the filter surface [35]. Intensities were *z*-normalized for each assigned sum formula. The HCA was run 5 times to give a maximum of 50 data clusters for each filter and for positive- and negative-ion modes using complete linkage between the original data clusters. HCA was also used to find compounds that were statistically significant for each measurement location and independent of the sampling date. Therefore, a two-sample test was applied in positive- and negative-ion mode and the resulting compounds with *ρ*-values (probability that the means of two sample sets are significantly different) below 0.05 were used for clustering. Normalized mean intensities were used in this approach for each signal. To better illustrate the differences between cities, *Z*-score values were plotted based on the median for each significant *m*/*z* signal.

For quantification of the PAHs, the raw data was converted into imzml files and a region of interest (ROI) was selected manually for each deposited PAH concentration and one additional area was selected to determine the zero value and identify chemical noise signals. Total ion count (TIC) normalized intensities were extracted and calibration curves were calculated and evaluated using the standard addition technique. Results for each PAH signal were obtained by using the total particle mass and occupied area per filter relative to the ROI area. The ROI corresponded to only fractions of the total particle mass per filter, allowing quantification with less than 30 µg particle mass.

## Results

### Number and composition of compounds

**Fig. 1:**
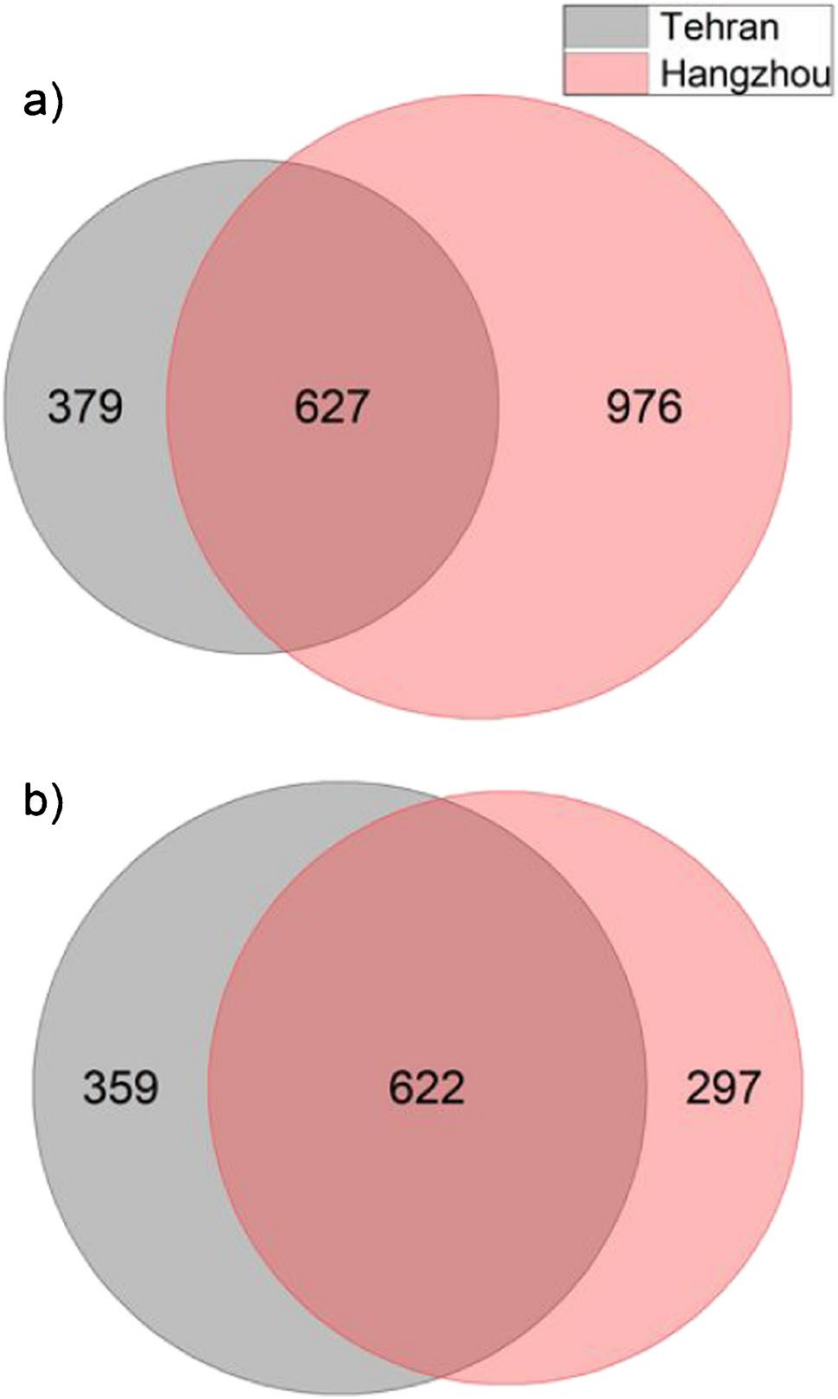
Total number of assigned sum formulae features represented in Venn diagrams for positive (**a**) and negative (**b**) ion mode

A total of 3258 m/*z* signals, excluding isotopic features of assigned signals, were assigned to all four filter samples collected on 2 days each in Hangzhou (China) and Tehran (Iran), 1981 in positive- and 1277 in negative-ion mode. Results are represented by Venn diagrams in Fig. 1. The particle mass concentrations were 43.1 µg/m^3^ and 37.5 µg/m^3^ for Tehran on days 1 (287) and 2 (289), respectively. Significantly higher concentrations were found in the Chinese samples with 174.7 µg/m^3^ and 106.5 µg/m^3^ on days 1 (FPI17B0807) and 2 (FPI17B0811). The annual mean value in Iran has been decreasing since 2012 and was 72 µg/m^3^ per year according to the WHO database [9]. Thus, the mass concentrations on our sampling days were significantly below the average. There are several possibilities why these concentrations might have been lower than the actual concentrations during sampling in Tehran. One possible reason is the time lag of several weeks between sampling and the final weighing in Germany. Another possible reason is the lower relative humidity in February in Tehran (57%) compared to Hangzhou (66%), which most probably led to a strong loss of volatile ammonium nitrate during the initial sampling [36, 37]. The significantly higher average humidity in Hangzhou combined with the direct weighing of the filters after sampling indicate that the measured concentrations were not subject to major changes during and after sampling and were thus close to the actual particle concentrations in the air. Studies show that the highest losses of ammonium nitrate occur below 60% relative humidity, while no loss is expected close to 100% [38]. Furthermore, a significant reduction of NO_3_^−^ ions from particulate nitrate in the presence of acid sulphates and ammonia in the collected particles can be expected, since the formed nitrate compounds are volatile. In the later course of this study, it becomes clear that due to strongly increased sulphate contents in the Tehran samples, this can lead to significant differences in nitrate content between the Chinese and Iranian samples. The annual mean concentration in Hangzhou fluctuated between 85 and 150 µg/m^3^ between 2013 and 2016, which is in the range of the concentrations measured in this study [9]. These particle mass concentrations indicate that particle pollution was higher in Hangzhou than in Tehran during the sampling period. This is also in line with the 27% higher number of detected signals and assigned sum formulas in the Chinese samples. In particular, 2522 sum formulas were assigned for both Chinese filters and 1987 for both Iranian filters. The Chinese samples showed a higher number of signals in the positive-ion mode (918, 1176) than in the negative-ion mode (690, 600), whereas for both Iranian samples the number of assigned sum formulae in the positive-ion mode (826, 568) was lower than in the negative-ion mode (892, 778). This was somewhat surprising, because LDI MS typically results in higher yields of positively charged than negatively charged ions, depending on the particle composition [39, 40]. The higher number of negatively charged ions in Tehran may have two reasons, most likely related to acidity and availability of anions. Negative charges arise mainly from capturing free electrons of photoionized analyte molecules in the plume or by the presence of substances with a high gas-phase basicity acting as a matrix that deprotonates inorganic and organic acids [41]. The second case is more likely and indicates that the samples from Tehran contain a higher amount of acidic compounds which can be easily deprotonated. The presence of these compounds is expected due to reactions of primary aerosols with sulphur dioxide and dimethyl sulphide, resulting in sulphuric acid–containing particles.

**Fig. 2:**
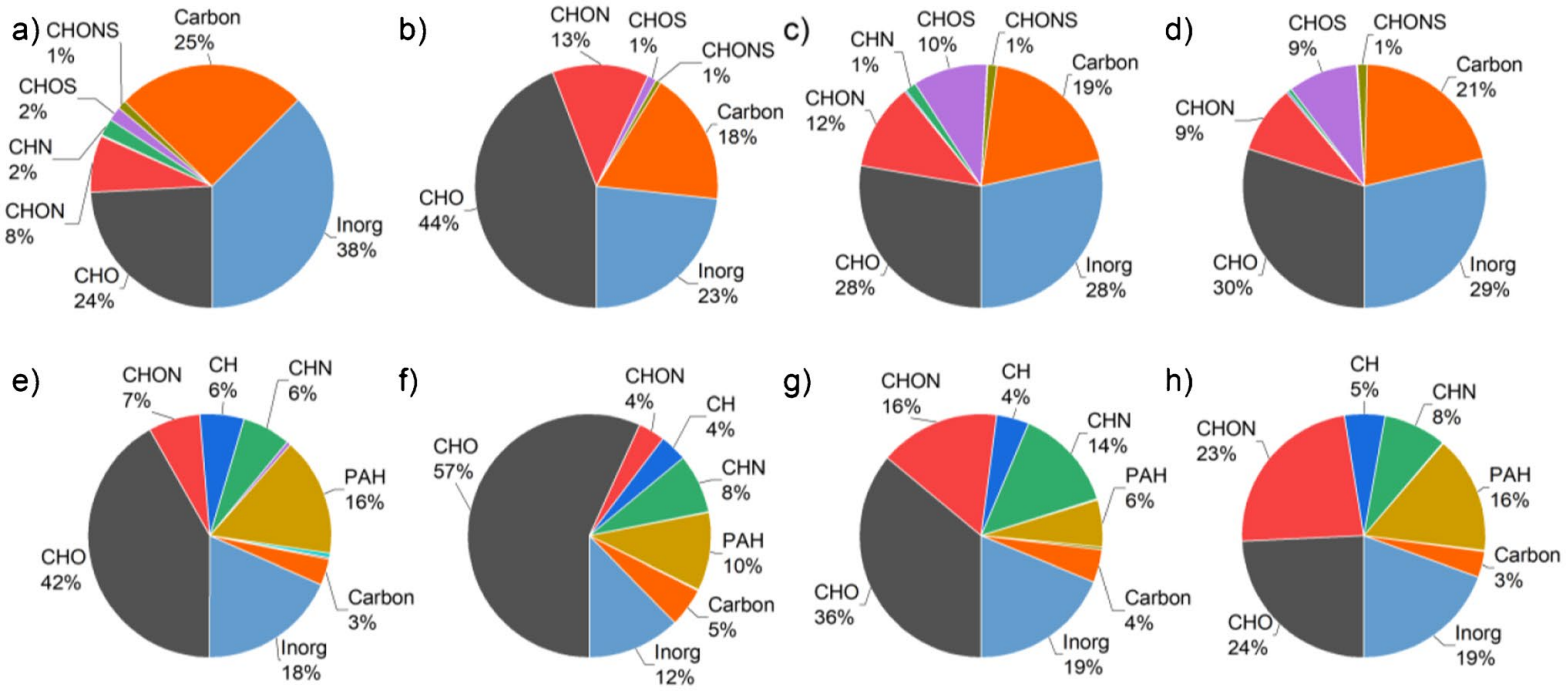
Assigned sum formulae grouped into 11 classes in negative- (**a**–**d**) and positive-ion mode (**e**–**h**). Measurements of the Chinese filters on day 1 (FPI17B0807, **a**, **e**) and day 2 (FPI17B0811, **b**, **f**) as well as for the Iranian filters on day 1 (287, **c**, **g**) and day 2 (289, **d**, **h**) exhibit characteristic features for each sampling location

Figure 2 provides an overview of all assigned sum formulae in positive- and negative-ion mode, divided into 11 categories, for both cities on the two sampling days. Here, we used the number of assigned signals instead of using intensity or peak abundance due to high uncertainties in ionization yield and behaviour of the different ions and classes of ions. The categories can be roughly separated into organic (CHO, CHON, CH, CHN, CHOS, PAH, CHNS, CHS, CHONS) and inorganic compounds (carbon, inorganic). Sum formulae containing carbon and hydrogen atoms were more frequently found in positive-ion mode, whereas negative-ion mode was more sensitive for heavy-metal-containing ions and acidic sulphate species. Deprotonation is known to favour the formation of carboxylate anions; thus, they are also found more frequently within the negatively charged ions. Although a similar amount of carbon black is found for all samples, its content within the negatively charged ions is much higher. The smallest variations within one polarity for both sampling locations were found in the carbon black content with relative numbers between 18 and 25% for the negatively charged ions (Fig. 2a–d) and significantly lower 3 to 5% (Fig. 2e–h) for the positively charged ions. If one takes the sum of the shares of these compounds (23 to 29%) for both polarities, the fluctuations between cities and sampling times were even smaller.

The most significant difference between the two cities was found in the amount of sulphur-containing organic compounds in negative-ion mode, which were very prominent in Tehran (11 and 11%) and almost absent in the samples from Hangzhou (3 and 2%). Since more than 90% of SO_2_ emissions in urban areas are caused by industrial processes, power generation and transportation, this dramatic difference can be attributed mainly to the SO_2_ air pollution control policy in China that strongly regulates sulphur dioxide emissions since 2000 [42]. After the first implementations of filter systems in the year 2006, the SO_2_ emissions declined until the recent years as shown by van der A et al. [43]. Larger differences were also found for CHON and CHN compounds in positive-ion mode, which seems to be more sensitive for those nitrogen-containing compound classes. Here too, the shares in Tehran with 30% and 31% relative assigned sum formulae number were more than double of those in Hangzhou with 13% and 12%. We assume that mainly traffic-related sources are responsible for these nitrogenous organic compounds, because the sampling in Tehran was carried out near a heavily frequented highway [44, 45].

As Fig. 2 illustrates, the inorganic compounds are responsible for more than 12% of all signals in the respective sample and polarity. This is the case in both positive- and negative-ion mode, whereas the proportion in negative-ion mode is higher with 23 to 38% compared to 12 to 19% in positive-ion mode. All inorganic species that have been assigned are characterized by the absence of carbon and hydrogen species in their respective molecular formulae. The majority of this ion class consists of alkali and alkaline earth metals, but a large number of heavy-metal-containing molecular formulae also belong to this group, which includes iron, copper, manganese, zinc, titanium, lead, chromium, nickel, arsenic, tin, cadmium, silver and cerium in decreasing order of relative content. Among them, only tin, silver and cerium have been detected as positively charged ions, while arsenic and cadmium in particular have been detected as negatively charged ions. Despite the overall lower number of assigned sum formulas in Tehran compared to Hangzhou, the number of assigned heavy metal species was higher in Iranian compared to Chinese samples by a factor of 1.2 and 1.7 for positively and negatively charged ions, respectively.

The total share of CHO-containing sum formulae ranges from 24 to 44% among the negatively charged ions and from 24 to 57% among the positively charged ions for both cities. The differences in aerosol composition between both sampling days, especially concerning CHO and inorganic species in positive-ion mode, were particularly remarkable in Hangzhou, whereas only minor differences were found in both samples from Tehran in all compound groups. In Hangzhou, a drastic change in particle composition was observed between days 1 and 2, especially for the negatively charged ions, starting from a strongly inorganic aerosol on day 1 (carbon + inorganic = 63%) to an organic-dominated aerosol on day 2 (carbon + inorganic = 41%). The relative number of CHO-containing species found in our study is in good agreement with other studies measured in south China and Singapore [46, 47]. Since we are able to detect and report other classes of compounds in addition to CHO compounds, such as large carbon fragments, inorganic compounds and PAHs, the relative proportion of CHO compounds is reduced compared to other studies [48]. If we count only organic compounds, the relative percentage of CHO-containing compounds rises to levels between 53 and 75%, as found in other studies [49].

In positive-ion mode, ion formation involves cationization and direct photoionization, leading to the formation of positively charged metal adduct and radical cations, respectively. It has been shown that PAH detection on carbonaceous compounds is supported by the higher UV absorption of elemental carbon. Due to the laser wavelength of 343 nm, unselective REMPI processes, in addition to the previously mentioned ion formation mechanisms, may also be responsible for the high number of PAH compounds detected in the positive-ion mode [50].

A large proportion of the polycyclic aromatic hydrocarbons (PAH) was detected in positive-ion mode and values for all samples ranged from 6 to 16%. Assigned substances belonging to this class included not only carbon- and hydrogen-based PAHs, but also those containing nitrogen (amino PAHs) and oxygen atoms (oxy-PAHs). While precursor PAHs from industrial processes, coal combustion and vehicle exhaust are mostly emitted as primary particles, their oxidation products provide evidence of aerosol ageing by photochemical processes or reactions with nitrate radicals, OH radicals and ozone [51]. Prominent representatives of these oxidation products are oxy-PAHs. To form oxy-PAHs from precursor PAHs, two conditions must be met: a sufficient time (typically a few hours) for the reaction to run its course and the presence of ozone or OH radicals [52, 53]. For fresh particles, the number of PAHs is expected to be higher than that of oxy-PAHs [54]. As an example, we chose benzo(a)pyrene (B[*a*]P, *m*/*z* 252.093), benz[a]anthracene (*m*/*z* 228.093) and pentacene (*m*/*z* 278.109) and their oxy-PAHs B[*a*]P-dione ([M + H]^+^ at *m*/*z* 283.075), benz[a]anthracenedione ([M]^•+^ at *m*/*z* 258.068) and pentacenedione ([M]^•+^ at *m*/*z* 308.083). All three PAHs were found in the Chinese and Iranian samples, but the corresponding oxy-PAHs could only be detected in the Chinese samples. This indicates that the Iranian samples were exposed to oxidizing compounds in the atmosphere for a limited time only, as these reactions are typically fast and occur within minutes to a few hours [55]. Reactions during sampling, storage and ionization can thus also be ruled out. Subdividing the PAHs into precursor and oxy-PAHs shows that the difference between Tehran and Hangzhou is mainly caused by the number of oxidized species, which is by a factor of 3.6 higher in the Hangzhou samples. Many of the detected CHN and CHON compounds are also potential PAH species, but have not been explicitly identified as PAHs. Since we found that the total particle concentration of the sample FPI17B0807 is 1.6 times higher than that of the sample FPI17B0811, despite the larger number of assigned sum formulae in the latter compared to the former, we assume that the high content of inorganic compounds contributes to the larger mass.

Most studies using ESI or other soft ionization techniques either for analysis of filter extracts or direct chemical characterization of aerosol particles on filters focus on protonated or deprotonated organic compounds [56, 57]. Here, we were able to detect inorganic molecules, inorganic carbon fragments and organic substances, which were either not detected within measurements using only a single analytical method in other studies or were not considered, but which are of great importance for the comprehensive interpretation of aerosol filter mass spectra. The increased number of nitrogenous and sulphur compounds in Tehran compared to Hangzhou is in line with regional particle sources such as traffic in the first case, and the overall increased pollution from unfiltered industrial waste gases in the second case. This shows that, when measuring with only one ion polarity, an extremely large number of compounds, including PAHs, certain heavy metal species, sulphates and CHN compounds can only be determined partially or not at all.

### Double-bond equivalents of detected organic compounds

**Fig. 3:**
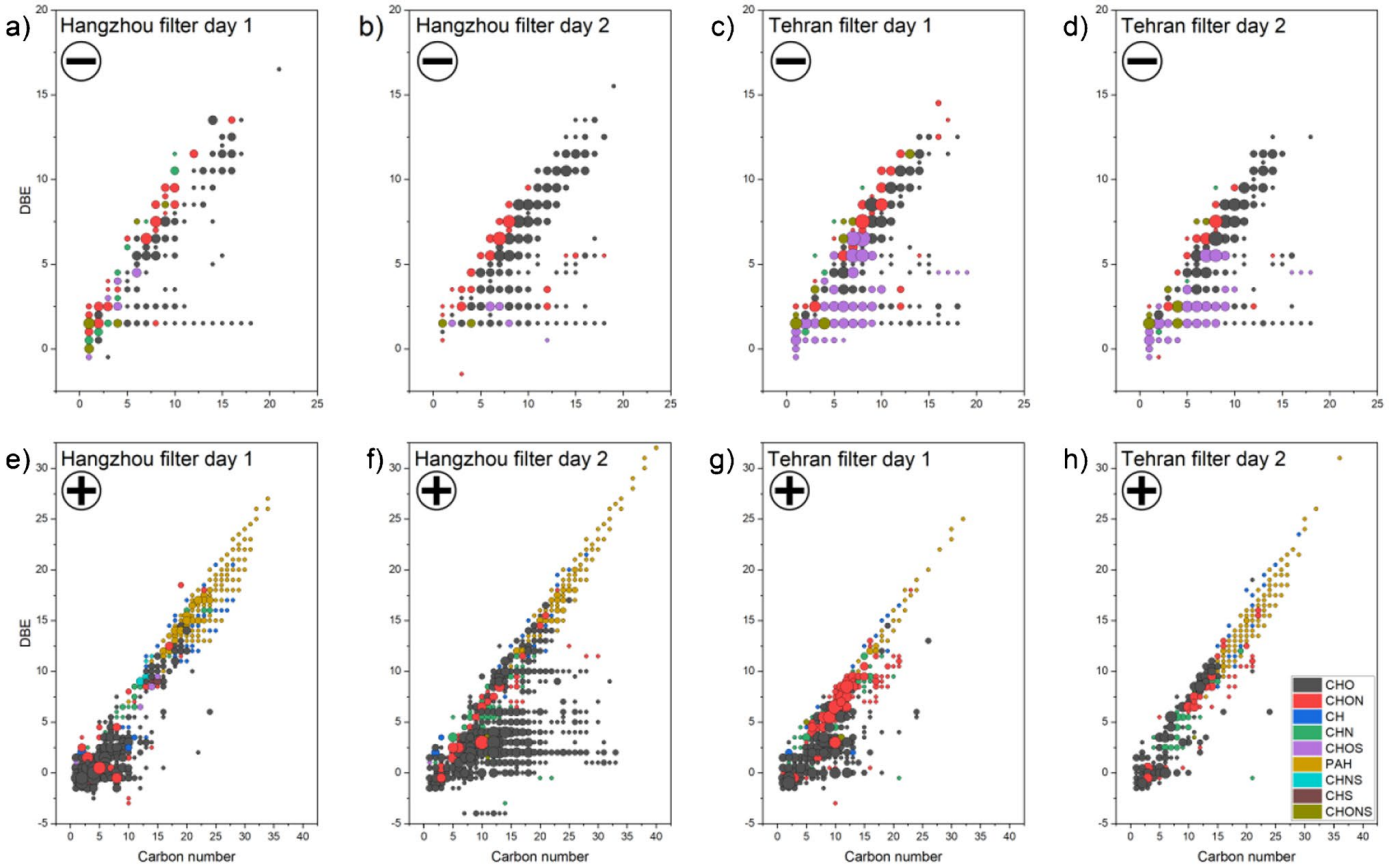
Plots showing the number of double-bond equivalents for 9 classes of organic molecules in negative- (**a**–**d**) and positive-ion mode (**e**–**h**). The Chinese filters on day 1 (FPI17B0807, **a**, **e**) and day 2 (FPI17B0811, **b**, **f**) as well as the Iranian filters on day 1 (287, **c**, **g**) and day 2 (289, **d**, **h**) are shown. The area of each data point corresponds to the number of compounds

The double-bond equivalents (DBEs) of organic molecules is a structural descriptor that helps to separate compounds into groups. For example, oxidized compounds or aromatics group together based on DBEs. From this, the ageing behaviour of particles can be assessed, among other things. Figure 3 shows DBEs for each associated molecule categorized as CHO, CHON, CH, CHN, CHOS, PAH, CHNS, CHS or CHONS. The number of carbon atoms is plotted against the number of DBEs and the number of matching molecular formulae is indicated by the area of the data points. In general, the number of DBEs of all compounds was higher in the Hangzhou samples compared to the Tehran samples.

Especially, the samples from Tehran showed very similar horizontal and vertical distributions for the same ion classes in the negative-ion mode (Fig. 3c and d). Primarily, sulphur-containing compounds in the form of CHOS were observed in the two Tehran samples, but mostly for DBEs below 7. In the same carbon number and DBE regions, mainly CHO compounds were detected in the samples from Hangzhou, but in lower relative quantities than the sulphur compounds in Tehran. The class of CHO compounds was predominantly grouped above a DBE of 5 for the negatively charged ions and below 10 for the positively charged ions. Apart from this, the patterns of negatively charged molecules were similar in both cities, except for sample FPI17B0807 in Fig. 3a. Here, we found lower relative numbers of all compounds, especially in the region between 9 and 13 carbon atoms. The positively charged ions were somewhat more meaningful, mainly due to the assigned PAHs. These showed the highest number of DBEs.

An average DBE was calculated for the different classes, from which the unsaturation could be estimated and compared between the two megacities. Only the most prominent classes with more than 10 compounds were used for comparison in order to obtain appropriate statistics. The evaluation was performed for positive-ion mode. For CHO compounds, the average DBEs of both cities were similar, with DBE_avg_(Hangzhou) = 1.9 and 3.5 and DBE_avg_(Tehran) = 2.1 and 3.5 for days 1 and 2, respectively. Differences were found for the class of CHON compounds, with increased unsaturation observed in Tehran (DBE_avg_ = 6.3 and 6.4) compared to Hangzhou (DBEavg = 3.0 and 6.5), indicating higher oxidation of nitrogenous compounds, implying a contribution from car exhaust gases.

For the more hydrocarbon-based structures such as PAH and CH compounds, the situation changed due to the higher unsaturation of both classes in Hangzhou compared to Tehran. Especially, the PAHs from Hangzhou were found to have significantly higher DBEs (DBE_avg_(Hangzhou) = 16.1 and 16.5, DBE_avg_(Tehran) = 13.0 and 14.2). This may be a consequence of a longer transport of the PAHs in the atmosphere. It has been shown that with the higher molecular weight of the PAHs and higher unsaturation, stronger adsorption to the particle phase is associated, which allows these compounds to remain longer in the atmosphere [58, 59]. This observation is in good agreement with the higher oxidation observed for the Hangzhou PAH fraction. As a result, the PAHs found in Hangzhou may come from more distant sources like coal combustion, remote traffic and other industry. Sample FPI17B0811 (Fig. 3b and f) also showed that there is a trend towards higher DBE if the number of CHO compounds rises. The lower-molecular-weight PAHs in Tehran may have different sources, but these were confined to the immediate vicinity of the sampling point, and were thus mainly restricted to car exhaust. The gap between DBE 5 and 8 of the Chinese sample from day 1 is mainly due to the lack of CHO and CHON species with corresponding DBEs.

### Distinguishing between Tehran and Hangzhou on the basis of characteristic compounds

The samples from Tehran and Hangzhou were distinguished by statistical evaluation of *m/z* signal identities and intensities, as shown in SI Fig. 3. For this purpose, 168 and 186 significant compounds were identified for negative- and positive-ion mode by means of a Student t-test and clustered using HCA, respectively. SI Fig. 3 shows that by using these significant compounds, both cities can be easily separated, due to either very low or zero compound intensities (blue colour) or high compound intensities (red colour). The individual sum formulas are not easily readable due to their large number and are therefore listed in detail in SI Table 3.

In the case of the negatively charged ions in SI Fig. 3a, few compounds were found that were characteristic for the Hangzhou samples. These few compounds included mainly inorganic nitrate clusters with 3 to 5 nitrogen and 9 to 15 oxygen atoms. These compounds can be associated with NO_x_ emissions from traffic-related sources, but may also result from the reduced SO_2_ emissions in China [60, 61]. Low SO_2_ conditions favour the formation of secondary inorganic aerosols (SIAs) with high nitrate content. This is typically observed when the gaseous nitrate is converted to HNO_3_ and neutralized by atmospheric ammonia NH_4_^+^ [62]. These secondary inorganic nitrate particles are found several kilometres from their source due to their long atmospheric lifetime and small size [63]. Furthermore, NH_4_NO_3_ evaporates significantly (> 80% at 35 °C and 18% rel. humidity) by sampling in a dry and warm environment as in Tehran, and can additionally react with sulphuric acid to form HNO_3_ (g) [64, 65]. In both cases, the nitrogen content in the particle phase in Tehran will consequently be reduced and significantly lower amounts were detected, while the conditions in China would lead to much lower losses of particulate nitrate.

The characteristic negatively charged ions from Tehran samples included a large variety of different inorganic and organic species. From these, 76 out of 159 compounds contained sulphur atoms which makes the sulphur content the best distinguishing feature between Tehran and Hangzhou. Some silicates, organic nitrates as well as inorganic copper, iron and zinc compounds were found as well.

For the positively charged ions in SI Fig. 3b, mainly high molecular PAHs and oxy-PAHs, some CHN species and inorganic calcium were found to be characteristic for Hangzhou, while Tehran samples were dominated by lower-molecular-weight PAHs, CHON species and inorganic chromium compounds. Until now, the data allows for a differentiation of the two cities based on the bulk particle composition of the whole filter. However, by looking at a very restricted area on the filter surface and extracting the chemical information for this area, as shown in the following chapter, the characterization of individual particles can be approached.

### Particle-based classification

LDI imaging as a technique for untargeted analysis of aerosol filter samples under atmospheric pressure is not only capable of characterizing filter samples as bulk material but also allows for the characterization of small communities of particles. This is necessary to evaluate the composition of single particles or particle groups in terms of source apportionment and mixing state. For this approach, the location and composition of each mass spectrum in the imaging data set is correlated with each other on a statistical basis. Sum formulae on the filter surface are grouped based on their location and signal intensity. As a result, different particle classes (data clusters) within groups of particles were identified. These identifications of particle classes on filter samples have so far been possible mainly on the basis of classes of molecules with structurally similar properties, such as lipid species with different head groups, carbohydrates, alkanes and terpene compounds, determined by chromatographic MS techniques. Identification, based on the association of structurally very different compounds in the same particle, such as those occurring in highly internally mixed aerosol particles, has been rather challenging [66]. A major advantage of the method is that both inorganic and organic compounds can be detected and that both can occur in the same data cluster and, thus, in a small group of particles. Conclusions about the mixing of different chemical components in the particles, their ageing or the composition of the underlying primary particles (mineral, black carbon or organic) are thus possible.

In addition, the distribution of single particles on the filter surface, agglomeration effects and the shape of larger particles is also accessible with the method. Since the laser focus diameter was about 50 µm, there were certainly several particles assembled within this ablation area, which were ionized simultaneously and thus produced a mass spectrum corresponding to a small group of particles and not to a single particle. If, however, very specific *m*/*z* signals repeatedly occur together in certain spectra, the probability that these signals belong to individual particles rather than to particle agglomerates increases with the number of spectra measured.

The size and composition of a data cluster was one criterion to explain particle transport, composition and source. Overall, there was a larger number of small clusters (< 10 compounds) observed in the negative-ion mode compared to the positive-ion mode. From a chemical point of view, the smaller data clusters in negative-ion mode mostly consist of inorganic compounds containing elements such as iron, titanium, copper, silicon, phosphate and sulphate while in positive-ion mode, smaller CHO, CHON and CHN clusters were found more frequently. The smaller inorganic data clusters are mainly due to metal abrasion and mineral fragments, as there is little mixing of different metal species with organic compounds. The organic components that were found in such small data clusters are often linked by consecutive losses of C, H and/or O from a parent compound.

**Fig. 4:**
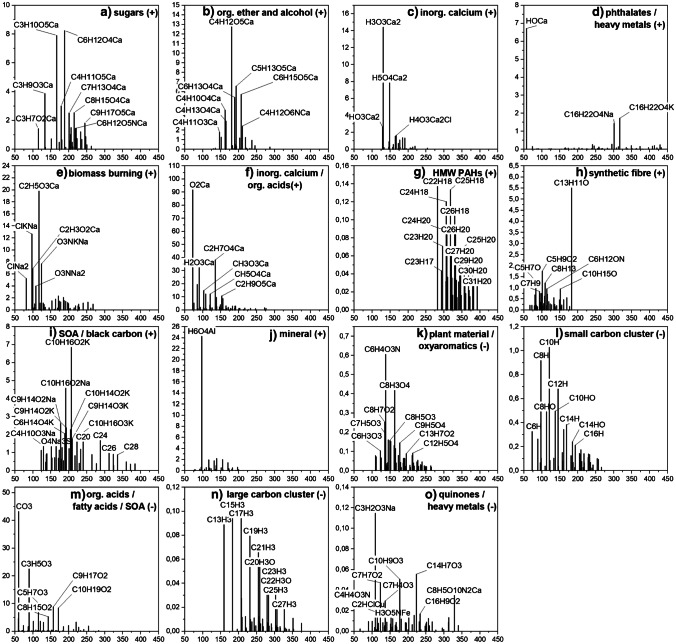
Representative mass spectral patterns for larger data clusters (> 30 sum formulas) for the Hangzhou sample FPI17B0807. A total of 15 data clusters, 10 for positively charged ions (**a**–**j**) and 5 for the negatively charged ions (**k**–**o**) were found and assigned to different particle types

**Fig. 5:**
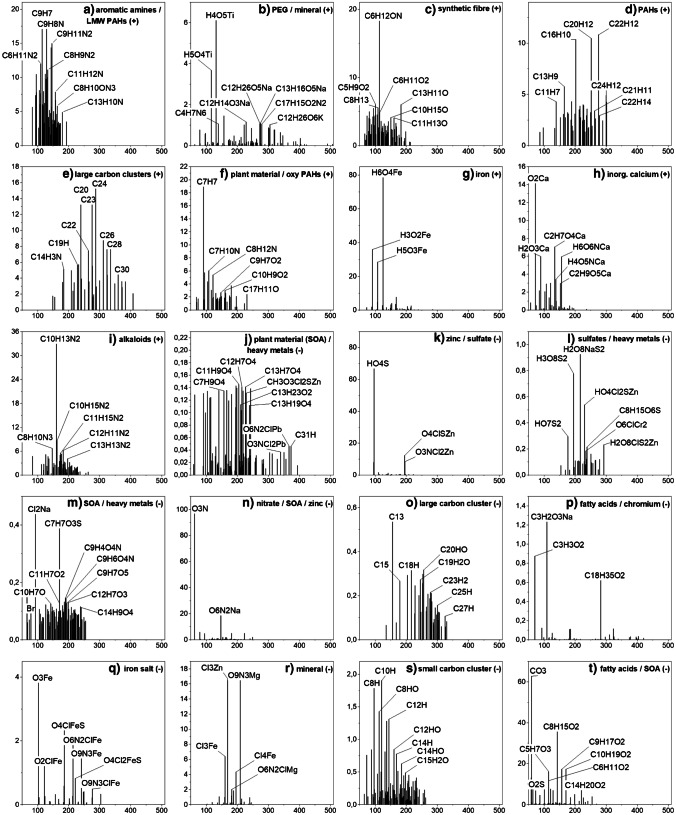
Representative mass spectral patterns for larger data clusters (> 30 sum formulas) for the Tehran sample 287. A total of 20 data clusters, 9 for positively charged ions (**a**–**i**) and 11 for the negatively charged ions (**j**–**t**) were found and assigned to different particle types

Larger data clusters indicate internally mixed particle groups or primary particles consisting of mixtures of different substances. For example, PAH particles, which generally contain a larger number of different low- and high-molecular-weight PAH species, are more common in positive-ion mode. It is expected that during the chemical conversion of compounds and the adsorption of new compounds, larger data clusters containing a larger number of signals will be formed. From these larger data clusters, the evolution of the underlying particles can be traced, so that an assignment of the particle source, the particle age and its transformation in the atmosphere is possible. For larger data clusters (> 30 compounds), representative spectral patterns were obtained for the sample FPI17B0807 from Hangzhou (Fig. 4) and the sample 287 from Tehran (Fig. 5). Both samples showed the highest particle concentrations and were selected on this basis. Shown are averaged intensities of signals that were assigned to the respective data cluster. The structure of the clusters was also used to verify the plausibility of individual sum formulae, for example if they contained the same atoms or molecular fragments as other ions of the same cluster. This helped to identify the data clusters and name them according to the assigned compound classes.

All measured samples had a number of specific data clusters in common. Prominent representatives here are mainly black carbon, mineral clusters and PAHs. Black carbon, which is produced by the incomplete combustion of fossil fuels, biomass and biofuels is largely found in both cities. The data clusters in Fig. 4i, l and n as well as in Fig. 5e, o and s show typical patterns of carbon fragments, divided into data clusters with low-molecular-weight (C_4_–C_22_) and higher-molecular-weight (C_13_–C_34_) species. This suggests that the carbon chains of different lengths are grouped together in different particles and subsequently may come from different sources. Mineral clusters in Figs. 4j and 5b, r mostly contain a mixture of the elements Al, Ca, Fe, Mn, Mg, Si and Ti. Data clusters of PAHs were found in both the Hangzhou sample and the Tehran sample but patterns of PAHs in the spectra differ. Whereas the clusters in Figs. 4g and 5d contain only PAHs, the data clusters in Fig. 5a and f show mixtures of PAHs with amines and plant compounds. Mainly high-molecular-weight species of PAHs were found in Hangzhou and low-molecular-weight species in Tehran. The data cluster with aromatic amines in Fig. 5a is of particular interest, because these compounds are very often classified as carcinogens and arise mainly from coal combustion activities in the respective region, but can also be attributed to cigarette smoke [67, 68]. Therefore, low-molecular-weight PAH compounds found in this data cluster can also be derived from these kinds of sources but are typically present in traffic emissions as well [69]. Especially, smaller ions like C_9_H_7_^+^ may also be fragments of larger PAH species like substituted naphthalenes, which was shown by Koptyug et al. [70]. Mixed clusters of heavy metals and sulphate (Fig. 5l), as well as zinc and sulphate (Fig. 5k) are also likely to be due to the combustion of coal. These observations are in good agreement with the higher number of heavy-metal-containing clusters (esp. sulphates and chlorides in negative-ion mode) in Tehran in general (Fig. 5j, k, l, m, n and p). None of the larger data clusters in Hangzhou in Fig. 4 are dominated by heavy metals such as Zn, Pb, Ni, Cr and As, associated with strong adverse health effects [71, 72]. These toxic elements only play a minor role in data clusters 4 d and 5 o. Figure 4d also shows intensive signals of phthalates, which are ubiquitous and cannot be assigned to any specific source.

Plant materials such as sugars (Fig. 4a), alkaloids (Fig. 5i), fatty acids (Figs. 4m and 5p, t) and other biogenic volatile organic compounds (Fig. 4k and 5f, j) were found as components of a variety of data clusters. While in Hangzhou there were only three data clusters associated with plant material, which also showed little mixing with other substances, in Tehran plant material was found in five data clusters and usually highly internally mixed. Secondary organic aerosols (SOA) were often part of these data clusters, but in both Hangzhou and Tehran samples, they were usually mixed with black carbon (Fig. 4i), fatty and other organic acids (Figs. 4m and 5t) and heavy metals (Fig. 5j, m, n). SOA were characterized by a number of oxidized terpenes and ketoaldehydes like camphoric acid (C_10_H_16_O_4_), 2.3-dihydroxy-4-oxopentanoic acid (C_5_H_8_O_5_), phthalic acid (C_8_H_6_O_4_), 4-methyl-5-nitrocatechol (C_7_H_7_NO_4_), succinic acid (C_4_H_6_O_4_), cis-pinonic acid (C_10_H_16_O_3_) and pinic acid (C_9_H_14_O_4_), all of them identified as markers of SOA in numerous in-situ and smog chamber studies, in both ozone and OH oxidation reactions [73–77]. All these compounds were found as [M-H]^−^ ions, except pinonic acid, which was also detected as [M + K]^+^ in the data cluster in Fig. 4i.

Calcium was one of the main components found in the Hangzhou sample, it occurred in inorganic and organic compounds and generally favored the formation of positively charged ions. Since calcium was also the main component responsible for the high inorganic content of this sample, we assume that a large proportion of mineral dust in the form of calcium carbonate particles specifically contributed to the formation of this aerosol. Typical sources of calcium carbonate are desert sand particles and sea spray aerosols [78, 79]. Two data clusters dominated by calcium compounds are shown in Fig. 4c, consisting of pure inorganic calcium and Fig. 4f, with a mixture of small organic acids and calcium. One data cluster from Tehran in Fig. 5h was likewise composed of inorganic calcium, but the remaining data clusters showed only few calcium-based compounds.

A very interesting feature in both cities were two data clusters (Figs. 4h and 5c), which contained a number of compounds whose molecular formulas can be assigned to caprolactam (C_6_H_11_ON) and methyl methacrylate (C_5_H_8_O_2_). These two prominent compounds are mainly associated with synthetic fibres, such as nylon and other plastics. Despite their use as synthetic fibres, caprolactam is used in many industrial manufacturing activities and has been found in PM_2.5_ aerosols in a Canadian study in significant levels as well [80].

Some data clusters were found to be specific for the respective city. Among the calcium-rich clusters in Hangzhou and the heavy-metal-containing clusters in Tehran, the data clusters in Fig. 4b and e are particularly noteworthy for the Chinese location. While the former consists mainly of smaller organic compounds such as ethers and alcohols and has no specific characteristics, the latter is clearly characterized by sum formulas associated with levoglucosan (C_6_H_10_O_5_), mannose and mannitol (C_6_H_14_O_6_), all markers of biomass combustion. Levoglucosan was found in the sample from Tehran as well, but mixed with SOA and heavy metals in data cluster m. Levoglucosan ions were detected as [M + K]^+^, [M + Na]^+^ and also [M-H]^−^.

In Tehran, we found two data clusters, one in positive- (Fig. 5g) and one in negative-ion mode (Fig. 5q), that included mainly inorganic iron in the form of water clusters as positive ions and in the form of nitrates, sulphates and chlorides as negative ions. Both were likely arising from combustion processes emitting iron oxide particles which are transformed in the presence of SO_2_, HCl and NO_x_ to form the observed compounds. Weber et al. [81] identified iron emissions from waste incineration and coal power plants.

Many of the identified data clusters are in good agreement with organic aerosol classes found for the Chinese megacity Nanjing [66]. Some studies show that it is possible to differentiate between different stages of oxygenation and carbon numbers, for example by aerosol mass spectrometers, but their interpretations are restricted to mainly fragments of compounds rather than intact parent ions [82, 83]. Rivellini et al. [47] differentiated less oxidized oxygenated organic aerosol and more oxidized oxygenated organic aerosol by ratios of C_2_H_3_O^+^ plus CO_2_^+^ to CO_2_^+^ on a quantitative basis. They also pointed out that by measuring additional refractory metals, attributing particle sources becomes more reliable compared to our approach. Using mixtures of metal ions and organic parent compounds grouped together in our data clusters allowed us to differentiate more easily between different sources. This was particularly meaningful in the case of terpenes for secondary biogenic organic aerosol, or levoglucosan mixed with small sodium and potassium ions in case of biomass burning. Especially, the occurrence of heavy metals mixed with PAHs and SOA in the samples from Tehran is of concern. A larger number of signals containing chromium, lead and arsenic were found in the majority of data clusters from Tehran. Especially, zinc and chromium, which were both data clustered together with the majority of CHOS compounds in Fig. 5k and l, clearly show that the emission of organic sulphur compounds is accompanied by an increased amount of toxic heavy metals. Some of the substances alone are already harmful to health, while a mixture of different sources was found here. The high quantities of harmful substances found show that negative health consequences can be expected, especially on days with very high particle loads. For some of the PAHs, we implemented a quantification approach, which is shown in the following section.

### Quantification of PAH species

**Fig. 6:**
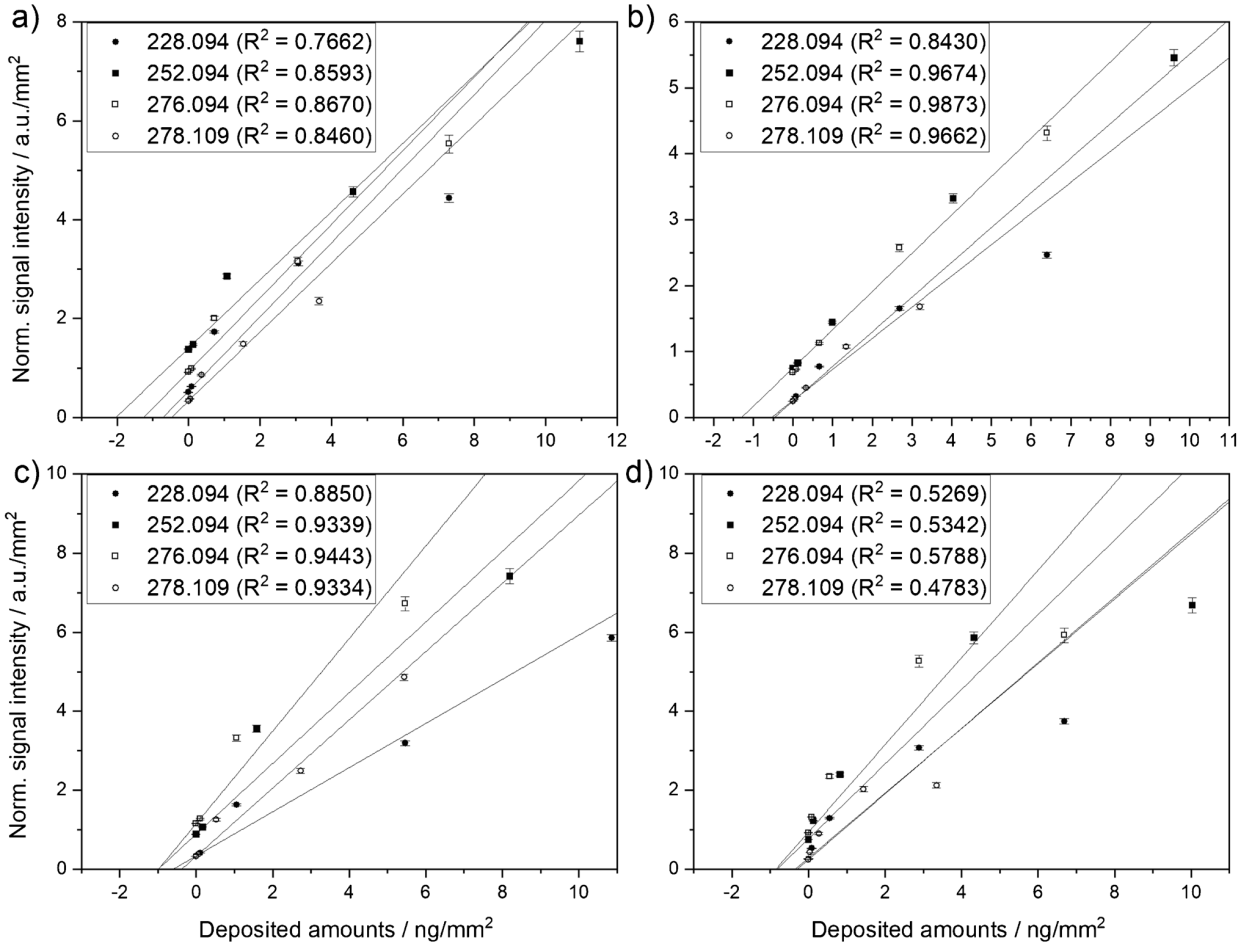
Calibration curves for dried droplet standard addition method on Hangzhou samples FPI17B0807 (**a**), FPI17B0811 (**b**) and Tehran samples 287 (**c**) and 289 (**d**). Calibration curves are shown for high-molecular-weight PAHs

Concentration series of 13 PAH compounds in dichloromethane were prepared and 1 µL of each dilution was spotted on the sample surface using the dried droplet method. Rasterizing the surface, followed by normalization to TIC allowed us to retrieve calibration curves with accuracies *R*^2^ ≥ 0.85 in 63% of all measurements as shown in Fig. 6 and SI Fig. 2. A few assumptions were made for the calculations. Since a standard solution suitable for LC or GC was used, containing also several PAHs with identical masses that can be separated chromatographically but not mass spectrometrically, it was assumed that each species with identical mass contributed equally to the intensity of the mass signal, meaning that these species have the same ionization and desorption properties on the filters. Due to this assumption, we list total concentrations of PAHs per mass and not per PAH species. Calibrations curves are shown in Fig. 6. Differences in ionization yields and signal intensities between the PAH species are expected due to their different vapour pressures, since the main reason for the ablation and desorption process is the explosive thermal decomposition of the carbon particles due to overheating [84]. However, this is true for all PAHs to the same extent, since the conditions and the substrate are homogeneously distributed.

Using the standard addition method, determined concentrations of low-molecular-weight PAHs (SI Fig. 2) were partly above 5000 µg/g and were thus significantly higher than high-molecular-weight species (181.5 to 1235 µg/g) (Fig. 6) for the Hangzhou and Tehran samples (Table 1). This effect is probably due to generally lower ion yields of low-molecular-weight compounds causing higher inaccuracies for the calibration curves but may also be the result of a calibration artifact, caused by the higher vapour pressure of the low-molecular-weight PAH species combined with a higher specific surface area of small particles: lowly concentrated solutions of low-molecular-weight PAHs will adsorb on the high specific surface area of the small particles and only a small fraction will thus evaporate from the filter surface. With rising concentrations, a higher fraction of the low-molecular-weight PAHs will escape into the gas phase as non-surface-bound volatile substances. As a result, the lowly concentrated solutions of the highly volatile PAHs are found to have higher levels in the particulate phase than the higher concentrated solutions, as most of them have already entered the gas phase. This effect reduces the slope and linearity of the calibration curves and in our measurements resulted in a significantly too high sample concentration. This can also be seen in the *R*^2^ values, which improved for the higher-molecular-weight PAH species compared to the low-molecular-weight species (see SI Fig. 2) from roughly 0.2 to 0.9. The lower the vapour pressure of the compounds, the weaker this effect became. Sheu et al. [59] showed that this effect is particularly pronounced with 2- to 4-ring PAHs, as these are present in the gas phase to about 4 to 50%, while the proportion of PAHs in the gas phase in species with more than 5 rings is less than 1%. For our study, the vapour pressures of the measured PAHs rise significantly between the three PAHs BaA and Chr at *m*/*z* 228.094 and Pyr at *m*/*z* 202.078 by one to three orders of magnitude, as can be seen from literature values (*P*_BaA_ = 2.1 × 10^−7^ mm Hg, *P*_Chr_ = 6.23 × 10^−9^ mm Hg and *P*_Pyr_ = 4.5 × 10^−6^ mm Hg) [85, 86]. Therefore, it is not surprising that for all PAHs heavier than *m*/*z* 202.078, we obtained values that are very comparable to literature values, while the lighter PAHs (*m*/*z* < 202.078) showed concentrations that were clearly too high. We therefore focused on the higher-molecular-weight PAHs between *m*/*z* 228.094 to 278.109 in our study.

**Table 1:** Results from standard addition calibration of PAH species for the two filters from each city. Concentrations of higher-mass PAHs > 202.078 were found to be much more reliable due to their lower vapour pressure

Sample	PAH content/µg/g							
	152.062 (AcPy)^*^	166.078 (Flu)^*^	178.078 (PA, Ant)^*^	202.078 (Pyr)^*^	228.094 (BaA, Chr)	252.094 (BbF, BkF, BaP)	276.094 (BghiP, IND)	278.109 (DBA)
FPI17B0807	4187 ± 2601	1174 ± 237	657 ± 65	714.3 ± 115	270 ± 77	766 ± 159	470 ± 94	182 ± 39
FPI17B0811	5654 ± 623	5395 ± 2789	1227 ± 216	752 ± 106	334 ± 76	798 ± 75	668 ± 39	289 ± 28
287	N. D	10,271 ± 689	2792 ± 476	531 ± 105	758 ± 154	1235 ± 170	1222 ± 152	478 ± 69
289	80,556 ± 148,020	6776 ± 2998	2602 ± 481	1270 ± 339	504 ± 229	1168 ± 504	1216 ± 482	431 ± 203

^*^Reported values are too high due to intrinsic properties of the PAHs resulting in large calibration errors

Linearity for measurement of sample 289 (Fig. 6d) was worse compared to the other samples, but in general we found very good correlations, mostly with *R*^2^ > 0.9. In general, the two samples from Tehran showed elevated concentrations of PAHs compared to both filters from Hangzhou, as shown in Table 1.

We found comparable concentrations of all PAH species within one city which indicates that the method is properly reproducible within the two samples measured. However, concentrations in Tehran samples were on average about twice as high as in Hangzhou samples. In particular, PAHs BbF, BkF, BaP (1235, 1168 µg/g) and BghiP, IND (1222, 1216 µg/g) showed very high total concentrations in samples 287 and 289 from Tehran. Those were lower than reported by Sheu et al. in ambient air at a traffic site at National Cheng Kung University (NCKU) (Tainan, Taiwan) with 3003.8 µg/g and 4407.2 µg/g for BbF, BkF, BaP and BghiP, IND, respectively, but their measurements were performed during the 1990s, when the exposure in China was at its peak [59, 87]. Levels of BaA and Chr (758 µg/g, 504 µg/g) and DBA (478 µg/g, 431 µg/g) were in the same order as those from Sheu et al. [59] with 403.03 and 565.2 µg/g. These values are alarmingly high, especially if the low particle concentration of the samples from Tehran is taken into account. Comparing our present results for China show, that even though the particle mass was twice as high in both Hangzhou samples compared to Tehran, the PAH concentrations were 37 to 54% lower. Concentrations between both Hangzhou samples varied between 4 and 37%, wherein FPI17B0807 was lower than FPI17B0811.

**Fig. 7:**
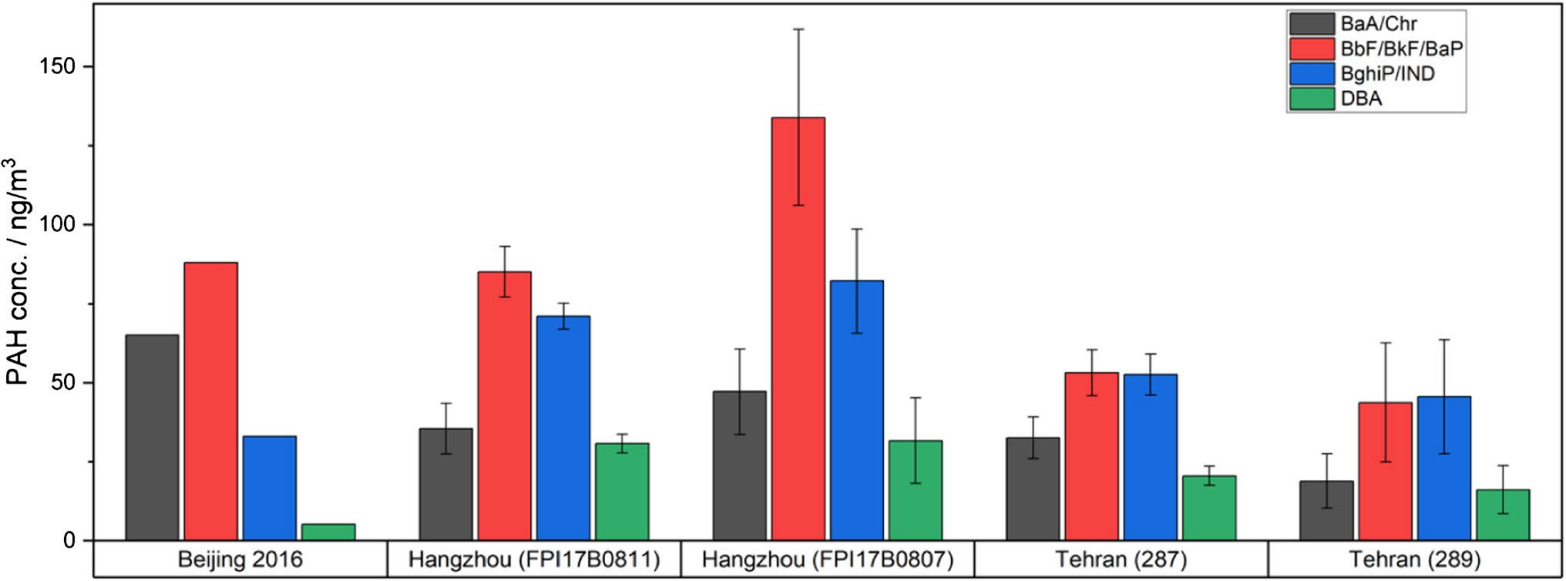
Calculated concentrations of 4 selected PAH groups BaA/Chr, BbF/BkF/BaP, BghiP/IND and DBA for each Tehran and Hangzhou sample compared to values measured in Beijing during winter 2016 [88]

In order to compare our results from Hangzhou and Tehran with recent studies, we calculated the PAH concentration per sampled volume of air. Measurements in Beijing in winter 2016 revealed similar maximum levels of PAHs at a 3 h average as we found in Hangzhou (Fig. 7) [88]. Concentrations were 65.2, 88.0, 33.1 and 5.2 ng/m^3^ for BaA/Chr, BbF/BkF/BaP, BghiP/IND and DBA, respectively. Which is in the range of concentrations we measured for FPI17B0811 (35.5 ± 8.1, 85.0 ± 8.0, 71.1 ± 4.1 and 30.8 ± 3.0 ng/m^3^) and FPI17B0807 (47.2 ± 13.5, 133.9 ± 27.9, 82.2 ± 16.4 and 31.7 ± 13.5 ng/m^3^). Due to much lower particle concentrations in the air, the total PAH concentrations for Tehran were lower with 32.6 ± 6.6, 53.2 ± 7.3, 52.6 ± 6.5 and 20.6 ± 3.0 ng/m^3^ for sample 287 and 18.9 ± 8.6, 43.8 ± 18.9, 45.6 ± 18.1 and 16.2 ± 7.6 ng/m^3^ for sample 289. The concentration of BbF/BkF/BaP was elevated in Hangzhou compared to other PAHs, while BbF/BkF/BaP and BghiP/IND were almost equal in both samples in Tehran. Compared to the values of Elzein et al. [88], the samples from Tehran and Hangzhou show a significantly higher concentration of the higher-mass PAH DBA.

The previously observed increased pollution of samples from Tehran, particularly by heavy metals and organic compounds, is confirmed by this quantitative approach. It becomes clear that the total pollution cannot be deduced directly from the particle concentration, since the composition of the particles is the most important factor. It is mainly small organic and soot particles that contribute to the significantly high pollution of the air with carcinogenic and toxic substances in Tehran.

## Conclusion and discussion

Ambient LDI MSI was used for the first time to unravel the chemical composition of aerosol particles collected on quartz fibre filters. An autofocusing MALDI MSI source was used to compensate for the extensive surface roughness of the filter and allowed for constant and high ion signals throughout the whole ablated area. An ultrahigh-resolution orbital trapping mass analyzer was used to differentiate very small differences in *m/z* values and to assign more than 3200 m/*z* signals with chemical sum formulas in positive- and negative-ion mode for two filters from Iran and China, respectively, quite a number of them as intact molecular compounds instead of fragments, as would be expected based on the ionization method. The addition of a matrix as in MALDI experiments was not necessary, as particle absorbances were sufficiently high in general and particularly PAH ionization was supported by the higher UV absorption of elemental carbon present on the filters [89]. Since our approach, using scanning laser desorption/ionization mass spectrometry, is an offline technique where particles are collected, stored and brought to the laboratory, particle compositions may change by chemical reactions or evaporation before analysis.

The lower number of sulphur compounds detected in Hangzhou may indicate a successful Chinese sulphur dioxide emission policy. This led to a significant reduction of sulphuric acid contents in the particles. As the proportion of nitrogen oxide compounds was also lower in our measurements, it can be assumed that the exposure to nitric acid and its reaction products was also reduced. Since it has been shown that the influence of acidic sulphur compounds can lead to a reduction in the number and concentration of nitrate-containing compounds on filter samples, the actual difference is probably even larger [38]. It is alarming that, despite the mass loadings of the filters from Tehran were by a factor of 3 or more lower than in Hangzhou, the number of heavy-metal-containing compounds was up to 1.7 times higher and PAHs showed nearly double the concentrations as in Hangzhou. This raises concerns about the exposure of the population in Tehran to toxic pollutants, especially during periods of very high particulate matter concentrations. PAH concentrations determined by a standard addition method on the filter surface allowed quantification of these harmful substances. However, we were able to show that this method is only suitable for higher-molecular-weight PAHs with low vapour pressure, as measurement artifacts occur due to the application of the solutions and delayed measurements. Nevertheless, the total number of assigned sum formulae and especially of assigned CHO and PAH species was higher in Hangzhou than in Tehran.

As can be seen from our data, there are realistic concerns about a major influence of air pollution in both countries on the environment as well as on the health of the population [90]. The improvement of efficiency in the energy-producing sector and the use of modern filter systems are essential points that can contribute to the reduction of the quantity and danger of harmful particles. China is leading the way in this respect with its restrictive measures, resulting in a reduction of atmospheric pollutants. Our results show a reduced number of sulphates, nitrogen oxides, heavy metals and PAHs in the measured particles from China, while in Iran, despite a significantly lower quantity of particles during our sampling period, the risk potential can be estimated to be much higher due to stronger pollution by heavy metals, PAHs and higher-oxidized nitrogen-containing compounds.

The presented method offers the possibility to quickly and easily access a variety of organic or inorganic molecular information in aerosol particles collected on filter samples. Reliable source apportionment enables the identification of emitters of certain particle populations and can be used as a tool to reduce the emission of selected pollutants.

## Supplementary Information

Below is the link to the electronic supplementary material.

Supplementary file1 (PDF 3060 KB)
