# Supplementary material for: Particle characterization and quantification of organic and inorganic compounds from Chinese and Iranian aerosol filter samples using scanning laser desorption/ionization mass spectrometry
Source: Anal Bioanal Chem. 2022 Sep 1;414(24):7223–41. doi: 10.1007/s00216-022-04275-1 (PMC9482912; doi:10.1007/s00216-022-04275-1)
Supplement: Supplementary file 1 — Supplementary file1 (PDF 3060 KB) [file 216_2022_4275_MOESM1_ESM.pdf]

Particle characterization and quantification of organic and inorganic compounds from Chinese and Iranian aerosol filter samples using scanning laser desorption/ionization mass spectrometry

Christof Barth, Klaus-Peter Hinz, Bernhard Spengler*

Institute of Inorganic and Analytical Chemistry. Justus Liebig University Giessen. 35392 Giessen. Germany

*Corresponding Author: eMail: Bernhard.spengler@anorg.chemie.uni-giessen.de

Supporting Information

1 Microscopic images and height information

Figure 1 a) shows a microscope image of filter sample 287 from Tehran. The left side of the filter was already measured and one can clearly see the homogeneous removal of aerosol particles from the filter surface compared to the right side of the filter. On the right side the fibre structure of the filter is visible and we could even remove material that penetrated deeper into the filter structure. The height information (topography) of the sample is shown in figure 1 b). The total height difference over the entire measured area (slightly brighter areas) was about 400 µm. Without the use of an autofocusing laser system, most of the measurement area would have been out of focus.

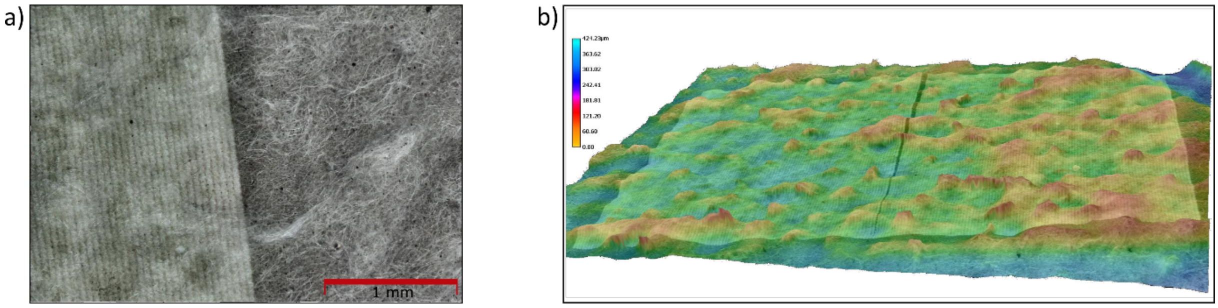

Figure 1: (a) Microscopic image of the measured (left) and unmeasured (right) structure of the filter sample 287 from Tehran and topography (b) of the complete ablated area.

2 Calibration curves for low-molecular-weight PAHs

Figure 2 shows the standard-addition calibration curves for low-molecular-weight PAH species. These curves show very poor correlations, especially for PAHs with high vapor pressure (mass 152.06 and 166.08). For species with mass 178.08 and 202.08, the correlation coefficients are comparable to the lowly volatile PAHs with masses 228.09 to 278.11. However, since a large proportion of the compounds were evaporated into the gas phase, the calculated final concentrations were considered incorrect.

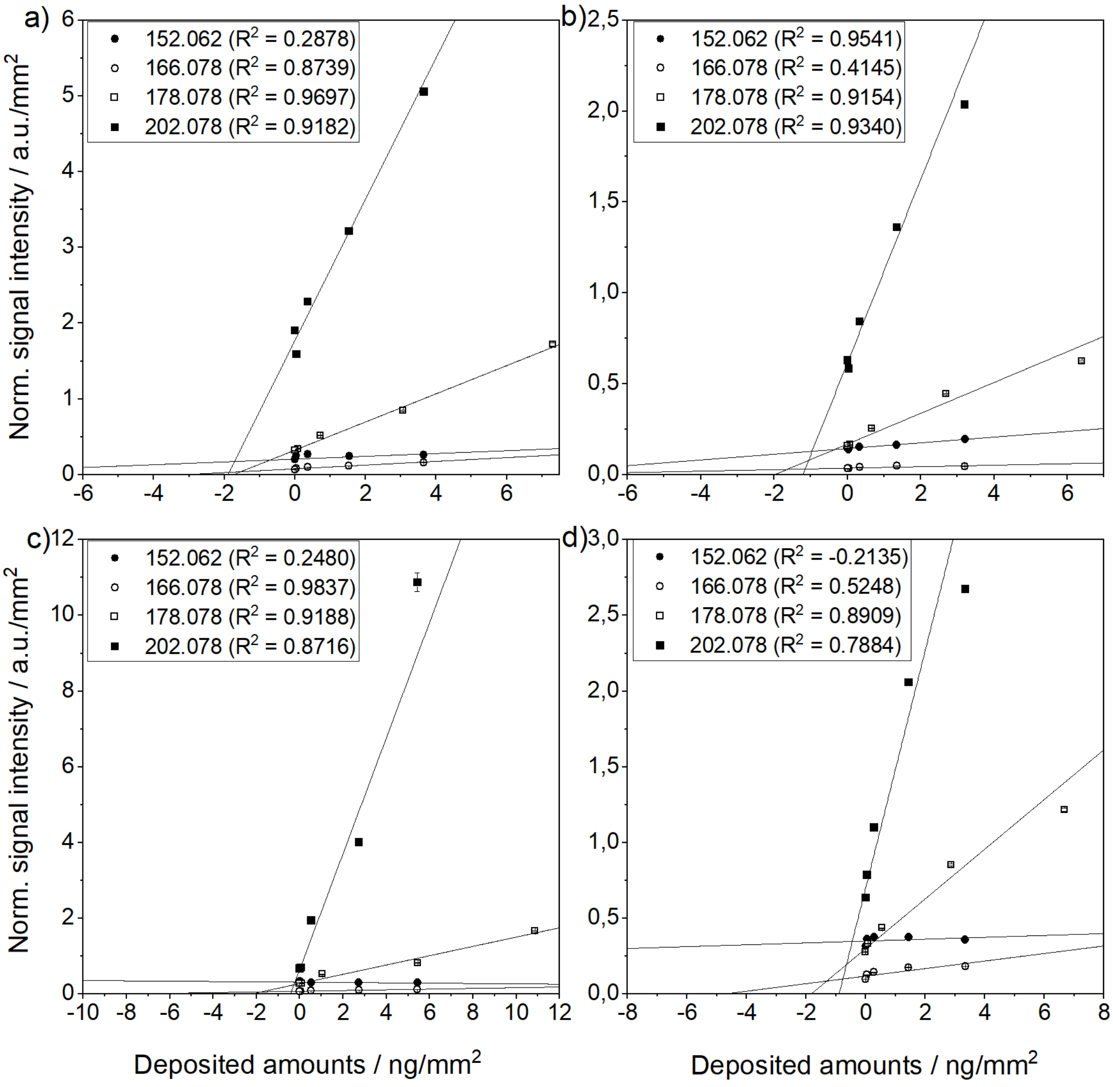

Figure 2: Calibration curves for dried-droplet standard-addition method on Hangzhou samples FPI17B0807 (a), FPI17B0811 (b) and Tehran samples 287 (c) and 289 (d). Calibration curves are shown for low-molecular-weight PAHs.

3 Mass lists with assigned sum formulas for each measurement

The following tables contain all assigned sum formulas in positive- (Table 1) and negative-ion mode (Table 2). If a respective signal was found for a sample, the mass deviation is given in the table. Typical mass error was below ± 5 ppm with a few exceptions which were H_4O_2Ni, H_2OBaCl, O_2N_3Sc and O_3N_3Sc for the positively charged ions. The first two could be easily assigned via isotopic patterns, while the scandium compounds were assigned less reliably. For the negatively charged ions $C_4H_5O_4$, $H_3O_5Ca_2$ and $C_2HO_8FeS_2$, mass deviations were above ±5 ppm.

Table 1: Mass list for all assigned sum formulas in positive-ion mode.

Theoretical mass M^+	Assigned sum formula	Mass error for each filter / ppm			
		FPI17B0807	FPI17B0811	287	289
56.96478	HOCa	-1.282	-1.371	-1.001	-1.103
58.06513	C3H8N				-0.923
58.96045	CaF		-0.651		

59.04914	C3H7O	-1.254	-1.245	-0.833	-0.945
60.04439	C2H6ON	-1.239		-0.811	
60.08078	C3H10N				-0.856
61.03964	CH5ON2	-1.156	-0.855	-0.737	-0.761
62.92905	Cu			-0.759	
62.98173	HONa2	-0.661	-0.807	-0.452	
67.05423	C5H7	-0.567		-0.365	
69.04472	C3H5N2	-0.433		-0.204	
69.06988	C5H9			-0.346	
70.06513	C4H8N	-0.359		-0.187	
71.04914	C4H7O			-0.243	
71.94024	HOMn			-0.208	
71.94822	OSi2	0.402			
71.95187	O2Ca	-0.376	-0.315	-0.221	-0.243
72.04439	C3H6ON	-0.372		-0.170	
72.08078	C4H10N		-0.141	-0.069	-0.066
72.93713	HOFe	-0.087	-0.146	-0.054	-0.023
73.06479	C4H9O	-0.301		-0.151	
74.06004	C3H8ON	-0.111	-0.014	-0.093	-0.042
74.09643	C4H12N	-0.055	0.032	-0.008	-0.015
74.93090	CaCl	-0.050	0.036	-0.021	0.029
74.97535	H3O2Ca	0.045	0.029	-0.030	0.000
77.00837	H5O3Mg	0.014	-0.079	0.098	
77.96882	O2Na2	0.151	0.124	0.136	0.081
78.95567	HOKNa	0.197	0.147	0.163	0.156
78.99704	H4O3Al	-0.157		-0.783	
79.02121	C2H7OS	-0.092		0.067	0.152
79.05423	C6H7	0.111		0.134	
79.95560	H3NCu	0.254		0.203	0.242
80.04948	C5H6N	0.144	0.347	0.199	0.255
80.93962	H2OCu			0.241	
80.94784	ClNa2	0.297	0.269	0.205	0.259
80.95133	FKNa	0.186	0.220		
81.03349	C5H5O		0.367		0.253
81.06988	C6H9	0.189	0.297	0.166	0.231
83.04914	C5H7O	0.237	0.349	0.195	0.259
83.06037	C4H7N2			0.292	0.336
83.08553	C6H11			0.162	
84.08078	C5H10N		0.407	0.355	0.396
84.91125	Rb	0.231	0.263	0.189	0.232
84.95970	CHO2Ca	0.279	0.410	0.252	0.328
85.06479	C5H9O	0.212		0.225	
85.95495	O2NCa	0.434	0.496		
86.06004	C4H8ON	0.285		0.274	
86.09643	C5H12N	0.391	0.397	0.323	0.413
87.02293	C7H3			0.433	
87.04406	C4H7O2	0.242		0.240	

87.90508	Sr	0.355			
87.97698	CONNa2	0.459		0.304	
88.07569	C4H10ON	0.290		0.301	
88.97812	O2BNa2	0.492			
89.03858	C7H5				0.502
89.07094	C3H9ON2	0.337		0.279	
89.93987	H2O2Fe	0.645	0.616	0.537	0.562
89.95080	H3O2Mn	0.634		0.460	0.463
89.96244	H2O3Ca	0.334	0.474	0.460	0.549
90.09134	C4H12ON	0.326	0.499		0.464
90.94770	H3O2Fe	0.488	0.539	0.403	0.422
91.05423	C7H7	0.512	0.518	0.422	0.468
91.99546	H4O4Mg	0.464	0.446	0.428	
92.04948	C6H6N			0.442	
92.94146	H2OCaCl	0.568	0.601	0.411	0.482
92.97191	H5ONNi				0.513
93.05730	C6H7N	0.483		0.463	
93.06988	C7H9	0.451	0.572	0.389	0.474
93.94276	O2KNa	0.541	0.481	0.410	0.442
93.95593	H4O2Ni	-5.770		-0.089	-0.939
94.06513	C6H8N	0.522	0.547	0.482	0.555
94.92961	HOK2	0.536	0.478	0.417	0.472
95.01893	H7O4Mg	0.421	0.445	0.298	0.365
95.04914	C6H7O	0.527	0.563	0.468	0.545
95.06037	C5H7N2			0.511	0.556
95.08553	C7H11	0.486	0.557	0.408	0.494
96.02358	H7O3NAl	0.562	0.484	0.432	0.462
96.04439	C5H6ON	0.563	0.528	0.506	0.558
96.92178	ClKNa	0.585	0.501	0.443	0.528
96.92527	FK2	0.410	0.460		
96.95729	H2O2CaNa	0.596	0.556		
96.99539	CH5O3S			-1.779	
97.00760	H6O4Al	0.442	0.468	0.346	0.400
97.02841	C5H5O2		0.563		0.448
97.06479	C6H9O	0.402	0.566	0.370	0.464
97.07602	C5H9N2		0.505	0.453	0.511
97.08354	C4H10O2Li	0.536			
97.96616	H5ONCu			0.412	0.497
97.96752	C2H2O2Ca	-4.781			
98.01514	C2H6N2Ca		0.168	0.119	
98.09643	C6H12N			0.461	
98.94190	H3O2Zn			0.366	0.516
98.95018	H4O2Cu			0.407	
98.97535	C2H3O2Ca	0.590	0.596	0.298	0.466
98.98414	C5ONa	0.691	0.789	0.591	0.771
99.04406	C5H7O2	0.409	0.532	0.353	0.437
99.08044	C6H11O			0.325	

100.07569	C5H10ON	0.402		0.352	0.449
100.11208	C6H14N	0.592		0.444	0.468
101.05971	C5H9O2	0.413	0.518	0.337	0.402
101.94986	O3NCa	0.589	0.559	0.355	0.458
102.09134	C5H12ON	0.365		0.305	
102.92575	C2CaK	-0.549	-0.589		
102.94818	H3O3Cr			0.350	
102.97026	CH3O3Ca	0.461	0.522	0.400	0.462
103.05423	C8H7		0.520	0.483	0.512
103.92994	CNK2		0.432		
103.95092	CONKNa	0.479	0.424		
103.96551	H2O3NCa	0.537	0.531		
104.04948	C7H6N			0.515	
104.10699	C5H14ON	0.586		0.537	0.485
104.90782	HOSr	0.458	0.401	0.337	0.363
104.93789	H2O3Mn			0.434	
104.95206	O2BKNa	0.420	0.290		
105.00328	CH5O4Mg	0.468	0.437	0.419	
105.03349	C7H5O	0.373			
105.04472	C6H5N2			0.479	
105.06988	C8H9	0.497	0.491	0.442	0.477
105.93479	H2O3Fe	0.512		0.465	
106.05255	C6H6N2			0.528	
106.06513	C7H8N	0.466	0.422	0.472	0.502
106.90455	Ag				0.555
106.96642	H5O2NFe	0.415			
106.98869	H2O3BNa2	0.527			
107.06037	C6H7N2			0.455	
107.07295	C7H9N	0.341		0.353	
107.08553	C8H11	0.397	0.459	0.354	0.415
107.95044	H4O3Fe	0.559	0.482	0.506	0.479
107.96137	H5O3Mn			0.451	
107.96681	O3NNa2	0.596	0.363	0.497	0.456
107.97300	H4O4Ca	0.420	0.410	0.405	0.531
108.04439	C6H6ON			0.400	
108.06820	C6H8N2			0.373	
108.08078	C7H10N	0.402	0.391	0.393	0.437
108.95826	H5O3Fe	0.489	0.414	0.415	0.412
109.02841	C6H5O2				0.394
109.05222	C6H7ON			0.319	
109.06479	C7H9O	0.343	0.441	0.338	
109.07602	C6H9N2		0.341	0.421	0.432
109.10118	C8H13	0.343	0.398	0.294	0.362
109.91670	O2K2	0.383	0.270	0.294	0.305
110.00602	H6O5Mg	0.329	0.268	0.263	0.298
110.03623	C6H6O2			0.156	0.393
110.06004	C6H8ON	0.420	0.327	0.375	0.403

110.92452	HO2K2		0.240		
110.95203	H4O2CaCl	0.485			
110.97535	C3H3O2Ca		0.343		
111.04406	C6H7O2			0.311	0.354
111.08044	C7H11O	0.306	0.360	0.277	
111.09167	C6H11N2		0.271	0.368	0.382
111.11683	C8H15			0.189	
112.89572	ClK2	0.302	0.175	0.251	0.290
113.05730	C4H10O2Na	0.443			
113.05971	C6H9O2	0.169		0.197	
113.09609	C7H13O			0.182	
113.10732	C6H13N2		0.226	0.332	0.331
114.09134	C6H12ON	0.216	0.192	0.190	0.255
115.00665	C3H7O2Ca	0.400			
115.05423	C9H7	0.382	0.192	0.318	0.320
115.07536	C6H11O2	0.157	0.159	0.137	0.184
116.93253	O2KNa2		0.032		
116.95247	H5O3Zn				0.213
116.96618	H5O4Ti	0.321	0.060	0.198	0.198
116.98591	C2H5O3Ca	0.175	0.129	0.174	0.209
117.05730	C8H7N	0.201	0.083	0.254	0.247
117.06988	C9H9		0.135	0.195	0.203
118.06513	C8H8N	0.167	0.067	0.210	0.208
118.08626	C5H12O2N				0.209
118.94310	H3O4Cr			-0.101	-0.491
118.95551	O2N3Sc				-9.374
119.00156	C2H7O3Ca	0.191			
119.01893	C2H7O4Mg	0.182	0.039	0.171	
119.06037	C7H7N2		0.008	0.182	0.171
119.08553	C9H11	0.115	0.052	0.099	
119.89491	O2Sr	0.081	-0.061	-0.013	-0.009
119.92486	CONK2	0.131	-0.068	-0.003	0.052
119.95092	H4O4Cr	0.156	0.051	0.098	0.089
119.96043	H2O4NCa	0.197	0.026	0.074	0.120
120.04439	C7H6ON			0.120	
120.08078	C8H10N	0.122	-0.024	0.138	0.137
120.92602	H4NCaCu	-0.062	-0.217	-0.071	-0.090
120.93632	C2H2OCaK	-0.855	-0.911		
120.96612	H3O4NaP			0.658	
120.98083	CH5O4Ca	0.029	-0.048	0.023	0.050
121.00760	C2H6O4Al			-0.637	
121.02841	C7H5O2		-0.036	0.000	0.038
121.06479	C8H9O			0.043	
121.07602	C7H9N2			0.118	0.101
121.10118	C9H13		-0.007	-0.021	
121.96616	C2H5ONCu	0.552			
121.97608	H4O4NCa	0.077	-0.016	0.044	0.064

122.01510	C10H2		-0.039		
122.04746	C6H6ON2			-0.141	
122.06004	C7H8ON	0.011		0.048	
122.09643	C8H12N	0.003	-0.099	0.009	0.028
122.92452	CHO2K2		-0.372	-0.140	
122.94846	H4O4Mn			0.040	0.037
123.01385	CH7O5Mg	-0.089	-0.228	-0.104	-0.110
123.02293	C10H3		1.529		
123.02325	C2H8O4Al	-0.130		-1.062	
123.04165	C5H8O2Na	0.000	-0.145		
123.05529	C6H7ON2			0.020	
123.08044	C8H11O			-0.036	
123.09167	C7H11N2		-0.158	0.030	
123.11683	C9H15	-0.089		-0.099	
123.91977	O2NK2	-0.027	-0.203	0.051	
123.94075	O3NKNa	-0.011	-0.476	0.068	-0.072
123.94535	H4O4Fe	-0.107	-0.153	-0.085	-0.056
124.07569	C7H10ON			-0.047	
124.11208	C8H14N			-0.030	
124.95318	H5O4Fe		-0.227	-0.040	-0.068
124.96411	H6O4Mn			0.104	
124.97698	H7O3NFe	-0.078	-0.137	-0.055	-0.069
125.04713	C6H7O2N			-0.036	
125.05971	C7H9O2			-0.148	
125.09609	C8H13O	-0.191	-0.170	-0.162	
125.10732	C7H13N2	-0.063	-0.257	-0.058	-0.075
125.96100	H6O4Fe	-0.098	-0.245	-0.116	-0.150
125.98357	H6O5Ca	-0.217	-0.267	-0.408	-0.342
126.92437	H4O2ClFe			-0.161	
126.96883	H7O4Fe			-0.301	
127.03897	C6H7O3				-0.753
127.07267	C3H7N6		-0.304	-0.154	
127.07295	C5H12O2Na	-2.156			-2.366
127.07536	C7H11O2	-0.283		-0.335	
127.11174	C8H15O	-0.315		-0.326	
127.12297	C7H15N2		-0.278	-0.160	
128.06205	C10H8	-0.138	-0.324	-0.167	-0.174
128.91720	HO3Ca2	-0.135	-0.336		-0.215
128.98591	C3H5O3Ca	-0.143	-0.348		
129.03124	C4H10O2K	-0.074			
129.05222	C4H10O3Na	-0.213	-0.390	-0.203	-0.247
129.05462	C6H9O3			-0.482	
129.06988	C10H9		-0.351	-0.257	-0.246
129.09101	C7H13O2	-0.345	-0.365	-0.364	-0.328
129.99374	C3H6O3Ca	-0.203	-0.613		
130.06513	C9H8N	-0.241	-0.447	-0.215	-0.211
130.15903	C8H20N		-0.496		

130.93285	H3O3Ca2	-0.208	-0.421	-0.234	-0.258
130.97898	C2HO3Be2Ca	-0.254			
131.00156	C3H7O3Ca	-0.254	-0.448		-0.303
131.04914	C9H7O				-0.278
131.06037	C8H7N2			-0.274	
131.08553	C10H11		-0.443	-0.357	-0.329
131.95327	H4O5Ti	-0.328	-0.607	-0.360	-0.354
132.06820	C8H8N2			-0.310	
132.08078	C9H10N	-0.329	-0.531	-0.309	-0.309
132.10191	C6H14O2N				-0.312
132.90490	Cs	-0.333	-0.651	-0.329	-0.355
132.92852	H2O2Ca2F	-0.153			
132.96110	H5O5Ti			-0.485	
132.96587	H5O4CaMg	-0.289	-0.505		
133.00760	C3H6O4Al			-0.852	
133.01721	C3H9O3Ca	-0.259	-0.532		
133.06479	C9H9O			-0.398	
133.07602	C8H9N2		-0.589	-0.345	-0.352
133.10118	C10H13	-0.432	-0.520	-0.464	
133.98865	C2H6O4Ca	-0.370	-0.539		
134.06004	C8H8ON			-0.404	
134.07127	C7H8N3			-0.351	-0.295
134.09643	C9H12N			-0.432	
134.95043	O3N3Sc				-8.716
134.99648	C2H7O4Ca	-0.429	-0.619	-0.471	-0.418
135.00286	C3H5O3Na2	-0.814	-0.655	-0.464	
135.01288	C3H8O2CaF	-0.459			
135.02293	C11H3	1.423		-0.226	
135.03924	C4H9O2Na2	-0.296			
135.04406	C8H7O2			-0.514	
135.05023	C3H11O4Mg	-0.466			
135.05964	C4H12O3Al	-0.459			
135.08044	C9H11O		-0.599	-0.505	
135.09167	C8H11N2				-0.442
135.11683	C10H15	-0.504	-0.597	-0.563	
135.94535	CH4O4Fe	-0.531			-0.497
135.95628	CH5O4Mn			-0.478	
136.02167	C2H8O5Mg	-1.369	-0.520	-0.572	-1.428
136.03931	C7H6O2N				-0.477
136.05488	C3H11O3NAl	-0.495			
136.06177	C5H6N5		-0.665		
136.07328	C6H11ONNa	-0.480		-0.452	
136.07569	C8H10ON	-0.524	-0.687	-0.577	
136.11208	C9H14N	-0.502		-0.561	
136.90439	HOSn	-0.420	-0.599	-0.392	
136.94019	O4AlNa2		-0.875	-0.697	
136.95318	CH5O4Fe	-0.494		-0.536	-0.527

137.02332	C7H5O3			-0.110	-0.782
137.02950	C2H9O5Mg	-0.596	-0.775	-0.620	-0.598
137.03890	C3H10O4Al	-0.494	-0.913	-1.226	-1.021
137.05730	C6H10O2Na	-0.531	-0.723	-0.525	
137.05971	C8H9O2			-0.627	
137.07094	C7H9ON2			-0.524	
137.09609	C9H13O	-0.633		-0.625	
137.10732	C8H13N2		-0.749	-0.545	
137.97099	H4O5NCa	-0.494	-0.748	-0.548	-0.521
138.03415	C2H9O4NAl	-0.654	-0.839	-1.075	
138.09134	C8H12ON			-0.595	
138.12773	C9H16N			-0.557	-0.523
138.93657	H2O3BK2		-0.821		
138.93658	H6ONCaCu			-0.714	
138.99139	CH7O5Ca	-0.682	-0.789	-0.792	-0.704
139.01817	C2H8O5Al	-0.646	-0.853	-0.639	-0.643
139.02940	CH8O4N2Al			-0.718	
139.03657	C5H8O3Na	-0.617	-0.809	-0.596	
139.05020	C6H7O2N2			-0.681	
139.05423	C11H7	-0.538	-0.750	-0.545	-0.548
139.07267	C4H7N6			0.333	
139.07536	C8H11O2			-0.716	
139.11174	C9H15O	-0.718		-0.708	
139.12297	C8H15N2		-0.833	-0.621	-0.617
139.91469	O3NK2	-0.609	-0.936	-0.557	-0.595
139.98664	H6O5NCa		-0.818	-0.775	
140.00401	H6O6NMg		-0.853	-0.660	-0.684
140.95902	H6O5Mn	-0.390	-0.386	-0.087	-0.334
140.97149	H4O4AlNa2	-0.689		-0.746	
141.06988	C11H9	-0.647	-0.885	-0.665	-0.663
141.08832	C4H9N6			0.364	0.267
141.09101	C8H13O2	-0.831		-0.834	
141.13862	C8H17N2		-0.917		
142.07770	C11H10		-0.413	-0.298	-0.275
142.93855	HO4Na2S			-0.263	-0.328
142.94811	C5H3Ca2	-0.620		-0.655	
143.00156	C4H7O3Ca	-0.285	-0.476		
143.07027	C7H11O3			-0.434	
143.07295	C10H9N		-0.494	-0.357	
143.08553	C11H11		-0.479	-0.350	-0.333
143.10666	C8H15O2	-0.426	-0.464	-0.433	-0.387
143.92711	H5O3ClFe			-0.358	
144.04439	C9H6ON			-0.312	
144.08078	C10H10N	-0.381	-0.546	-0.346	-0.335
144.10191	C7H14O2N			-0.394	
144.92744	CO3KNa2		-0.612		
144.98083	C3H5O4Ca	-0.370			

145.01721	C4H9O3Ca	-0.405			
145.02615	C4H10O3K			-0.475	
145.06479	C10H9O		-0.551	-0.384	-0.380
145.07602	C9H9N2			-0.384	-0.374
145.10118	C11H13		-0.521	-0.445	
145.91994	H2O4Ca2	-0.450	-0.664		
146.01510	C12H2		-0.598		
146.06004	C9H8ON			-0.422	
146.09643	C10H12N			-0.441	
146.88332	O2Ca2Cl	-0.393			
146.91816	O5AlCa	1.016			
146.92777	H3O4Ca2	-0.407	-0.593		
146.99648	C3H7O4Ca	-0.421	-0.624	-0.475	-0.484
147.02293	C12H3		-0.174		
147.03286	C4H11O3Ca	-0.252			
147.04406	C9H7O2		-0.636	-0.487	-0.471
147.08044	C10H11O			-1.098	
147.09167	C9H11N2		-0.701	-0.418	-0.444
148.00430	C3H8O4Ca	-0.427	-0.716	-0.533	-0.435
148.07569	C9H10ON		-0.700	-0.503	
148.08692	C8H10N3			-0.456	-0.494
148.11208	C10H14N			-0.529	-0.511
148.89897	H2O2Ca2Cl	-0.089	-0.181		
148.94342	H5O4Ca2	-0.042	-0.207	-0.142	-0.122
148.97698	C2H7O3NFe	-0.177			-0.041
148.98956	C3H9O3Fe	-0.057	-0.171	-0.087	-0.064
149.01213	C3H9O4Ca	-0.104	-0.258	-0.328	-0.157
149.02332	C8H5O3	-0.245	-0.230	-0.193	-0.149
149.05971	C9H9O2			-0.186	
149.09609	C10H13O		-0.194		
149.96100	C2H6O4Fe	-0.165		-0.135	-0.133
149.97193	C2H7O4Mn			-0.081	
150.03386	C5H8N3Ca		-0.352	-0.339	
150.03732	C3H10O5Mg	-0.212	-0.378		-0.227
150.09134	C9H12ON			-0.184	
150.91944	C2HO3K2		-0.317		
150.93908	H4O3Ca2F	0.046			
150.96883	C2H7O4Fe	-0.173	-0.289	-0.188	-0.183
150.97680	C3H5O3KNa	-0.465	-0.382	-0.260	
150.98333	C3H8O2CaCl	-0.167			
151.01817	C3H8O5Al	-0.406	-1.294	-0.623	
151.02778	C3H11O4Ca	-0.320			
151.05455	C4H12O4Al	-0.168			
151.07295	C7H12O2Na	-0.234	-0.331	-0.192	
151.08659	C8H11ON2			-0.211	
151.09649	C6H15O4	-0.387		-0.535	
151.11174	C10H15O	-0.327	-0.290	-0.310	-0.261

151.12297	C9H15N2		-0.343		
151.97665	C2H8O4Fe			-0.221	-0.221
151.99922	C2H8O5Ca	-0.314	-0.330		
152.06205	C12H8	-0.223	-0.334	-0.186	-0.187
152.95267	H5O5CaSi	-0.275			-0.332
152.96511	H6O5AlCa	-0.230	-0.366		-0.058
152.97190	CH7O4NFe			-0.228	-0.261
153.00704	C2H9O5Ca	-0.302	-0.443	-0.410	-0.358
153.01824	C7H5O4				-0.268
153.03382	C3H10O5Al			-1.181	
153.05222	C6H10O3Na	-0.342	-0.408	-0.298	
153.06988	C12H9		-0.375	-0.258	-0.275
153.09101	C9H13O2			-0.369	
153.12739	C10H17O	-0.435		-0.394	
153.13862	C9H17N2		-0.444	-0.302	
153.95592	CH6O5Fe	-0.388	-0.382	-0.307	-0.288
154.02606	C7H6O4	1.481		0.487	0.015
154.04987	C7H8O3N				-0.304
154.06513	C11H8N	-0.312		-0.285	
154.07770	C12H10				-0.287
154.90050	H3OAl3Mn	1.096			
154.90743	HOBa	-0.234	-0.393	-0.193	-0.202
154.96832	H7O5CaSi			-0.359	
154.99813	H8O6AlMg	-0.158			
155.03148	C5H8O4Na			-0.306	
155.03389	C7H7O4				-0.337
155.08553	C12H11		-0.457	-0.331	-0.353
155.10425	C7H16O2Na	-0.340	-0.514		
155.10666	C9H15O2	-0.514		-0.465	
155.86479	O2KRb	-0.564			
155.92810	CH2O3NCa2	-0.379			
155.98156	H6O6NCa	-0.360	-0.547	-0.405	-0.392
156.02058	C10H4O2		-0.481	-0.359	
156.08078	C11H10N	0.010	-0.044	0.053	
156.84520	BrK2		-0.082		
156.90309	BaF	-0.011	0.002		
156.91212	CHO4Ca2	-0.457			
156.93312	HON2AlKNa2		-0.577		
156.93323	CH5O2NKMn	-1.535		-1.203	
156.94543	H4O4AlKNa	-0.056	-0.130	-0.011	
156.98679	H7O6Al2	-0.006	-0.040	0.174	0.016
156.99748	C3H9O4Ti	0.038		1.601	
157.00728	C13H		-0.020		-0.020
157.01721	C5H9O3Ca	0.019	-0.070		
157.07602	C10H9N2		-0.094	0.081	
157.08352	C6H14O3Na	-0.078	-0.113	0.055	-0.072
157.12231	C9H17O2	-0.187		-0.109	

157.88368	O3K2S	-0.105			
158.09643	C11H12N	-0.070	-0.150	-0.037	-0.030
158.15394	C9H20ON	-0.160		-0.124	-0.131
158.91250	H3O3NKMn			-0.152	-0.200
158.92212	C2H2O2K2Na	-0.790	-0.922	-0.793	-0.756
158.92777	CH3O4Ca2	-0.085			
158.97675	C2H7O5Ti			0.309	
158.99001	H8O6AlSi			-0.119	-0.154
158.99648	C4H7O4Ca	-0.112			
159.03286	C5H11O3Ca	-0.163			
159.05023	C5H11O4Mg	-0.031			
159.08044	C11H11O		-0.131	-0.117	
159.09167	C10H11N2		-0.193	-0.054	-0.058
159.11683	C12H15		-0.142		
159.92302	H2O4NCa2	-0.153			
160.05188	C10H8O2				-0.430
160.07569	C10H10ON			-0.096	
160.90138	CO3K2Na		-0.298		
161.01213	C4H9O4Ca	-0.048	-0.239	-0.126	-0.093
161.03188	C5H7O5N			1.601	
161.03858	C13H5	1.597			
161.05971	C10H9O2			-0.150	-0.151
161.07094	C9H9ON2			-0.100	
161.07529	C6H14O3Al	-0.223			
161.09609	C11H13O	-0.242	-0.236	-0.223	-0.183
161.10732	C10H13N2	-0.156	-0.292	0.013	-0.044
162.00738	C3H8O4NCa	-0.110			
162.01995	C4H10O4Ca	-0.067			
162.05496	C9H8O2N			-0.129	
162.09134	C10H12ON			-0.172	
162.10257	C9H12N3			-0.141	
162.94096	H5O5MnNa	0.001			
162.94100	H2O4Na3Si				-0.201
162.97976	C3H8O4Mn	-0.153		-0.098	-0.121
163.00521	C4H11O3Fe	0.055			
163.02778	C4H11O4Ca	-0.215	-0.478	-1.441	-0.385
163.03897	C9H7O3		-0.275	-0.226	-0.216
163.05423	C13H7	0.294	-0.054	-0.097	-0.131
163.07536	C10H11O2			-0.679	
163.08659	C9H11ON2			-0.213	
163.12297	C10H15N2		-0.377	-0.194	-0.202
163.13287	C8H19O3			-0.347	
163.93051	H4O5Ca2	-0.237	-0.340		-0.197
163.99922	C3H8O5Ca	-0.281	-0.320		
164.05297	C4H12O5Mg	-0.215			
164.08184	C8H10ON3			0.246	
164.89388	H2O3Ca2Cl	0.206			

164.92049	O4Na3S	0.169	0.090	0.339	0.239
164.93001	HO4Na3P	-1.978		0.060	
165.00704	C3H9O5Ca	0.216	0.086	0.134	0.418
165.03382	C4H10O5Al			-0.580	
165.04343	C4H13O4Ca	0.245			
165.06988	C13H9	0.172	0.100	0.190	0.177
165.08860	C8H14O2Na	0.111	0.064	0.118	
165.12739	C11H17O			0.046	
165.13862	C10H17N2		0.059		
165.92617	H3O4Ca2F	-4.215			
165.99230	C3H10O4Fe	0.327	0.251	0.145	0.203
166.01487	C3H10O5Ca	0.110	0.017	-0.065	0.048
166.07770	C13H10				0.152
166.08626	C9H12O2N		0.043	0.062	
166.90953	H4O3Ca2Cl	0.086	0.064		0.140
166.95074	C3H5O3K2	-0.041	-0.042	0.047	0.046
166.95398	H7O5Ca2	0.109	0.012		
166.95408	C3H5O2Al2Ca			1.623	2.180
166.98755	C2H9O4NFe	-1.048		0.048	0.048
167.02269	C3H11O5Ca	-0.043	-0.160	-0.981	
167.04689	C7H12O2K		-0.010	0.091	
167.04947	C4H12O5Al			-1.148	
167.06787	C7H12O3Na			0.079	
167.07027	C9H11O3		0.027	0.025	0.066
167.07295	C12H9N	0.076	0.015	0.055	
167.08553	C13H11		0.039		0.075
167.10666	C10H15O2	-0.044	0.022	-0.058	
167.14304	C11H19O			-0.033	
167.15427	C10H19N2		-0.037		
167.97157	C2H8O5Fe	0.004	0.038	0.059	0.097
168.08078	C12H10N	0.049	-0.024	0.043	
168.94965	H6O4Ca2F	-2.679			
168.99389	C3H10O3CaCl	0.042			
169.01721	C6H9O3Ca	-0.035			
169.03562	C5H5O3N4				-2.364
169.06479	C12H9O		-0.034	0.065	0.017
169.07602	C11H9N2		-0.117	0.029	0.018
169.10118	C13H13				0.007
169.12231	C10H17O2	-0.120	-0.062	-0.088	
169.89452	O2Ba	0.047	-0.048	0.090	0.093
170.01510	C14H2		-0.051		
170.06004	C11H8ON			0.022	
170.08385	C11H10N2			0.010	-0.012
170.09643	C12H12N		-0.119	-0.019	
170.85434	Cl2K2Na		-0.170		
170.92536	H4O4Ca2Na	0.020			
170.92777	C2H3O4Ca2	-0.127			

170.94608	C2H2O3Na3Si			0.001	-0.071
170.96324	H7O6CaSi	0.007	-0.056	0.001	-0.025
170.97535	C8H3O2Ca	1.791	1.757	1.341	1.758
171.02640	C5H8O5Na			-0.051	
171.03286	C6H11O3Ca	-0.041			
171.04406	C11H7O2				-0.029
171.07819	C7H16O2K		-0.153		
171.09167	C11H11N2		-0.170	-0.009	-0.029
171.09917	C7H16O3Na	-0.130		-0.102	
171.10157	C9H15O3			-0.266	
171.13796	C10H19O2	-0.219		-0.160	
171.98457	C3H8O5Ti	-0.067	-0.368	-0.094	-0.096
172.00430	C5H8O4Ca	-0.148	-0.205		
172.03931	C10H6O2N			-0.017	
172.05806	C6H12O4Mg	-0.062			
172.07569	C11H10ON			-0.075	
172.09950	C11H12N2			0.007	
172.11208	C12H14N	-0.098		-0.086	
172.13321	C9H18O2N	-0.273			
172.16959	C10H22ON			-0.213	
172.87354	BaCl	0.384	0.384	0.424	0.412
172.90706	HON2AlK2Na	-0.462	0.032	0.083	-0.169
172.91937	H4O4AlK2	0.232	0.183	0.106	-0.068
172.94342	C2H5O4Ca2	0.318	0.235		
172.97759	H2O5B2Na3	0.109			
172.99625	H9O7MgSi	0.323		0.344	0.292
173.01213	C5H9O4Ca	0.305	0.306		
173.04851	C6H13O3Ca	0.304			
173.05745	C6H14O3K		0.197	0.276	0.265
173.05971	C11H9O2				0.231
173.07094	C10H9ON2		0.261		
173.07843	C6H14O4Na	0.229	0.221	0.322	0.229
173.10732	C11H13N2	0.320	0.239	0.311	0.300
173.91037	H2O2Ce	-0.319			
173.93867	CH4O4NCa2	0.313			
173.96384	C2H6O6Ti			0.288	0.219
174.09134	C11H12ON			0.272	
174.88642	HO4K2S	0.251	0.118	0.237	0.212
174.89606	C2H2O2K3	-0.464	-0.528	-0.335	-0.396
174.92268	CH3O5Ca2	0.284			
174.97249	H7O7Si2			0.278	0.275
174.98492	H8O7AlSi	0.248		0.255	0.203
174.99736	H9O7Al2			2.547	
175.01784	C13H3O		0.435		
175.02778	C5H11O4Ca	0.327	0.184	0.199	0.218
175.03897	C10H7O3				0.238
175.05770	C5H12O5Na	-0.530			1.720

175.07536	C11H11O2			0.199	
175.08659	C10H11ON2		0.157	0.245	
175.09094	C7H16O3Al	0.103			
175.12297	C11H15N2		0.135	0.246	
175.97665	C4H8O4Fe	0.260			
175.99922	C4H8O5Ca		0.194		
176.06205	C14H8	0.184	0.121	0.258	0.224
176.07061	C10H10O2N			0.207	0.187
176.87532	CO3K3	0.092	0.060		
176.93833	CH5O5Ca2	0.164	0.237		
176.98448	C4H9O4Fe	0.248			
176.99245	C5H7O3KNa		0.074		
177.00704	C4H9O5Ca	0.157	0.125	0.111	
177.03382	C5H10O5Al			-0.436	
177.04343	C5H13O4Ca	0.179	-0.055		
177.05462	C10H9O3			0.163	
177.06080	C5H13O5Mg	0.150			
177.06988	C14H9	1.669			0.338
177.08860	C9H14O2Na	0.121	0.065	0.180	0.185
177.09101	C11H13O2			-0.080	
177.10224	C10H13ON2			0.163	
177.12739	C12H17O	0.019		0.085	
177.13862	C11H17N2			0.186	
177.16378	C13H21	0.018	0.112	0.074	0.148
177.93358	H4O5NCa2	0.147			
178.00229	C3H8O5NCa	0.224	0.056		0.258
178.01487	C4H10O5Ca	0.072	0.102		
178.07770	C14H10	0.122	0.064	0.181	0.150
178.08626	C10H12O2N			0.165	
178.09749	C9H12ON3			0.137	
179.02269	C4H11O5Ca	-0.134	-0.078	-1.907	
179.04689	C8H12O2K			0.120	
179.04947	C5H12O5Al	-0.085		-1.114	
179.06787	C8H12O3Na		0.012	0.087	
179.08553	C14H11	0.300	0.041		0.132
179.10425	C9H16O2Na	0.054		0.110	
179.10666	C11H15O2			-0.069	
179.15427	C11H19N2		-0.014		
179.90286	H4O5CaFe	0.008			
179.97157	C3H8O5Fe	0.118		0.121	0.100
180.00795	C4H12O4Fe	0.439			
180.01794	C3H10O5NCa	0.106			
180.03052	C4H12O5Ca	0.144			
180.08078	C13H10N	0.060	-0.027	0.089	0.082
180.09335	C14H12				0.094
180.88811	H5O4Fe2	0.091		0.336	0.152
180.89443	O4KNa2S	0.003	-0.104	0.248	0.108

180.90378	C2H2CuNa4	0.915	0.825	0.928	
181.00728	C15H		0.009	0.089	0.043
181.02615	C7H10O3K		-0.134		
181.02873	C4H10O6Al	-0.321		-0.563	
181.06254	C8H14O2K	-0.034	-0.095	-0.109	
181.06479	C13H9O		-0.045		
181.06512	C5H14O5Al			-1.799	-1.804
181.07602	C12H9N2			0.084	
181.08352	C8H14O3Na	-0.079	-0.061	0.029	
181.08860	C13H11N	0.026		0.018	
181.10118	C14H13		-0.033		0.020
181.12231	C11H17O2			-0.114	
181.16992	C11H21N2		-0.104		
181.91851	H6O5CaFe	-0.031	0.138	0.039	0.012
181.94107	H6O6Ca2	-0.108	-0.120		
182.01510	C15H2		-0.102		
182.08385	C12H10N2	0.487		0.459	
182.09643	C13H12N			0.431	
182.19033	C12H24N		0.363		
182.90234	CHO2Ba	0.396	0.436	0.455	0.474
182.92633	H7O5CaFe	0.384	0.469		
183.00954	C4H12O3CaCl	0.453			
183.01761	C3H11O6Ca	0.316	0.372	0.347	0.319
183.02293	C15H3		0.525		
183.04180	C7H12O3K		0.296		
183.08044	C13H11O	0.277	0.351	0.310	0.311
183.09167	C12H11N2	0.347	0.313	0.375	0.364
183.10157	C10H15O3			0.244	
183.11683	C14H15		0.385		
183.13796	C11H19O2			0.294	
183.89759	O2NBa		-2.081		-0.179
184.07328	C10H11ONNa			0.287	0.184
184.07569	C12H10ON			0.379	
184.09950	C12H12N2			0.439	0.400
184.92010	H6O4Ca2Cl	0.276	0.309	0.166	0.220
185.00219	C14HO		0.370		
185.01213	C6H9O4Ca	0.328	0.262		
185.02107	C6H10O4K		0.213		
185.02600	C14H3N		0.365	0.415	0.371
185.04205	C6H10O5Na	0.278	0.268	0.480	0.329
185.07094	C11H9ON2			0.345	
185.08352	C12H11ON			0.167	
185.10732	C12H13N2		0.290	0.357	
185.11482	C8H18O3Na	0.271		0.313	
185.11722	C10H17O3			-0.043	
185.15361	C11H21O2	0.157		0.222	
185.85418	HO3ClCuK			1.865	

185.89883	H4O4FeKNa		0.202		
186.00022	C4H10O5Ti	0.454			
186.09134	C12H12ON			0.296	
186.10257	C11H12N3			0.371	
186.11515	C12H14N2			0.387	
186.14886	C10H20O2N	0.128			
186.93558	H7O6FeSi	0.324		0.437	0.354
187.02564	C5H11ONCaNa2	-0.769			
187.02778	C6H11O4Ca	0.257	0.271		
187.03657	C9H8O3Na		0.795		
187.03897	C11H7O3				0.290
187.05423	C15H7	0.546	0.394	0.364	0.328
187.07310	C7H16O3K		-0.028	0.059	
187.08659	C11H11ON2			0.278	
187.12297	C12H15N2		0.203	0.257	
187.90508	H2O3Ba			0.307	0.356
187.93051	C2H4O5Ca2	-0.238			
188.02567	C14H4O		0.272		
188.03560	C6H12O4Ca	0.345	0.086		
188.06835	C6H15O3NK		-0.057		
188.90198	HO2N2AlK2Na	-0.529			
188.93833	C2H5O5Ca2	0.354			
188.97380	H9O7CaSi	0.221		0.274	0.231
189.00704	C5H9O5Ca	0.183	0.193		
189.04343	C6H13O4Ca	0.336	0.194		
189.05237	C6H14O4K	0.024	0.051	0.164	0.151
189.05462	C11H9O3		-0.028		0.072
189.06988	C15H9	0.394	0.189	0.301	0.283
189.07981	C7H17O3Ca		-0.123		
189.10224	C11H13ON2			0.201	
189.13862	C12H17N2		0.127	0.202	0.192
189.99230	C5H10O4Fe	0.213			
189.99236	C12O2N				-0.038
190.01487	C5H10O5Ca	0.107	0.105		
190.04987	C10H8O3N			0.315	
190.05125	C6H14O4Ca	-0.441			
190.06862	C6H14O5Mg	0.053			
190.07770	C15H10				0.204
190.08626	C11H12O2N			0.194	0.172
190.88411	H2OBaCl		7.114	3.685	
190.93425	H7O6CaTi	0.263			
190.95398	C2H7O5Ca2	0.169	0.106	-2.285	-2.590
191.00013	C5H11O4Fe	0.408			0.204
191.01463	C6H12O2CaCl	-0.084			
191.02269	C5H11O5Ca	0.078	0.107	-0.169	0.131
191.03163	C5H12O5K	-0.200	-0.181		-0.359
191.03389	C10H7O4				0.241

191.05908	C6H15O4Ca	0.161	-0.018	-3.613	
191.07645	C6H15O5Mg	0.150			
191.08553	C15H11	0.903	0.124	0.224	0.186
191.10425	C10H16O2Na	0.097	0.019	0.214	0.146
191.92542	CH4O6Ca2	0.130			
191.97157	C4H8O5Fe	0.071		0.237	0.160
191.98194	C7H5O4K				2.747
191.99413	C4H8O6Ca	0.258			
192.01794	C4H10O5NCa	0.070			
192.06552	C10H10O3N			0.191	
192.09335	C15H12	0.126	0.073		0.131
192.10191	C11H14O2N			0.160	
192.86211	C3HK4		3.078		
192.96639	C5H7O3K2		0.466		
193.00196	C4H9O6Ca		0.612		
193.03834	C5H13O5Ca	0.564	0.415		
193.04954	C10H9O4			0.598	
193.06254	C9H14O2K	0.419	0.452	0.235	0.142
193.06512	C6H14O5Al	-0.265			
193.07335	C10H11O3N			1.271	
193.08352	C9H14O3Na	0.398	0.447	0.624	0.496
193.08592	C11H13O3			-0.034	
193.10118	C15H13		0.769	0.552	0.603
193.11990	C10H18O2Na	0.423		0.505	
193.15869	C13H21O			0.407	
193.98722	C4H10O5Fe	0.509		0.552	0.502
194.01510	C16H2	0.426	0.451		
194.03359	C4H12O5NCa	0.502			
194.08385	C13H10N2			0.615	
194.09643	C14H12N	0.475	0.457	0.460	0.466
194.10900	C15H14	0.521			0.483
194.88901	ON2AlK2Na2		-0.347		
194.90445	CH4O4Ca2Cl	0.403			
194.94299	H11O5FeTi	3.136		3.097	3.113
194.94890	CH7O6Ca2	0.340	0.418		
195.04180	C8H12O3K		0.373	0.400	
195.05399	C5H15O5Ca		0.317		
195.06278	C8H12O4Na		0.425		
195.07819	C9H16O2K		0.410	0.369	
195.08044	C14H11O		0.476		0.479
195.09167	C13H11N2		0.405	0.462	
195.09917	C9H16O3Na		0.410	0.503	
195.10425	C14H13N	0.424		0.426	0.434
195.11683	C15H15				0.435
195.12270	C8H19O5	0.239		0.287	
195.17434	C13H23O	0.248	0.431	0.349	
195.94415	H6O6NCa2		0.391		

196.01286	C3H10O6NCa	0.426	0.367	0.620	0.512
196.84739	O3K3S	0.415			
196.86837	O4K2NaS	0.313	0.283	0.493	0.413
196.87800	C2HO2K3Na	-0.317	-0.301		
196.90396	H5O6Ti2	0.373			0.309
196.91799	C2H3O2Ba	0.383	0.421	0.483	0.455
196.95443	H6O7NaSi2			0.367	0.316
197.01213	C7H9O4Ca	0.350	0.402		
197.02107	C7H10O4K		0.305		
197.04205	C7H10O5Na			0.403	
197.05745	C8H14O3K		0.291	0.235	
197.05971	C13H9O2			0.322	
197.07094	C12H9ON2			0.433	0.391
197.09475	C12H11N3			-1.972	
197.10732	C13H13N2		0.301	0.368	
197.87331	O3NClK2Na		0.237		
197.89085	H6O5Fe2	0.338	0.914	1.085	1.321
197.91342	H6O6CaFe	0.292			
198.04108	C4H14O6Ca	0.158			
198.07876	C12H10ON2			0.389	0.365
198.10257	C12H12N3			0.364	
198.89868	H7O5Fe2			0.369	
198.99139	C6H7O5Ca	0.260			
198.99793	C4H9O5KNa			-0.771	
199.00033	C6H8O5K		0.293		
199.01784	C15H3O		0.439		
199.02778	C7H11O4Ca	0.295	0.254		
199.03672	C7H12O4K		0.168		
199.03897	C12H7O3		0.289		0.306
199.04502	C12H9NS	0.385			
199.07310	C8H16O3K		0.109		
199.08659	C12H11ON2			0.310	0.289
199.11174	C14H15O			0.134	
199.16926	C12H23O2			0.235	
199.89251	O3NBa	0.358	0.301	0.390	0.361
199.92907	H8O6CaFe	0.212	0.247		0.259
200.06205	C16H8	-0.087	0.230	-0.065	-0.067
200.09441	C12H12ON2			-0.044	
200.91291	CH3O3Ba			0.758	0.100
201.00704	C6H9O5Ca		-0.116		
201.01598	C6H10O5K	-0.075	0.098	-0.089	-0.081
201.02011	C4H14O4CaCl	0.109			
201.04343	C7H13O4Ca	0.035	0.140		
201.05462	C12H9O3		0.149		-0.044
201.06988	C16H9				0.045
201.07981	C8H17O3Ca	-0.054			
201.08875	C8H18O3K	-0.293	-0.052	-0.300	-0.392

201.10224	C12H13ON2		0.131	-0.076	
201.13862	C13H17N2		0.068		
201.87277	H4O4FeK2	-0.084	-0.063		
202.03868	C6H12O4NCa	0.017			
202.07770	C16H10	-0.008	-0.024	-0.025	-0.013
202.88527	H3O5Ca3	4.039			
202.99037	C4H11O4Ca2	0.116			
203.01106	C6H12O4Mn	-1.736			
203.02269	C6H11O5Ca	-0.085	-0.095		
203.03163	C6H12O5K		-0.140		
203.03389	C11H7O4				-0.010
203.05261	C6H12O6Na	-0.011	-0.131	-0.019	
203.05770	C11H9O3N			0.651	
203.05908	C7H15O4Ca	-0.031	-0.156		
203.08553	C16H11		0.137		-0.050
203.15427	C13H19N2		0.708		
203.93884	H4O9Si2	-2.526		-2.512	-2.547
203.94925	H9O7MnSi			0.107	
203.96181	C3H8O5Ca2	-0.113			
204.00795	C6H12O4Fe	0.161			
204.02058	C14H4O2		0.682		
204.03052	C6H12O5Ca		0.642		
204.06552	C11H10O3N			0.043	-0.003
204.06690	C7H16O4Ca	0.079	0.046		
204.08078	C15H10N	0.054	0.599	-0.035	
204.09335	C16H12	0.088			0.015
204.10191	C12H14O2N			0.010	
204.90078	H3ON2Ca2Na2S			0.875	
204.90092	H5O5Ca3		0.020	1.287	
204.94667	H5O9Si2			-2.517	-2.547
205.00196	C5H9O6Ca	0.186			
205.00728	C17H		0.762		
205.01578	C6H13O4Fe	1.323			
205.03834	C6H13O5Ca	0.070	0.584		
205.06254	C10H14O2K	-0.701	0.124		
205.06479	C15H9O	0.143	0.587		
205.06826	C6H14O6Na	0.075		0.556	0.926
205.07335	C11H11O3N			1.122	
205.07473	C7H17O4Ca	0.002	0.947		
205.10118	C16H13	1.352			0.069
205.89878	H6O6FeTi	0.546			
205.92134	H6O7CaTi	0.075			
205.94107	C2H6O6Ca2	-0.008			
206.01510	C17H2		0.394		
206.02360	C6H14O4Fe	0.061			0.020
206.04617	C6H14O5Ca	-0.036			
206.08117	C11H12O3N			0.070	0.029

206.09643	C15H12N			-0.002	
206.10900	C16H14	0.071	0.412		0.012
206.11756	C12H16O2N			0.028	
206.89659	H4O4Ca3F	0.419			
206.93394	H7O6Ca2Mg	0.216			
207.01761	C5H11O6Ca		0.323		
207.02293	C17H3		0.356		
207.04180	C9H12O3K		0.196		
207.05399	C6H15O5Ca	0.149			
207.07642	C10H11O3N2			0.260	
207.07819	C10H16O2K	0.028	0.256	0.062	0.000
207.08044	C15H11O				0.058
207.09917	C10H16O3Na	-0.088	0.250	0.038	-0.029
207.11683	C16H15				-0.007
208.00287	C5H12O5Fe	0.134		0.056	0.002
208.09682	C11H14O3N			-0.936	
208.11208	C15H14N	0.044	0.162	-0.003	0.009
208.12465	C16H16	0.044			
208.91834	O6N2KNa2	-0.043	-0.083	-0.009	
208.92010	C2H6O4Ca2Cl	-0.043			
208.96455	C2H9O6Ca2		0.148		
208.97980	C6H9O3Ca2	-2.780			
208.98392	HPb			1.333	
209.02107	C8H10O4K		0.059		
209.02600	C16H3N	-1.946	0.116	0.029	0.005
209.05745	C9H14O3K	-0.042	0.042	0.058	-0.038
209.06964	C6H17O5Ca		0.142		
209.07843	C9H14O4Na		0.046	0.002	
209.09384	C10H18O2K		-0.027	-1.318	
209.09609	C15H13O				0.045
209.10732	C14H13N2			-0.007	
209.11482	C10H18O3Na	-0.047	0.082	0.051	
209.11990	C15H15N	0.040		-0.006	-0.035
209.15361	C13H21O2			-0.187	-0.125
209.91361	O6N2KMgNa	-0.828			
209.99236	C10H3O4Na		-1.098		
210.02851	C4H12O6NCa	0.187			
210.10392	C15H14O	0.126			
210.11515	C14H14N2			0.066	
210.86304	CH2O3K3S	-1.137	-1.261		
210.99139	C7H7O5Ca	0.002			
210.99454	C2H3O8N4	0.031			
211.03672	C8H12O4K		-0.217		
211.05423	C17H7	0.419			0.077
211.05770	C8H12O5Na			-0.172	
211.06416	C9H15O3Ca		-0.181		
211.07310	C9H16O3K		-0.210	-0.134	

211.08659	C13H11ON2			0.013	
211.09408	C9H16O4Na		-0.173		
211.12297	C14H15N2	0.079	-0.175	-0.014	-0.018
211.16926	C13H23O2			-0.112	
211.89105	H4O7Ti2	-0.659		-0.993	-0.845
211.93906	H6O7NCa2		-0.175		
212.01587	C6H12O5Ti	-0.016			
212.03181	C10H7O3NNa	0.456		0.423	
212.05285	C13H10NS	0.687			
212.06542	C14H12S	0.229			
212.09441	C13H12ON2			0.000	-0.023
212.11822	C13H14N3	0.074	-0.289	0.053	
212.84231	K3SO4	-0.045	-0.386	-0.059	-0.059
212.85194	C2HO2K4	-0.599	-0.941		
212.90175	H7O5NFe2	0.115			
212.91480	C5H2O3Ca2Na	0.886			
213.00704	C7H9O5Ca	0.027	-0.291		
213.01598	C7H10O5K		-0.390		
213.04343	C8H13O4Ca	0.008	-0.359		
213.05237	C8H14O4K		-0.444	-0.193	
213.05462	C13H9O3				-0.032
213.06988	C17H9	0.069	-0.290	-0.047	-0.021
213.09101	C14H13O2		-0.394		0.060
213.10224	C13H13ON2		-0.357	0.081	0.017
213.14612	C10H22O3Na	-0.034		-0.016	-0.053
213.18491	C13H25O2			-0.141	
213.84725	O3NClK3		-0.492		
213.84731	HO5K2Mn			-0.287	
213.88577	H6O6Fe2	0.008		0.046	-0.051
214.05125	C8H14O4Ca	-0.038			
214.07770	C17H10				-0.024
214.09749	C12H12ON3			-0.022	
214.89359	H7O6Fe2	-0.225	-0.433	-0.279	-0.162
214.91453	H7O7Ti2	-0.015		-0.069	-0.093
214.92856	C2H5O3Ba		0.137	0.029	-0.015
214.95401	C3H7ON2AlK2Na	-0.150			
214.97171	C7H5O4KNa	-1.517			
215.01276	C15H3O2	0.004	-0.485	-0.098	-0.073
215.02269	C7H11O5Ca		-0.462		
215.03163	C7H12O5K		-0.695		
215.03389	C12H7O4		-0.388		0.016
215.05908	C8H15O4Ca	0.083	-0.608		
215.06787	C11H12O3Na		0.019	0.485	
215.08150	C12H11O2N2			-0.040	
215.08553	C17H11	0.111	0.372	-0.012	-0.021
215.08900	C8H16O5Na		0.395		
215.09546	C9H19O3Ca	0.014			

215.90142	H8O6Fe2	-0.015	0.309	0.010	-0.046
215.99413	C6H8O6Ca	-1.936			
216.04171	C12H8O4				1.447
216.06690	C8H16O4Ca		0.042		
216.09335	C17H12	0.032	0.290	0.039	0.011
216.99549	C2H10O8AlSi			-0.010	
217.02615	C10H10O3K		0.132		
217.03834	C7H13O5Ca	0.027	0.256		
217.04713	C10H10O4Na		0.624		
217.04730	C7H14O5K			-0.522	
217.04954	C12H9O4				-0.005
217.07335	C12H11O3N			1.771	
217.07473	C8H17O4Ca	0.046	0.194		
217.08860	C16H11N	0.050	0.166	0.044	
217.10118	C17H13		0.340		0.134
217.10465	C8H18O5Na	-0.019	0.138	0.063	-0.003
217.15629	C13H22ONa	0.032	0.158		
217.90307	H2O4NBa			0.143	0.094
218.01510	C18H2	0.045	0.097	-0.004	
218.03359	C6H12O5NCa	0.187			
218.05736	C12H10O4				1.063
218.07262	C16H10O	-0.005	0.113	0.103	0.035
218.08117	C12H12O3N			0.071	0.008
218.09643	C16H12N	0.096	0.080		
218.10900	C17H14				-0.069
218.93629	C5H3O3NKNaS			0.377	
218.94890	C3H7O6Ca2	-0.006			
218.95677	H8O8AlSi2			0.026	
219.01761	C6H11O6Ca	-0.028	0.009		
219.02655	C6H12O6K	0.013	-0.046	-0.059	-0.157
219.04180	C10H12O3K		-0.138		
219.05399	C7H15O5Ca	0.063			
219.08044	C16H11O	0.406			
219.09038	C8H19O4Ca	0.027	2.265		
219.09167	C15H11N2			-0.207	
219.11683	C17H15				0.041
219.88801	H4O6Ca3	0.026			
220.01286	C5H10O6NCa		0.036		
220.03925	C7H16O4Fe	0.117			
220.06182	C7H16O5Ca	0.090			
220.09682	C12H14O3N			-0.097	0.021
220.11208	C16H14N		-0.054	0.017	
220.12465	C17H16	0.099	-0.063		
221.00093	C4H13O5Ca2	-0.251			
221.02107	C9H10O4K		-0.474		
221.04220	C6H14O6K	-0.056	-0.389	-0.493	-0.567
221.05745	C10H14O3K		-0.217		

221.06964	C7H17O5Ca	-0.033			
221.09384	C11H18O2K		-0.246		
221.09609	C16H13O				0.198
221.10732	C15H13N2			0.043	
221.11482	C11H18O3Na			0.161	
221.12340	C8H21O5Mg	-0.001			
222.01852	C6H14O5Fe	-0.056			
222.07747	C7H18O5Ca		-0.206		
222.12773	C16H16N		-0.216	0.070	
222.86704	H4O4Ca3Cl	0.234			
222.91149	H7O6Ca3	-0.012			
223.03672	C9H12O4K		-0.419		
223.04891	C6H15O6Ca	0.033			
223.07310	C10H16O3K	0.006	-0.295	0.027	
223.07568	C7H16O6Al	-1.235			
223.09408	C10H16O4Na		-0.300	0.118	
223.10949	C11H20O2K		-0.346	-0.003	
223.11207	C16H15O	1.266		-1.231	
223.12297	C15H15N2			0.038	
223.13555	C16H17N	0.078		0.011	
223.92907	C2H8O6CaFe	0.045			
224.04027	C13H8N2S	-0.704			
224.06205	C18H8	0.006	-0.431	-0.018	
224.11957	C16H16O	0.001			
224.15595	C17H20		-0.359		
224.87130	O5N2K3		-0.391		
224.89227	O6N2K2Na	-0.076	-0.534	0.020	
224.97883	HOPb	0.276	-0.403	0.036	0.003
225.01019	C4H13O6CaSi	0.142			
225.01598	C8H10O5K		-0.462		
225.05237	C9H14O4K		-0.477	0.005	
225.07981	C10H17O3Ca		-0.406		
225.08875	C10H18O3K		-0.527	-0.205	
225.10224	C14H13ON2			0.040	
225.10973	C10H18O4Na		-0.425		
225.13862	C15H17N2			0.045	
225.88577	CH6O6Fe2	0.087		0.201	
226.05125	C9H14O4Ca	0.004	-0.441		
226.05592	C13H10N2S	-0.722			
226.07770	C18H10	-0.018	-0.517	-0.002	-0.022
226.08107	C15H14S	0.251			
226.14645	C15H18N2			0.040	
226.93045	C6H4O3Ca2Na	0.888			
226.96494	C4H9O2Ba	0.403			
227.03163	C8H12O5K		-0.656		
227.03389	C13H7O4				0.106
227.05261	C8H12O6Na		-0.608	-0.003	

227.05908	C9H15O4Ca	-0.024	-0.608		
227.06802	C9H16O4K		-0.679		
227.08553	C18H11	0.069	-0.080	-0.007	-0.067
227.11789	C14H15ON2			-0.002	-0.052
227.17540	C12H23O2N2				-0.189
227.93638	C3H6O3Ba	0.146			
227.99413	C7H8O6Ca	0.216			
227.99945	C19		-0.679		
228.00575	C10H7O3NK	-0.073		0.279	
228.04171	C13H8O4		0.943		
228.06311	C11H11O3NNa			0.377	
228.06690	C9H16O4Ca	0.137			
228.09335	C18H12	-0.012	-0.642	-0.034	-0.062
229.00196	C7H9O6Ca	0.450	0.652		3.442
229.00728	C19H	0.079	0.176	0.041	-0.002
229.02615	C11H10O3K		0.141		
229.03834	C8H13O5Ca		0.009		
229.04954	C13H9O4		0.044		0.046
229.06479	C17H9O	0.332			
229.07473	C9H17O4Ca	0.022	0.170		
229.08352	C12H14O3Na		0.702	0.790	
229.09715	C13H13O2N2	-1.449	-1.193	-0.389	-0.371
229.10118	C18H13	0.349	0.309		0.099
229.10465	C9H18O5Na		0.283		
229.11111	C10H21O3Ca	0.074			
229.12005	C10H22O3K	-0.017	0.073	0.040	-0.016
229.14103	C10H22O4Na			0.224	
229.16992	C15H21N2		0.123		
229.90162	H6O8Ti2	-0.019		0.015	-0.101
230.01510	C19H2		0.068		
230.09240	C12H12O2N3			-0.002	
230.09643	C17H12N	0.134	0.105		
230.10900	C18H14	0.060	0.122	0.034	0.016
230.92999	H7O8CaSi2				0.031
230.98528	C5H11O5Ca2	-0.085			
231.00767	C15H3O3		0.053		
231.02293	C19H3	0.205	-0.012		
231.02880	C12H7O5				0.477
231.04180	C11H12O3K		-0.130		
231.05399	C8H15O5Ca		0.069		
231.06293	C8H16O5K		-0.182		
231.08044	C17H11O	0.175	0.086	0.049	
231.09038	C9H19O4Ca	0.006	0.142		
231.09932	C9H20O4K		-0.066		
231.10425	C17H13N	0.075	0.037		
231.11683	C18H15	1.711	0.072		1.326
232.00287	C7H12O5Fe	0.353			

232.08827	C17H12O	0.039	0.015	0.185	
232.09682	C13H14O3N			-0.129	
232.11208	C17H14N	0.147	-0.012		
232.12465	C18H16				0.015
232.89584	CH5O6Ca3	0.122			
232.94564	H9O8CaSi2			0.097	
233.02107	C10H10O4K		-0.108		
233.02600	C18H3N		-0.040	0.026	0.013
233.04220	C7H14O6K		-0.259		
233.04445	C12H9O5				0.199
233.04708	C8H17O4Fe	1.384			
233.05745	C11H14O3K		-0.182		
233.06964	C8H17O5Ca	0.024			
233.07858	C8H18O5K	-0.100	-0.213	-0.050	-0.074
233.09384	C12H18O2K		-0.247		
233.10603	C9H21O4Ca	-0.062	0.383		
233.13022	C13H22OK	0.037	-0.128		
233.13248	C18H17				-0.065
233.89109	H4O6NCa3	0.065			
234.00470	C6H10O7Ca		-0.180		
234.04987	C10H11O5Na	-0.080			
234.05490	C8H18O4Fe	0.347			
234.12773	C17H16N		-0.144		
234.91023	C5H3O3NK2S			0.325	
234.95168	H8O9AlSi2			0.029	-0.011
235.03672	C10H12O4K		-0.315		
235.05785	C7H16O6K	0.148			
235.06273	C8H19O4Fe	1.960			
235.07310	C11H16O3K		-0.235		
235.08529	C8H19O5Ca	-0.027	-0.027		
235.10949	C12H20O2K		-0.231		
235.13047	C12H20O3Na		-0.202	0.178	
235.16926	C15H23O2			-0.165	
235.91364	H4O5NBa			0.144	
236.04416	C6H14O6NCa	0.077			
236.09174	C12H14O4N			0.191	
236.11049	C8H20O6Mg	0.073			
236.14338	C17H18N		-0.153		
236.99485	C12H6O3K		-0.383		
237.02817	C6H13O7Ca		-0.240		
237.04810	C14H9N2S	-1.857			
237.05237	C10H14O4K		-0.389		
237.06456	C7H17O6Ca	0.121			
237.06988	C19H9	-0.001	-0.406	-0.032	-0.072
237.08875	C11H18O3K		-0.389		
237.10094	C8H21O5Ca	0.138		-1.937	
237.10224	C15H13ON2			0.117	

237.10973	C11H18O4Na		-0.322		
237.12514	C12H22O2K		-0.352		
237.13862	C16H17N2			0.063	
237.18491	C15H25O2			-0.172	
237.87601	H6O6Ca2Fe	0.064			
237.89858	H6O7Ca3	0.064			
238.89359	C2H7O6Fe2	0.153		0.177	
239.01050	C12H8O3K		-0.478		
239.03163	C9H12O5K		-0.516		
239.05908	C10H15O4Ca		-0.467		
239.06802	C10H16O4K		-0.509	0.003	
239.08553	C19H11	-0.030	-0.526	-0.048	-0.090
239.08900	C10H16O5Na		-0.559	-0.140	
239.09661	C8H20O4FCa	-3.673			
239.10440	C11H20O3K		-0.509		
239.90142	C2H8O6Fe2	0.009		0.095	-0.027
239.99945	C20	-0.062		0.010	0.032
240.06690	C10H16O4Ca	-0.041	-0.536		
240.09335	C19H12	0.009	-0.490		-0.046
240.86621	O6N2K3	-0.106	-0.625	0.016	-0.067
240.87760	H6O5Ca3Cl	0.064			
241.00246	C16H3Na2	0.741	0.435	1.393	1.375
241.04728	C9H14O5K		-0.641		
241.07232	C8H18O4CaNa	0.396			
241.07473	C10H17O4Ca		-0.682		
241.08367	C10H18O4K		-0.654		
241.08860	C18H11N	-0.035	-0.662		
241.10118	C19H13	0.085	-0.455		-0.076
242.00978	C8H10O6Ca	-0.343			
242.01510	C20H2	0.000	-0.653	-0.102	
242.04238	C11H9O4NNa		-0.067		
242.07262	C18H10O	0.041	-0.592		
242.10900	C19H14	0.008	-0.609	-0.054	-0.076
242.94495	ClPb				0.244
242.94565	C8H5O4K2		0.007	-2.086	
242.98940	H3O2Pb			0.253	0.096
243.02655	C8H12O6K		0.170	-0.047	
243.02880	C13H7O5				0.349
243.04180	C12H12O3K		0.083		
243.06053	C7H17O5KNa			0.291	
243.06293	C9H16O5K		0.050		
243.08044	C18H11O	0.174			
243.09038	C10H19O4Ca		0.329		
243.09917	C13H16O3Na			0.679	
243.10425	C18H13N	0.075			
243.11280	C14H15O2N2	-1.299			
243.11683	C19H15	0.178	0.242		0.038

243.12030	C10H20O5Na		0.251		
244.03705	C11H11O3NK		-0.001	-0.168	-0.270
244.08827	C18H12O	0.130			
244.11208	C18H14N		0.190		
244.12465	C19H16	0.060	0.174	-0.002	-0.024
244.96462	C2H8O8KNa2			-0.119	
245.00093	C6H13O5Ca2	-0.074			
245.04220	C8H14O6K		-0.019		
245.04445	C13H9O5				0.268
245.06964	C9H17O5Ca	0.028	0.046		
245.07843	C12H14O4Na		0.474		
245.09609	C18H13O	0.289	0.164		
245.10603	C10H21O4Ca	-0.029			
245.11497	C10H22O4K	-0.180		-0.026	
245.11990	C18H15N	0.061	0.106		
245.13248	C19H17				0.066
245.13595	C10H22O5Na		0.147	0.289	
245.15120	C14H22O2Na	0.041	0.106		
246.01002	C19H2O	0.037	0.087		-0.056
246.04640	C20H6				-0.146
246.10392	C18H14O	0.021	0.113	-0.013	
246.14030	C19H18	0.050			
246.91149	C2H7O6Ca3	0.075			
247.01252	C7H11O7Ca	0.690	1.790		
247.03672	C11H12O4K		-0.174		
247.08529	C9H19O5Ca	-0.063			
247.12168	C10H23O4Ca	-0.148	-0.086		
248.03417	C8H16O5Fe	0.297			
248.06205	C20H8	-0.026			
248.06552	C11H13O5Na			0.025	
248.94056	H9O9CaSi2			-0.006	-0.080
249.05237	C11H14O4K		-0.180		
249.08875	C12H18O3K		-0.157		
249.11831	C9H21O6Mg	0.128			
249.12514	C13H22O2K		-0.121		
249.15656	C13H24Na3		3.057	3.278	
250.05981	C7H16O6NCa	0.108			
250.07770	C20H10	-0.076	-0.219	-0.106	-0.109
250.88468	H2O6Na3Si3	-1.701			
251.04689	C14H12O2K		-0.352		
251.05276	C7H16O7K		-0.918		
251.06802	C11H16O4K		-0.201		
251.08553	C20H11	0.080			-0.054
251.10440	C12H20O3K		-0.214		
251.12538	C12H20O4Na		-0.198		
251.14079	C13H24O2K		-0.214		
251.96579	H10O9NSi3			-0.076	-0.109

252.01526	C6H12O8Ca		-0.175		
252.06547	C8H20O5Fe	0.342			
252.08803	C8H20O6Ca	0.382			
252.09335	C20H12	-0.090	-0.223	-0.057	-0.068
252.09923	C13H16O5			1.356	
252.94981	H9O10Si3			-0.068	-0.120
252.97375	CHO2Pb			-0.320	-0.122
253.00728	C21H		-0.298	-0.102	
253.04728	C10H14O5K		-0.335		
253.07473	C11H17O4Ca	-0.097	-0.331		
253.08367	C11H18O4K		-0.335		
253.10118	C20H13	-0.026	-0.165		-0.130
253.12005	C12H22O3K		-0.320		
253.13354	C16H17ON2		-0.312		
253.16992	C17H21N2			0.092	
254.01510	C21H2		-0.417		
254.02504	C13H10O3Ca	-0.258			
254.09643	C19H12N	0.014	-0.355		
254.10900	C20H14	-0.010	-0.300	-0.099	-0.089
254.16517	C16H20N3		-0.305		
255.02293	C21H3		-0.268	-0.034	
255.06053	C8H17O5KNa			0.787	
255.06278	C13H12O4Na			0.485	
255.06293	C10H16O5K		-0.441		
255.06519	C15H11O4	0.014			-0.110
255.06706	C8H20O4CaCl	0.030			
255.08044	C19H11O	0.006	-0.453	-0.142	
255.09932	C11H20O4K		-0.336		
255.10425	C19H13N	-0.021			
255.11683	C20H15	0.159	-0.457		-0.065
255.13570	C12H24O3K		-0.430		
255.14919	C16H19ON2		-0.364		
256.01286	C8H10O6NCa	0.276			
256.08827	C19H12O	0.014	-0.371		
256.09682	C15H14O3N			0.093	
256.12465	C20H16	-0.002	-0.379		-0.095
256.92119	H6O9NaSi3			-0.118	-0.141
257.02025	C10H17Ca3	3.060			
257.02600	C20H3N		-0.381	0.009	-0.100
257.04220	C9H14O6K		-0.517		
257.04445	C14H9O5				0.317
257.05971	C18H9O2	0.955			
257.07858	C10H18O5K		-0.572		
257.12845	C15H17O2N2	-1.258	-1.125		
257.13248	C20H17	0.154			-0.038
257.13595	C11H22O5Na		-0.382		
258.04987	C12H11O5Na	-0.060			

258.05774	C8H18O6Ti	0.157			
258.06753	C18H10O2	0.103			
258.10392	C19H14O	0.103	0.487		
258.14030	C20H18	0.049	0.394		
259.01252	C8H11O7Ca	0.465	0.556	2.537	
259.09408	C13H16O4Na		0.829		
259.09423	C10H20O5K			-0.016	
259.11174	C19H15O	0.299			
259.11536	C7H24O7K		-0.221		
259.13555	C19H17N	0.041			-0.035
259.25464	C14H37NCa		1.389		
259.96260	C4H10O4Ba	0.029			
260.03197	C11H11O4NK	-0.198			
260.05310	C8H15O6NK		0.046		
260.11957	C19H16O	0.013	0.364		
260.14338	C19H18N		0.317		
260.15595	C20H20	0.071			
261.02817	C8H13O7Ca	0.135	0.441		
261.03711	C8H14O7K		0.222		
261.03937	C13H9O6				0.803
261.05237	C12H14O4K		0.191		
261.06988	C21H9	0.243			-0.133
261.07350	C9H18O6K		-0.008		
261.08875	C13H18O3K		0.325		
261.10988	C10H22O5K		0.014		
261.12514	C14H22O2K		0.244		
261.13086	C10H22O6Na		0.248	0.350	
261.13862	C18H17N2			0.055	
261.85059	H4O4BaFe				-0.080
262.01081	C12H6O7		1.844		
262.04719	C13H10O6		1.725		1.466
262.07368	C16H10O2N2			-1.103	-1.260
262.08117	C12H15O5Na			-0.088	
262.09883	C18H14O2		0.347		
263.01050	C14H8O3K		0.150		
263.05276	C8H16O7K		-0.056		
263.06802	C12H16O4K		0.126		
263.08021	C9H19O6Ca	0.065			
263.08150	C16H11O2N2			0.018	-0.250
263.08553	C21H11	-0.087	0.103	-0.168	-0.145
263.10440	C13H20O3K		0.168		
263.11789	C17H15ON2			0.034	
263.12538	C13H20O4Na		0.247		
263.13396	C10H23O6Mg	-0.034			
263.90456	H12O7Fe2Si			1.900	1.860
263.99945	C22	-0.050	0.166	-0.148	-0.105
264.00269	C6H10O8NCa		-0.115		

264.01833	C14H9O3K	-1.167			
264.09335	C21H12	-0.057	0.108		-0.136
264.12304	C14H18O4N				0.180
264.91239	H13O7Fe2Si			1.892	
264.92760	C8H4O4K2Na		0.125		
265.02309	C7H13O8Ca		0.702		
265.04713	C14H10O4Na		0.584		
265.08367	C12H18O4K		0.063		
265.08860	C20H11N	-0.518			
265.09586	C9H21O6Ca	0.346			
265.10118	C21H13	-0.054	0.052		-0.144
265.12005	C13H22O3K		0.067	-0.044	
265.12231	C18H17O2	0.942			
265.13050	C13H24KNa2		3.095	2.891	
265.13129	C12H22O2N2K	2.892			-0.040
265.15644	C14H26O2K		0.097		
266.01510	C22H2	-0.021	0.005	-0.182	
266.09111	C8H20O6NCa	0.021			
266.09643	C20H12N	-0.006			
266.10900	C21H14	-0.085	0.072	-0.180	-0.123
266.11894	C13H22O3Ca	0.009	0.181		
266.98940	C2H3O2Pb			0.087	-0.032
267.00542	C13H8O4K	-0.048			
267.06293	C11H16O5K		-0.031		
267.08044	C20H11O	0.124	0.010		
267.09038	C12H19O4Ca	-0.183			
267.09932	C12H20O4K		-0.016	-0.098	
267.10425	C20H13N	-0.104	-0.151		
267.11683	C21H15	0.177	0.717		-0.069
267.13570	C13H24O3K		-0.035		
267.17170	C10H29O4NCa	0.996		0.906	
268.08827	C20H12O	-0.075	-0.011		-0.060
268.11208	C20H14N		-0.067		
268.12465	C21H16	-0.056	-0.130		-0.176
268.13321	C17H18O2N			-0.072	
268.18082	C17H22N3		-0.082		
269.02025	C11H17Ca3	2.902			
269.03376	C18H7Na2	-1.741			
269.04205	C13H10O5Na		0.449		
269.07858	C11H18O5K		-0.135		
269.09609	C20H13O	0.095			
269.13248	C21H17	0.144			-0.066
270.10392	C20H14O	-0.025	-0.073		
270.14030	C21H18	-0.039	-0.140		
270.85110	H7O7CaFe2	-0.092			
270.98431	CH3O3Pb			0.670	
271.05785	C10H16O6K		-0.276		

271.09423	C11H20O5K		-0.236		
271.14410	C16H19O2N2	-1.206			
271.14813	C21H19	0.277			
271.15160	C12H24O5Na		-0.115		
271.85893	H8O7CaFe2	0.006			
272.03154	C14H8O6		1.275		1.343
272.03560	C13H12O4Ca	-0.159			
272.03607	C5H10O10N3		-1.938		
272.11957	C20H16O		-0.214		
272.15595	C21H20	-0.060	-0.233		
272.89513	H6O9KSi3			-0.227	-0.209
273.03937	C14H9O6	-1.083			0.013
273.05237	C13H14O4K		-0.317		
273.10973	C14H18O4Na		0.218		
273.15120	C20H19N	-0.069			
273.16725	C12H26O5Na		-0.222	-0.049	
274.04132	C21H6O				1.246
274.07770	C22H10	-0.219	-0.484	-0.337	-0.275
274.13522	C20H18O	-0.063	-0.244		
275.04770	C14H13ONa2S	-0.227			
275.06802	C13H16O4K		-0.367		
275.08900	C13H16O5Na		0.102	0.151	
275.14079	C15H24O2K		-0.309		
275.99945	C23	-0.177	0.512	-0.177	-0.088
276.09335	C22H12	-0.130	0.529	-0.186	-0.159
276.09682	C13H17O5Na	-0.949		-0.769	
276.11448	C19H16O2		0.503		
276.93275	H8O10Al3Si			3.341	
276.95844	C4H13O6Ca3	0.042			
277.00728	C23H	-0.067	0.466	-0.240	-0.258
277.05809	C13H11O6N		2.404		
277.08367	C13H18O4K		0.494		
277.09586	C10H21O6Ca	1.933			
277.10118	C22H13	-0.023	0.523		-0.145
277.10480	C10H22O6K		0.310	-0.451	
277.12005	C14H22O3K		0.505		
278.01510	C23H2		0.389		
278.09643	C21H12N	0.079	0.463		
278.10900	C22H14	-0.147	0.402	-0.268	-0.201
278.19033	C20H24N			-0.047	
278.86891	C2H5ON4Ca3Ni	0.389			
278.91109	H7O10Si4				-0.176
279.02293	C23H3		0.631	-0.106	
279.03874	C8H15O8Ca		0.666		
279.06278	C15H12O4Na		0.931		
279.08044	C21H11O	0.055			
279.09932	C13H20O4K		0.447		

279.11151	C10H23O6Ca	0.385			
279.11280	C17H15O2N2		-1.330	-0.763	
279.11683	C22H15	-0.016	0.626		-0.109
279.13570	C14H24O3K		0.429		
279.15909	C16H23O4	-0.109		-0.110	
279.17209	C15H28O2K		0.447		
280.11208	C21H14N		0.367		
280.12465	C22H16	-0.140	0.310		-0.236
281.02600	C22H3N		0.417	-0.074	
281.07858	C12H18O5K		0.334		
281.09956	C12H18O6Na		0.420		
281.11497	C13H22O4K		0.355		
281.11990	C21H15N	-0.113			
281.13248	C22H17	0.165			-0.045
281.15135	C14H26O3K		0.337		
282.01002	C22H2O		0.554	-0.140	
282.02006	C5H13O6N2KNa2	-0.706			
282.02889	C14H11O4K	-0.494			
282.08602	C8H20O7NCa	0.148			
282.10392	C21H14O	-0.089	0.340		
282.14030	C22H18	-0.100	0.156		
282.85160	O5N4KNaRb		-2.183		
283.02131	C13H8O6Na		0.810		-0.493
283.05785	C11H16O6K		0.255		
283.07536	C20H11O2	0.237			
283.09423	C12H20O5K		0.259		
283.13062	C13H24O4K		0.283		
283.14813	C22H19	0.283			0.163
284.05535	C15H10O5N				0.032
284.06519	C19H12OSi	0.914			
284.08318	C20H12O2	0.037			
284.10431	C17H16O4		1.054		
284.10699	C20H14ON		0.318		
284.11957	C21H16O		0.269		
284.12812	C17H18O3N			-0.033	-0.021
284.15595	C22H20	-0.076			
284.99996	C2H5O3Pb			0.523	0.189
285.03696	C13H10O6Na		0.767		
285.05237	C14H14O4K		0.206		
285.06988	C23H9		0.311		-0.241
285.07575	C16H13O5	0.554			
285.10988	C12H22O5K		0.170		
285.13595	C17H19O3N			-1.274	
285.81562	H6O7Fe3	-0.121	0.344	-0.184	-0.192
286.04719	C15H10O6		1.587		
286.13522	C21H18O		-0.165		
286.14377	C17H20O3N			-0.063	-0.089

286.17160	C22H22	-0.076			
286.82345	H7O7Fe3	-0.106		-0.082	-0.140
287.01050	C16H8O3K		0.203		
287.08553	C23H11	-0.131	0.133		-0.276
287.08915	C11H20O6K		0.038		
287.12538	C15H20O4Na		0.599		
287.14666	C9H28O7K		-0.321		
287.15298	C13H27O4Ca	-0.148			
287.17717	C17H28OK		0.222		
287.99945	C24	-0.254	0.141	-0.255	-0.142
288.02646	C14H8O7		1.592		
288.07810	C19H12O3	0.128			
289.10118	C23H13	-0.121	0.096	-0.282	-0.272
289.10480	C11H22O6K		-0.036		
289.12005	C15H22O3K		0.137		
289.14118	C12H26O5K		-0.071	-0.410	
289.15365	C10H28O4NCaNa	0.854			
289.16216	C12H26O6Na			0.058	
290.01510	C24H2	-0.058	0.028	-0.359	
290.04211	C14H10O7		1.452		1.005
290.06592	C14H12O6N				-0.078
290.10900	C23H14	-0.199	0.000		-0.267
291.06293	C13H16O5K		0.005		
291.09932	C14H20O4K		0.039		
291.10425	C22H13N	-0.246			
291.11683	C23H15	0.012	0.217		-0.169
291.13570	C15H24O3K		0.056		
291.15668	C15H24O4Na		0.107		
292.08827	C22H12O	-0.145	0.037		
292.12465	C23H16	-0.244	-0.145		-0.300
293.07843	C16H14O4Na		0.461		
293.10507	C16H18ON2K		2.931		
293.11497	C14H22O4K		-0.010		
293.12716	C11H25O6Ca	0.565			
293.13248	C23H17	0.084			-0.075
293.13595	C14H22O5Na		0.075		
293.15135	C15H26O3K		-0.010		
293.20872	C16H30O3Na		0.034		
293.89592	O9N3KNa3		-0.007		
294.07609	C17H12O4N	-0.099			
294.14030	C23H18	-0.276	-0.600		
294.90600	H7O11Si4			-0.228	-0.212
295.02131	C14H8O6Na		1.275		0.209
295.03672	C15H12O4K	-0.359			
295.09423	C13H20O5K		0.789		
295.11521	C13H20O6Na		0.966		
295.13062	C14H24O4K	-0.108	0.803		

295.13555	C22H17N	-0.149			
295.14813	C23H19				0.178
295.16930	C14H27O2N2Ca	0.857			
296.10699	C21H14ON		0.937		
296.11957	C22H16O	-0.054	0.711		
296.15595	C23H20	-0.142	0.369		
296.83460	C5H2O2NCa2Cl2K	1.555			
297.03696	C14H10O6Na		1.296		
297.07350	C12H18O6K		0.707		
297.10988	C13H22O5K		0.720		
297.14627	C14H26O4K		0.737		
297.95329	H12O10NSi4			-0.192	-0.183
298.07770	C24H10	-0.257	0.015		-0.331
298.11996	C18H18O4		1.346		
298.13522	C22H18O		0.598		
298.14377	C18H20O3N			-0.088	-0.070
298.17160	C23H22	-0.110			
298.34683	C20H44N		0.738		
298.82554	O5N4K2Rb		-1.652		
299.05276	C11H16O7K				-0.247
299.12553	C13H24O5K		0.625		
300.09335	C24H12	-0.276	0.518	-0.392	-0.306
300.09965	C15H19O3NK		0.355		
300.15942	C18H22O3N			-0.195	-0.095
300.99245	C19H4ONK	-4.577			
301.00728	C25H	-0.032	0.672	-0.273	
301.01090	C13H10O6K		0.638		
301.03765	C12H13O7S	2.038			
301.10118	C24H13		0.821		-0.181
301.10480	C12H22O6K		0.578	-0.415	
301.14103	C16H22O4Na	-0.298	0.744		-0.218
301.14118	C13H26O5K			-0.844	
301.16231	C10H30O7K		0.146		
302.10900	C24H14	-0.284	0.542	-0.404	-0.309
303.04262	C15H13O2Na2S	-1.563			
303.04812	C21H11Ca		-0.834		
303.09932	C15H20O4K		0.611		
303.11683	C24H15	-0.022	0.779		-0.177
303.12045	C12H24O6K		0.472	-0.462	
303.14158	C9H28O8K		0.129		
303.17781	C13H28O6Na			-0.009	
303.85292	O9N3CaK2		0.570		
304.11208	C23H14N	0.004	0.650		
304.12465	C24H16	-0.276	0.302		
304.15434	C17H22O4N			-0.122	-0.032
304.24824	C16H34O4N			-0.337	
305.07858	C14H18O5K		0.516		

305.08431	C10H18O9Na			-1.163	
305.09609	C23H13O	0.020	0.624		
305.09971	C11H22O7K		0.408		
305.11497	C15H22O4K		0.549		
305.11990	C23H15N	-0.265			
305.12780	C11H21O4N4S		0.844	0.290	0.171
305.13248	C24H17	-0.137			-0.363
305.13610	C12H26O6K		0.250	-0.555	
305.15135	C16H26O3K		0.542		
305.15708	C12H26O7Na		0.552	0.143	
306.10392	C23H14O	-0.176	0.392		
306.14030	C24H18	-0.324	0.016		
307.05770	C16H12O5Na	0.110			
307.05785	C13H16O6K		0.442		
307.09408	C17H16O4Na		0.953		
307.12072	C17H20ON2K		2.239		
307.13062	C15H24O4K		0.455		
307.13555	C23H17N	-0.372			
307.14813	C24H19	0.221			0.215
307.16700	C16H28O3K		0.458		
308.06519	C21H12OSi	0.424			
308.08318	C22H12O2	-0.053			
308.11957	C23H16O		0.154		
308.12571	C17H19O3NNa			-0.102	
308.15595	C24H20	-0.316	-0.528		
309.07350	C13H18O6K		0.379		
309.10095	C11H23O2N2KNaS	0.094			
309.10988	C14H22O5K		0.408		
309.12207	C11H25O7Ca		-0.063		
309.14627	C15H26O4K		0.401		
309.15846	C12H29O6Ca	0.050			
309.18265	C16H30O3K		0.401		
309.20363	C16H30O4Na			-0.450	
309.86986	O9N3K2Na2	-0.317	0.371	-0.204	
310.13522	C23H18O	-0.231	-0.368		
310.14377	C19H20O3N			-0.219	-0.185
310.17160	C24H22	-0.251			
311.08553	C25H11	-0.144			-0.300
311.08915	C13H20O6K		0.310		
311.12553	C14H24O5K		0.345		
311.93255	H10O11NSi4			-0.374	-0.282
311.99945	C26	-0.346	0.334	-0.469	-0.310
312.09335	C25H12		-1.730		
312.18725	C24H24	-0.142			
312.36248	C21H46N			-0.261	-0.229
313.03188	C14H10O7Na	0.335	0.716		-0.054
313.10118	C25H13	-0.189	0.329		-0.302

313.20501	C16H33O3Ca	-0.209	0.367		
313.94820	H12O11NSi4			-0.354	-0.300
314.01510	C26H2	-0.103	0.273		
314.10900	C25H14	-0.362	-0.202		
314.11530	C16H21O3NK		-0.039		
314.17507	C19H24O3N			-0.255	
314.79945	H2O3N4KRb2		-1.862		
315.04753	C14H12O7Na		0.792		
315.11683	C25H15	-0.079	0.493		-0.238
315.12045	C13H24O6K		0.160		
315.13570	C17H24O3K		0.293		
316.12465	C25H16	-0.105	0.415		
317.09971	C12H22O7K		0.590		
317.11497	C16H22O4K	-0.169	0.643	-0.261	-0.066
317.13248	C25H17				0.243
317.13610	C13H26O6K		0.542		
318.10392	C24H14O	0.007	0.505		
318.14030	C25H18	-0.132	0.046		
319.09423	C15H20O5K		0.588		
319.11536	C12H24O7K		0.609	-0.147	
319.13062	C16H24O4K		0.600		
319.13555	C24H17N	-0.114			
319.14813	C25H19				0.046
319.15175	C13H28O6K		0.352	-0.366	
320.11957	C24H16O	-0.046	0.248		
320.15595	C25H20	-0.149			
321.05824	C10H18O9K			-0.770	
321.07350	C14H18O6K		0.524		
321.10973	C18H18O4Na		0.997		
321.13101	C12H26O7K		0.374	-0.330	
321.14627	C16H26O4K		0.542		
321.15120	C24H19N	-0.128			
321.18265	C17H30O3K		0.539		
321.24002	C18H34O3Na		0.573		
322.13522	C24H18O		-0.640		
322.14136	C18H21O3NNa			-0.116	
322.17160	C25H22	-0.122			
323.08915	C14H20O6K		0.451		
323.12553	C15H24O5K		0.476		
323.16192	C16H28O4K		0.479		
323.19830	C17H32O3K		0.482		
323.99945	C27	-0.213	0.440	-0.343	-0.158
324.09335	C26H12	-0.263	-0.362		-0.291
324.09965	C17H19O3NK			-0.851	
324.12304	C19H18O4N	-0.124			
324.18725	C25H24	-0.050			
325.00728	C27H	0.029	0.485	-0.356	-0.301

325.06826	C16H14O6Na		0.915		
325.10480	C14H22O6K		0.395		
325.14118	C15H26O5K		0.416		
325.17757	C16H30O4K		0.410	-0.378	
325.84380	O9N3K3Na		0.395	-0.074	
326.10900	C26H14	-0.240	0.056	-0.371	-0.217
326.13869	C19H20O4N			-0.092	
326.37813	C22H48N		0.478		
327.11683	C26H15	0.031	0.648		-0.083
328.12465	C26H16	-0.184	0.039		
328.13095	C17H23O3NK		0.033		
328.15434	C19H22O4N			-0.284	
329.00581	C14H10O7K				-0.691
329.13248	C26H17	0.077			0.013
329.13610	C14H26O6K		0.288		
329.18774	C19H30O2K		0.412		
329.19361	C12H34O7K		-0.107		
330.14030	C26H18	-0.168	-0.098		
331.11536	C13H24O7K		0.261		
331.13062	C17H24O4K		0.370		
331.14813	C26H19				0.181
331.15175	C14H28O6K		0.243		
331.17288	C11H32O8K		-0.168		
331.20911	C15H32O6Na		0.264	-0.036	
332.11957	C25H16O		0.004		
332.15595	C26H20	-0.224	-0.610		
333.05824	C11H18O9K		0.268		
333.08569	C12H21O8Ca		-1.668		
333.10988	C16H22O5K		0.301		
333.13101	C13H26O7K		0.187		
333.14627	C17H26O4K		0.298		
333.16740	C14H30O6K		0.040		
333.18265	C18H30O3K		0.295		
334.13522	C25H18O		-0.266		
334.17160	C26H22	-0.236			
335.08900	C18H16O5Na	0.208			
335.08915	C15H20O6K		0.224		
335.12538	C19H20O4Na		0.678		
335.16192	C17H28O4K		0.230		
335.19830	C18H32O3K		0.227		
335.21928	C18H32O4Na		0.286		
335.99945	C28	-0.294	0.203	-0.443	-0.220
336.12304	C20H18O4N				-0.121
336.18725	C26H24	-0.217			
337.10118	C27H13	-0.029	0.617		-0.150
337.10480	C15H22O6K		0.139		
337.14118	C16H26O5K		0.166		

337.17757	C17H30O4K		0.151		
337.21395	C18H34O3K		0.174		
338.01510	C28H2		0.142		
338.13869	C20H20O4N			-0.169	
339.06293	C17H16O5K	-0.035			
339.11683	C27H15	0.206	1.155		0.133
339.12045	C15H24O6K		0.489	-0.003	
339.15683	C16H28O5K		0.527		
339.19322	C17H32O4K		0.503		
339.22960	C18H36O3K		0.494		
340.12063	C22H16O2N2				-0.612
340.12465	C27H16	-0.067	-0.671		
340.13095	C18H23O3NK		0.117		
340.15434	C20H22O4N			0.001	0.185
341.07843	C20H14O4Na	0.348			
341.12845	C22H17O2N2				-0.477
341.13248	C27H17	0.189			0.212
341.17248	C16H30O5K		0.424		
341.81774	O9N3K4		0.459		
342.10392	C26H14O		-0.019		
342.14030	C27H18	-0.059	-0.490		
343.07453	C10H17O12N		-3.359	2.531	
343.09423	C17H20O5K		0.497		
343.14813	C27H19				0.330
343.15175	C15H28O6K		0.409		
344.08906	C18H16O7		1.582		
344.15595	C27H20	-0.005			
345.05824	C12H18O9K		0.453		
345.13101	C14H26O7K		0.412		
345.16740	C15H30O6K		0.337		
345.18853	C12H34O8K		-0.051		
346.13522	C26H18O		-0.329		
346.17160	C27H22	-0.045			
347.07389	C12H20O9K		0.344		
347.09487	C12H20O10Na		0.344	-0.178	
347.10540	C12H27O6Ca2	-0.623			
347.14666	C14H28O7K		0.306	-0.212	
347.16192	C18H28O4K		0.398		
347.18305	C15H32O6K		0.214	-0.254	
347.99945	C29		0.377		
348.09335	C28H12	-0.127	-0.269		
348.18725	C27H24	-0.193			
349.00728	C29H	0.170	0.425	-0.255	
349.03188	C17H10O7Na		0.809		
349.10480	C16H22O6K		0.310		
349.12593	C13H26O8K		0.356		
349.14103	C20H22O4Na		0.759		

349.16231	C14H30O7K		0.161		
349.17757	C18H30O4K		0.324		
350.10900	C28H14	-0.159	0.012	-0.356	-0.123
351.06293	C18H16O5K	-0.056			
351.11683	C28H15		0.823		
351.12045	C16H24O6K		0.222		
351.15683	C17H28O5K		0.268		
351.19322	C18H32O4K		0.262		
351.22960	C19H36O3K		0.276		
352.12465	C28H16	-0.184	-0.466		
353.04220	C17H14O6K		0.298		
353.09956	C18H18O6Na		0.634		
353.13248	C28H17	0.032			
353.13610	C16H26O6K		0.190		
353.17248	C17H30O5K		0.204		
353.20887	C18H34O4K		0.218		
354.14030	C28H18	-0.171	-1.155		
355.11521	C18H20O6Na		0.588		
355.15175	C16H28O6K		0.191		
356.15595	C28H20	-0.116	-1.292		
357.05237	C20H14O4K	0.086			
357.09448	C17H18O7Na		0.563		
358.17160	C28H22	-0.124			
359.14666	C15H28O7K		0.097		
359.20418	C13H36O8K		-0.332		
359.99945	C30	-0.106	0.103	-0.429	-0.131
360.18725	C28H24	-0.113			
361.05316	C12H18O10K		0.116		
361.05947	C16H17O7Ca	-0.524			
361.10118	C29H13	-0.037			-0.038
361.14118	C18H26O5K		0.130		
361.16231	C15H30O7K		0.033		
361.21395	C20H34O3K		0.079		
362.01510	C30H2		0.077		
363.06881	C12H20O10K	-0.037	0.063	-0.273	-0.180
363.08979	C12H20O11Na			-0.347	
363.11683	C29H15	-0.057	0.291		
363.14158	C14H28O8K		0.032	-0.250	
363.15668	C21H24O4Na		0.462		
363.17796	C15H32O7K		-0.147		
364.12465	C29H16	-0.243	-0.970		
365.10544	C12H22O11Na	0.519	0.334	-0.015	0.061
365.13248	C29H17				0.167
365.13610	C17H26O6K		0.372		
365.15723	C14H30O8K		0.410		
365.17248	C18H30O5K		0.410		
365.20887	C19H34O4K		0.388		

365.24525	C20H38O3K		0.412		
366.14030	C29H18	-0.087	-1.074		
366.96170	C14H9O7K2		-0.594		
367.07883	C18H16O7Na	0.556	0.789		
367.11521	C19H20O6Na		0.754		
367.15175	C17H28O6K		0.345		
367.18813	C18H32O5K		0.348		
367.22452	C19H36O4K		0.353		
368.14925	C21H22O5N			-0.047	0.196
368.15595	C29H20	0.016			
369.20378	C18H34O5K		0.283		
370.16490	C21H24O5N			-0.037	0.239
370.17160	C29H22	0.099			
371.99945	C31	0.125	0.282	-0.285	0.031
372.09335	C30H12				0.061
372.18725	C29H24	0.097			
373.00728	C31H	0.248	0.348	-0.042	
373.10480	C18H22O6K		0.273		
373.16231	C16H30O7K		0.240		
373.21983	C14H38O8K		-0.231		
373.23508	C18H38O5K		0.173		
374.10900	C30H14	-0.008	-0.046	-0.330	-0.034
375.14158	C15H28O8K		0.298		
375.15683	C19H28O5K		0.260		
375.17796	C16H32O7K		0.164		
375.19322	C20H32O4K		0.244		
376.12465	C30H16	-0.031	-0.147	-0.176	
377.19361	C16H34O7K		-0.041		
377.20887	C20H34O4K		0.201		
378.14030	C30H18	-0.087	-0.905		
379.06372	C12H20O11K		0.176	-0.216	
379.13062	C21H24O4K	0.545	0.287		1.068
379.15175	C18H28O6K		0.147		
379.18813	C19H32O5K		0.158		
379.22452	C20H36O4K		0.168		
379.26090	C21H40O3K		0.160		
380.15595	C30H20	-0.115			
381.07937	C12H22O11K	-0.012	0.133	-0.131	-0.011
381.09448	C19H18O7Na		0.511		
381.13086	C20H22O6Na		0.516		
381.16740	C18H30O6K			-0.062	
381.24017	C20H38O4K		0.128		
382.17160	C30H22	-0.008			
383.05276	C18H16O7K	0.224	0.149		
383.11028	C16H24O8K		0.094		
383.18305	C18H32O6K		0.101		
383.99945	C32	0.048	0.112	-0.350	-0.015

387.11683	C31H15	-0.020	0.008		
387.12045	C19H24O6K		0.075		
388.83050	O12N4CaK2Na		-0.028		
389.09527	C15H19O11N			1.843	
389.15723	C16H30O8K		-0.003		
389.17248	C20H30O5K		0.038		
389.19361	C17H34O7K		-0.093		
390.14030	C31H18	-0.126	-0.948		
391.17288	C16H32O8K		-0.046		
391.18798	C23H28O4Na		0.371		
391.20926	C17H36O7K		-0.281		
391.28429	C24H39O4		0.058	-0.255	
392.14684	C21H23O5NNa			0.049	0.242
392.15595	C31H20	-0.158			
393.05824	C16H18O9K			-0.510	
393.15214	C15H30O9K		-0.343		
393.24017	C21H38O4K		-0.064		
393.27655	C22H42O3K		-0.064		
395.21943	C20H36O5K		0.301		
395.25582	C21H40O4K		0.301		
397.00728	C33H	0.348			
397.12593	C17H26O8K		0.275		
397.23508	C20H38O5K		0.217		
398.10900	C32H14	0.069	-0.221		0.231
399.77700	H2O3N5K6Na2		-1.337		
400.12465	C32H16	0.079	-0.109	-0.045	
401.19361	C18H34O7K		0.160		
402.14030	C32H18		-0.474		
403.11092	C16H21O11N			1.877	
403.17288	C17H32O8K		0.157		
403.20926	C18H36O7K		0.117		
403.22452	C22H36O4K		0.187		
404.80444	O12N4CaK3		0.160		
405.07937	C14H22O11K		0.144		
405.08458	C23H14O4N2Na	1.197		0.200	
405.16740	C20H30O6K		0.176		
405.18853	C17H34O8K		0.060		
405.20363	C24H30O4Na		0.529		
405.22491	C18H38O7K		-0.009	0.105	
405.31294	C24H46O2K		0.158		
407.16779	C16H32O9K		-0.063		
407.29220	C23H44O3K		0.136		
407.99945	C34	0.244	0.107	-0.136	
408.12078	C21H23O5NK			-0.081	0.304
409.16216	C22H26O6Na		0.456		
409.18344	C16H34O9K		-0.224		
409.27147	C22H42O4K	0.138	0.086		

410.82139	O12N4K3Na2		0.073		
411.10519	C17H24O9K		-0.074		
413.13610	C21H26O6K		0.101		
413.26623	C24H38O4Na	0.101	0.050	-0.098	0.346
415.11536	C20H24O7K		0.047		
417.12657	C17H23O11N			1.667	
417.18853	C18H34O8K		-0.116		
419.20418	C18H36O8K		-0.172		
419.21928	C25H32O4Na		0.298		
419.99945	C35	0.200			
421.00728	C35H		-0.012		
421.05852	C23H14O4N2K	0.601	0.002		
421.21983	C18H38O8K		-0.302		
421.23508	C22H38O5K		-0.067		
421.30785	C24H46O3K		-0.088		
423.21435	C21H36O6K		-0.130	-0.150	
423.28712	C23H44O4K		-0.100		
424.12465	C34H16	0.019	-0.375		
425.13610	C22H26O6K		-0.078		
425.19346	C23H30O6Na		0.197		
426.14030	C34H18	-0.105	-0.875		
426.79533	O12N4K4Na		0.226		
427.11174	C33H15O		-0.550		
427.22452	C24H36O4K		0.225		
429.24017	C24H38O4K	0.335	0.219	0.016	0.464
431.20418	C19H36O8K		0.082		
431.99945	C36	0.359			
433.21983	C19H38O8K		0.024		
435.23548	C19H40O8K		-0.229		
435.32350	C25H48O3K		0.134		
437.20710	C25H29O5N2		-1.381		
437.30277	C24H46O4K		0.108		
441.16740	C23H30O6K		0.013		
441.18838	C23H30O7Na		0.400		
441.29753	C26H42O4Na		0.040		
442.76927	O12N4K5		0.023		
443.21943	C24H36O5K		0.010		
444.09335	C36H12				0.217
447.23548	C20H40O8K		-0.249		
447.25058	C27H36O4Na		0.315		
448.12465	C36H16		-0.264		
449.23000	C23H38O6K		-0.067		
449.33915	C26H50O3K		-0.065		
450.14030	C36H18		-0.289		
451.07898	C22H20O8K		-0.044		
451.31842	C25H48O4K		-0.076		
457.27147	C26H42O4K		-0.173		

461.25113	C21H42O8K	-0.383	
461.41192	C29H58OK	-0.177	
463.24565	C24H40O6K	-0.286	
463.31842	C26H48O4K	-0.243	
463.35480	C27H52O3K	-0.235	
463.39119	C28H56O2K	-0.224	
465.26130	C24H42O6K	-1.057	
465.33407	C26H50O4K	-1.018	
469.14706	C20H30O10K	-1.303	
471.25073	C26H40O5K	-1.174	
471.28712	C27H44O4K	-1.104	
472.12465	C38H16	-1.292	
474.14030	C38H18	-1.280	
477.37045	C28H54O3K	-1.150	
479.27695	C25H44O6K	-1.242	
479.34972	C27H52O4K	-1.210	
483.16271	C21H32O10K	-1.318	
485.30277	C28H46O4K	-1.285	
489.44322	C31H62OK	-1.350	
491.16764	C26H28O8Na		0.547
491.27695	C26H44O6K	-1.428	
491.42249	C30H60O2K	-1.330	
493.25606	C28H38O6Na	-1.136	
493.36537	C28H54O4K	-1.353	
495.32350	C30H48O3K	-1.428	
495.79898	O15N5K3Na3	-1.418	
498.14030	C40H18	-1.343	
503.42249	C31H60O2K	-1.472	
505.40175	C30H58O3K	-1.514	
505.43814	C31H62O2K	-1.522	
507.14158	C26H28O8K		1.618
507.27186	C26H44O7K	-2.330	
509.28751	C26H46O7K	-2.391	
509.32347	C28H41O3N6	-1.453	
511.77292	O15N5K4Na2	-2.289	
514.21733	C26H35O9Na	-1.205	
517.47452	C33H66OK	-2.401	
519.41740	C31H60O3K	-2.413	
519.45379	C32H64O2K	-2.384	
523.30316	C27H48O7K	-2.475	
527.74686	O15N5K5Na	-2.472	
531.45379	C33H64O2K	-2.546	
533.39667	C31H58O4K	-2.634	
537.28243	C27H46O8K	-2.661	
537.35477	C30H45O3N6	-1.797	
543.72079	O15N5K6	-2.617	
547.44870	C33H64O3K	-2.777	

551.29808	C28H48O8K		-2.736
553.31373	C28H50O8K		-2.839
567.32923	C32H48O7Na		0.170
595.32429	C30H52O9K		-0.337
615.09045	C16H26O18N5K	-0.661	

Table 2: List for all assigned sum formulae in negative-ion mode.

Theoretical mass M^-	Assigned sum formula	Mass error for each filter / ppm			
		FPI17B0807	FPI17B0811	287	289
59.01385	C2H3O2	-0.187	-0.133	-0.499	-0.398
59.98529	CO3	-0.272	-0.088	-0.459	-0.493
59.98828	HN2P	-0.922	-2.204	-1.291	
60.99312	CHO3	-0.206	0.020	-0.234	-0.468
61.98837	O3N	-0.021	0.102	-0.300	-0.274
62.96414	O2P	-0.119		-0.119	
62.98523	C2ONa			2.363	2.355
63.96245	O2S	-0.028	0.242	-0.177	-0.187
65.00329	C4HO	0.015	0.013	-0.090	-0.197
65.99854	C3ON	0.288	0.283	0.146	0.114
66.00977	C2N3	0.123		-0.018	-0.172
68.01419	C3H2ON		-0.020	-0.101	
68.99580	CH2O2Na	-0.540	-0.527	-0.557	-0.579
68.99820	C3HO2		0.024	-0.122	-0.202
69.03459	C4H5O		0.113		-0.197
71.01385	C3H3O2	-0.075	-0.099	-0.078	-0.232
71.05024	C4H7O	-0.071	-0.027	-0.141	-0.227
72.99312	C2HO3			-0.098	
73.00837	C6H	-0.061	-0.052	-0.136	-0.236
73.02950	C3H5O2	0.023	-0.051	0.004	-0.041
74.00362	C5N	-0.127	0.000	-0.197	-0.202
74.99381	H3O3Mg	-0.088			
75.00877	C2H3O3		-0.046	-0.257	-0.278
76.97005	HO3Si	-0.255		-0.259	-0.269
77.00329	C5HO		0.000	-0.111	-0.109
78.91888	Br	-0.059	-0.007	-0.058	-0.094
78.95906	O3P	0.083	-0.108	-0.153	-0.204
79.93961	OS2	0.079			
79.95737	O3S	0.080	-0.200	0.025	0.036
80.96519	HO3S	-0.097		-0.221	-0.300
83.05024	C5H7O		-0.043	-0.080	-0.117
84.00055	C7				-0.069
85.02950	C4H5O2	-0.149	-0.098	-0.133	-0.218
87.00877	C3H3O3	0.134	-0.157	0.235	0.194
87.04515	C4H7O2	0.136	-0.054	0.239	0.185
89.00329	C6HO	0.276	0.105	0.344	0.032
89.02442	C3H5O3	0.052	0.060	0.133	0.067
89.99854	C5ON	-0.147	-0.076	-0.122	-0.155
90.01008	H3N4P	-3.513		-3.365	-3.387
90.97136	H3O3Ca	-0.068			
91.02231	C3H7OS			-2.555	-2.634
92.92802	Cl2Na	-0.005	0.050	0.147	0.168
92.96519	CHO3S	-2.457		-2.366	-2.380

92.99580	C3H2O2Na			-0.126	
92.99820	C5HO2	0.094	0.071	0.133	0.150
93.00121	H4O3NAl		1.598	1.606	1.515
93.03459	C6H5O	-0.013	-0.069	0.340	0.141
94.02984	C5H4ON		0.094	0.164	0.146
94.91998	O2Cu				0.314
94.98061	H3O4Si	0.684	1.537		1.991
94.98084	CH3O3S			-0.003	
94.99305	H4O4Al	0.074		0.261	0.264
95.01385	C5H3O2		0.158	0.200	0.139
95.95228	O4S	0.192	0.084	0.939	0.869
95.97609	H2O3NS	0.141	0.104	0.920	0.797
96.00055	C8	0.110		0.213	0.152
96.94235	HO2S2			0.056	
96.96011	HO4S	0.628	0.432	0.697	0.729
96.96962	H2O4P	0.463	0.226	-0.395	-0.157
97.00837	C8H	0.608	0.215	0.577	0.556
97.02950	C5H5O2	0.186	0.122	0.177	0.103
97.97185	CH4Al2Si			0.490	0.499
98.00362	C7N	0.247	0.231	0.248	0.154
98.02475	C4H4O2N		0.170	0.158	
99.00877	C4H3O3	-0.016	0.154	0.218	0.082
99.04515	C5H7O2		0.154	0.231	0.124
99.92580	O3Cr	0.174		0.210	0.218
101.00329	C7HO		0.221	0.333	0.276
101.02442	C4H5O3	0.580	0.537	0.631	0.594
101.06080	C5H9O2	0.442	0.537	0.525	0.506
102.92334	O3Mn	0.215		0.249	0.233
102.97571	H4N2NaTi	0.090			
103.04007	C4H7O3	0.421	0.164	0.510	0.256
103.92023	O3Fe	0.326	0.426	0.465	0.505
103.95201	CONKNa	0.836			
103.99038	C6O2	0.183		0.220	0.219
104.92806	HO3Fe	0.435	0.232	0.362	0.276
104.98039	H3ON2AlP	0.845		0.890	
105.95845	H2O4Ca	0.323			
105.96044	CO3NS	0.201		0.234	0.231
106.91197	O2As	0.240		0.260	0.211
106.98084	C2H3O3S				0.241
107.05024	C7H7O		0.150	0.344	0.205
108.00055	C9	0.466	0.305	0.415	0.407
108.02168	C6H4O2	0.420	0.342	0.407	0.390
108.03157	C3H2N5			0.621	
108.93747	H2O2CaCl	0.290			
108.99071	C3H2O3Na	0.116		0.174	-0.100
109.00837	C9H	0.217		0.240	0.230
109.02950	C6H5O2	0.218	0.144	0.333	0.240

110.01620	C9H2				0.229
110.02475	C5H4O2N		0.358	0.231	
110.97576	CH3O4S			0.335	0.324
111.00877	C5H3O3	0.219	0.136	0.310	0.217
111.02000	C4H3O2N2		0.153		0.208
111.04515	C6H7O2	0.237	0.135	0.312	0.200
111.94720	O5S	0.165		0.223	0.186
111.99546	C8O				0.213
112.01659	C5H4O3	-1.415			
112.01927	C8H2N			0.290	
112.95502	HO5S			0.327	
112.98563	C2H2O4Na	-0.030	-0.202	0.002	-0.047
113.00329	C8HO	0.378	0.205	0.162	0.316
113.02442	C5H5O3	0.342	0.187	0.340	0.299
113.06080	C6H9O2		0.141	0.316	0.202
113.89392	OClCu	0.252		0.276	0.247
113.99854	C7ON	0.252	0.172	0.309	0.214
114.00981	H4N5Ca	-0.133			
114.01967	C4H4O3N	0.182	0.171		
114.03492	C8H4N	0.261		0.284	
114.90174	HOClCu	0.059		0.255	0.207
114.92622	O3ClS	0.137		0.292	0.243
114.93630	C2N2Cu	0.303	0.132	0.336	0.704
114.95163	H3O4Ti	0.242		0.232	0.165
115.00368	C4H3O4			0.279	0.201
115.01894	C8H3O	0.399		0.340	0.245
115.03017	C7H3N2	0.555		0.419	
115.04007	C5H7O3	0.112	0.129	0.228	0.184
115.07645	C6H11O2	-0.009	0.128	0.152	0.150
115.92072	O4Cr	0.050		0.192	
115.96661	CH2O3NCa	0.136			
116.02542	C6H2N3	0.128		0.206	0.222
116.05057	C8H6N			0.234	
116.92854	HO4Cr	0.024		0.179	0.043
116.99820	C7HO2		0.099	0.158	0.139
117.01933	C4H5O4	5.835	0.065	0.185	1.062
117.02067	C5HN4			0.133	
117.03459	C8H5O	0.084	0.056	0.143	0.131
117.04582	C7H5N2			0.357	
117.05572	C5H9O3	0.093	0.047	0.135	0.106
118.02984	C7H4ON	0.142	0.049	0.155	0.151
118.91825	O4Mn	0.072	0.061	0.144	0.159
118.94205	O4NaS	0.106		0.146	0.135
118.96627	CH3O4Ca	0.098			
119.01385	C7H3O2			0.166	0.144
119.02509	C6H3ON2		0.067	0.158	0.170
119.05024	C8H7O	0.065	0.049	0.143	0.128

119.91515	O4Fe	0.070	0.070	0.139	0.161
119.94699	O3NClNa	0.021	0.036	0.157	0.120
119.96152	H2O4NCa	0.037			
120.00055	C10	0.063	0.118	0.110	0.096
120.00910	C6H2O2N		0.143	0.136	0.138
120.04549	C7H6ON			-0.271	
120.92297	HO4Fe	-0.047		0.440	0.411
120.94554	HO5Ca	0.019			
121.00837	C10H	0.069	0.043	0.139	0.098
121.02950	C7H5O2	-0.080	-0.007	0.065	0.041
121.89728	O2ClMn	0.009		0.127	
122.00362	C9N	0.042	0.118	0.124	0.099
122.02475	C6H4O2N		0.019	0.076	0.092
122.03733	C7H6O2		-0.023	0.036	
122.89417	O2ClFe	-0.058	-0.011	0.040	0.042
122.90683	C2HClCu	0.300		0.390	0.432
122.97576	C2H3O4S			0.052	0.051
123.00877	C6H3O3	-0.204	-0.013	0.013	0.027
123.04515	C7H7O2	-0.033	-0.014	0.055	0.077
123.90207	CNClCu	-0.173	-0.051	0.002	0.003
123.92456	HO3CaCl	0.013			
123.94645	H4O4Fe	-0.076	0.012	0.012	0.052
124.01659	C6H4O3	-0.124	-0.005	0.016	0.037
124.99141	C2H5O4S			0.041	0.053
125.00329	C9HO	-0.037	0.035	0.050	0.030
125.02442	C6H5O3	-0.061	-0.022	0.003	0.046
125.06080	C7H9O2		-0.007	0.045	0.047
125.09719	C8H13O		0.000		
125.99345	C3H3O4Na			-0.068	
126.01967	C5H4O3N				0.062
126.90359	HOCaCl2	0.580			
126.90502	I	-0.089	-0.107	-0.054	-0.073
126.97613	CO4N2Na	-0.057	0.018		
127.00128	C3H4O4Na			-0.113	
127.00368	C5H3O4		-0.022	0.045	0.046
127.04007	C6H7O3	-0.167	-0.063	-0.079	-0.079
127.07645	C7H11O2	-0.206	-0.071	-0.085	-0.055
127.99038	C8O2			0.045	0.023
128.03532	C5H6O3N		-0.055		
128.89215	Cl3Mg	-0.117		0.026	0.083
128.98045	C8HS	-1.155		0.038	
129.01933	C5H5O4		-0.040	0.063	0.054
129.05572	C6H9O3	-0.147	-0.142	-0.036	-0.077
129.09210	C7H13O2	-0.185	-0.150	-0.073	-0.092
129.88883	O2ClCu	-0.111		0.019	0.028
129.92343	H4O2KMn			-0.934	
129.93872	H2O5Ti	-0.149		-0.055	

129.97569	C7NS	0.566			
130.93121	C2ON2Cu	-0.159	-0.063	0.007	0.097
130.95370	CHO4NCa	-0.174			
130.97104	O5N2Na	-0.220	-0.056	-0.007	-0.284
130.99219	O6N2Li	-0.159	-0.087	0.025	
131.03498	C5H7O4		-0.043	-0.088	-0.001
131.03766	C8H5ON			0.050	
131.06147	C8H7N2			-2.772	
131.07137	C6H11O3	-0.273	-0.104	-0.162	-0.069
132.00055	C11	-0.206	-0.088	-0.119	-0.116
132.02168	C8H4O2	-0.433		0.034	
132.04549	C8H6ON			0.020	0.021
132.86785	Cl2Cu	-0.231	-0.199	-0.056	-0.087
132.91762	CHN2CaCr	0.717		0.955	
132.96011	C3HO4S			-0.021	0.012
132.98192	C2H5O4Ca	-0.095			
133.00837	C11H	-0.162	-0.075	-0.027	0.005
133.01425	C4H5O5			0.041	0.080
133.01693	C7H3O2N			-0.011	
133.02950	C8H5O2	-0.125	-0.068	-0.003	0.005
133.93272	CHO2NCaCl	-0.127			
134.01620	C11H2	-0.097		0.019	0.026
134.02475	C7H4O2N	-0.164	-0.084	-0.085	0.004
134.04722	C5H4N5	-0.097			
134.89466	O3ClCr	-0.234		-0.061	-0.021
134.91599	O4KS	0.307			-0.095
134.94861	HO5NCa	-0.100			
134.97576	C3H3O4S			-0.368	-0.442
135.02402	C11H3	-0.107	-0.107	0.012	0.010
135.02990	C4H7O5			3.456	
135.04515	C8H7O2	-0.292	-0.160	-0.173	-0.175
135.92093	O3NClK	-0.176		0.250	0.044
135.92099	HO5Mn		-0.562		
135.93263	O6Ca	-0.044			
135.95644	H2O5NCa	-0.161			
135.99546	C10O		0.010	0.025	-0.021
136.01659	C7H4O3	-0.492	-0.094	-0.239	
136.01927	C10H2N	-0.110		0.004	0.024
136.04040	C7H6O2N		-0.101	0.005	0.016
136.05298	C8H8O2			-0.039	
136.93239	CH2O3CaCl	-0.171			
136.94000	O3NAlMg2	0.377		0.409	0.466
136.99141	C3H5O4S			-0.129	-0.044
137.00329	C10HO	-0.178	-0.138	-0.099	-0.161
137.01452	C9HN2	-0.119		0.003	
137.02442	C7H5O3	-0.309	-0.190	-0.171	-0.182
137.03565	C6H5O2N2	-0.272	-0.110	-0.200	-0.065

137.06080	C8H9O2		-0.089	-0.002	
137.89219	O3ClMn			0.048	
137.92764	HO3NCaCl	-0.151			
137.94495	CHO3NKMg	0.168	0.347	0.399	0.418
137.99586	C6H2O4			-0.433	-0.234
137.99854	C9ON	-0.093	-0.038	0.017	0.020
138.01967	C6H4O3N	-0.267	-0.212	-0.171	-0.190
138.03224	C7H6O3		-0.140		
138.88909	O3ClFe	-0.204	-0.088	0.033	0.010
138.91165	O4CaCl	-0.132			
138.93630	C4N2Cu			1.899	
138.93739	C2ON2KS				-1.876
138.95268	CH3O4SSi	-1.787		-1.677	-1.674
138.97067	C2H3O5S			-0.043	-0.025
139.00368	C6H3O4	-0.174	-0.105	-0.056	0.011
139.00706	C3H7O4S			-0.120	-0.032
139.01492	C5H3O3N2		-0.076	0.031	0.004
139.01894	C10H3O	-0.117	-0.091	-0.048	-0.010
139.03021	C3H7N4Ca	-0.369			
139.04007	C7H7O3		-0.106	-0.047	-0.032
139.07645	C8H11O2		-0.070		
139.89691	HO3ClFe	0.288	0.404		0.515
139.89699	CONClCu			-0.082	
139.91614	CONClKMg	0.366			
139.96592	CH2O5NS			-1.572	-1.592
139.97850	C2H4O5S			-2.536	
140.01419	C9H2ON			0.095	
140.02542	C8H2N3			0.024	0.031
140.02546	C2H6N5Ca	-0.398			
140.87121	OCl2Mn	-0.073		0.130	
140.93195	HO5SSi			-0.017	-0.022
140.98632	C2H5O5S			-0.036	-0.028
140.99820	C9HO2		-0.008	0.043	0.014
141.01933	C6H5O4	1.715	-0.122	0.271	0.171
141.03459	C10H5O		-0.059	0.080	0.022
141.04586	C3H9N4Ca	-0.355			
141.05572	C7H9O3		-0.088	-0.054	-0.027
141.09210	C8H13O2	-0.327	-0.209	-0.180	-0.218
141.86811	OCl2Fe	-0.096	-0.127	-0.105	-0.151
141.89068	O2CaCl2	-0.160		0.087	
141.91256	H3O3ClFe	-0.181		-0.004	0.004
141.94518	O6NS	-0.166	-0.143	-0.136	-0.186
142.02984	C9H4ON	-0.152	-0.096	0.022	0.013
142.87593	HOCl2Fe	-0.190		0.022	-0.012
143.01385	C9H3O2			0.153	0.046
143.02509	C8H3ON2	-0.196	-0.097	0.000	0.011
143.03498	C6H7O4		0.182	0.336	-0.003

143.05024	C10H7O	-0.035	-0.091	0.141	0.032
143.07137	C7H11O3	-0.189	-0.231	0.009	-0.177
143.10775	C8H15O2	-0.230	-0.253	-0.137	-0.177
143.89190	O2NClCu	0.469		0.605	0.431
144.00055	C12	-0.260	-0.070	-0.113	-0.165
144.04549	C9H6ON	-0.156	-0.099	-0.049	-0.005
144.86970	CaCl3	-0.248		-0.043	-0.016
144.87164	H2CaKZn	-0.642			
145.00837	C12H	-0.213	-0.162	-0.161	-0.194
145.02950	C9H5O2	-0.296	-0.128	-0.195	-0.083
145.05063	C6H9O4	-0.344	-0.142	-0.346	-0.103
145.08702	C7H13O3	-0.240	-0.122	-0.186	-0.041
146.00362	C11N	-0.091		-0.024	-0.016
146.02475	C8H4O2N	-0.304	-0.129	-0.202	-0.126
146.90683	C4HClCu	-0.135		0.032	-0.006
146.92613	C2O2N2Cu			0.047	
146.96596	O6N2Na	-0.257	-0.285	-0.156	-0.162
147.00877	C8H3O3			-0.120	-0.073
147.02402	C12H3	-0.202			
147.02990	C5H7O5			-0.072	-0.542
147.04515	C9H7O2		-0.137	-0.146	-0.031
147.05639	C8H7ON2			-0.084	-0.018
147.90207	C3NClCu	-0.198	-0.156	-0.037	-0.015
147.96901	C2H4O5Ca	4.020			
148.04040	C8H6O2N			-0.079	-0.020
148.91981	CO3NCaCl	-0.145			
148.94170	CH3O4NFe	-0.393			
148.96906	HO6N2Mg	4.702			
148.99141	C4H5O4S	-1.648		-1.142	-0.989
149.00329	C11HO		0.056	0.067	0.005
149.00957	C3H9O3Si2	-0.373	-0.125	-0.208	
149.00959	C2H8O4NK				-0.217
149.02442	C8H5O3	-0.366	-0.153	-0.268	-0.150
149.06080	C9H9O2		-0.140	-0.186	-0.042
150.00191	C7H4ONS	-0.160			
150.01111	C11H2O	-0.226		-0.221	-0.151
150.01967	C7H4O3N		-0.100	-0.020	0.003
150.03224	C8H6O3			-0.580	
150.87014	HOCl2Zn			-0.047	-0.008
150.90174	C3HOClCu		-0.026	0.034	0.085
150.92104	CO3N2Cu		-0.093	-0.045	-0.014
150.94353	HO6NCa	-0.128			
150.94804	C2H4O3CaCl	-0.181			
151.00368	C7H3O4	-0.108	-0.002	0.031	0.014
151.00706	C4H7O4S			-0.936	-0.384
151.02749	C7H5O3N			-3.021	
151.04007	C8H7O3	-0.366	-0.201	-0.239	-0.218

151.87627	CNCl2Fe	-0.150		0.005	
151.89883	CONCaCl2	-0.176			
151.99038	C10O2				0.038
152.01151	C7H4O4	-0.353	-0.234	-0.253	-0.238
152.03532	C7H6O3N	-0.340	-0.202	-0.265	-0.186
152.91473	O4NCaCl	-0.170			
152.91761	HO5CaS	-0.098		-0.132	-0.011
152.93661	H3O5NFe	-0.151			
152.97110	C5H2N2KMg	0.104			
152.98054	C4H2O5Na	-0.758			
152.98632	C3H5O5S			-0.188	-0.069
153.01933	C7H5O4	-0.333	-0.280	-0.134	-0.206
153.02271	C4H9O4S			-0.186	-0.088
153.05572	C8H9O3		-0.131	-0.035	-0.029
153.09210	C9H13O2		-0.112		
153.92255	HO4NCaCl	-0.288	-0.047	-0.030	0.000
154.00603	C10H2O2		-0.059	0.034	0.020
154.01458	C6H4O4N		-0.234	-0.141	-0.187
154.89802	CNClKNaS	2.804			
154.90657	O5CaCl			-2.540	-2.507
154.91873	HO4ClNaS			0.010	
154.92836	C2H2O2ClKNa	-0.560		-0.390	
154.94737	H3O6Si2	-0.230			
154.94760	CH3O5SSi		-1.627	-1.654	-1.660
154.95952	C2H3O2N2CaSi	1.532			
154.95975	C3H3ON2CaS			0.393	0.405
154.96559	C2H3O6S			-0.033	-0.014
154.97233	C4H5ONCaS	-0.753			
154.99860	C6H3O5				0.018
155.00197	C3H7O5S			-0.367	-0.059
155.03498	C7H7O4			0.034	0.006
155.07137	C8H11O3		-0.100	-0.145	-0.013
155.10775	C9H15O2	-0.326	-0.146	-0.241	-0.123
155.89375	O2NCaCl2	-0.141			
155.91112	O3NCl2Mg	-0.218	-0.111	-0.139	-0.017
156.00055	C13	-0.269	-0.125	-0.186	-0.183
156.03291	C9H4ON2	0.033		0.014	
156.86613	O2Cl2Mn			0.382	
156.92686	HO6SSi	-2.697		-0.183	-0.184
156.93618	H4O4NAs	1.164		1.296	
156.93930	H2O6AlS			0.035	0.071
156.96011	C5HO4S			0.099	
156.96325	CH5O5SSi	-1.524		-1.494	-1.483
156.99649	C6H5O3S	-0.243		-0.116	-0.011
157.00837	C13H	-0.173	-0.018	-0.084	-0.043
157.01425	C6H5O5			0.006	0.027
157.04074	C9H5ON2	-0.014			

157.05063	C7H9O4		-0.038	0.026	0.053
157.08702	C8H13O3	-0.147	-0.223	-0.094	-0.163
157.12340	C9H17O2	-0.173	-0.249	-0.137	-0.163
157.86302	O2Cl2Fe	-0.276	-0.086	-0.159	-0.027
158.00362	C12N	-0.009			
158.01620	C13H2	-0.161	-0.127	-0.071	-0.013
158.02475	C9H4O2N			-0.027	
158.03733	C10H6O2	-1.287	-0.760	-1.754	
158.92523	H2O4NCaK	0.807			
158.92797	C2O2N2CaCl	0.379			
158.94251	H3O6SSi			-0.225	-0.198
158.95183	H6O4NAs	1.090			
159.00877	C9H3O3			0.004	
159.01145	C12HN	0.002			
159.02402	C13H3	-0.168	-0.096	-0.115	-0.008
159.02990	C6H7O5		-0.083	-0.021	-0.033
159.03258	C9H5O2N	-0.407		-0.052	
159.04515	C10H7O2		-0.128	-0.077	-0.014
159.06628	C7H11O4		-0.147	-0.038	-0.014
159.10267	C8H15O3		-0.116	-0.087	-0.032
159.84515	Cl3Mn	-0.169		-0.053	0.032
159.87047	CNCl2Zn			-0.102	-0.005
159.87867	H2O2Cl2Fe	-0.263	-0.100	-0.177	-0.143
159.88682	O3NClCu	-0.288	-0.200	-0.233	-0.193
159.99546	C12O		-0.058		-0.023
160.01927	C12H2N	-0.131		-0.059	-0.010
160.04040	C9H6O2N	-0.149	-0.116	-0.071	0.003
160.84205	Cl3Fe	0.067	-0.199	0.177	-0.094
161.00329	C12HO	-0.262	-0.115	-0.141	-0.142
161.00916	C5H5O6			-0.029	0.001
161.01452	C11HN2	-0.144		-0.004	
161.02442	C9H5O3	-0.293	-0.134	-0.239	-0.092
161.03565	C8H5O2N2		-0.078	-0.022	0.001
161.04555	C6H9O5		-0.085	-0.071	
161.06080	C10H9O2		-0.110	-0.027	0.001
161.99854	C11ON	-0.027		0.026	
162.01967	C8H4O3N	-0.218	-0.103	-0.177	-0.050
162.03492	C12H4N	-0.070		0.021	
162.92378	H3O5Ca2	-5.546			
162.93989	O6N2K	-0.120	-0.212	-0.191	-0.181
163.00368	C8H3O4	-0.322	-0.214	-0.152	-0.162
163.01492	C7H3O3N2		-0.103		
163.01894	C12H3O	-0.119	-0.048	-0.078	0.010
163.04007	C9H7O3	-0.279	-0.135	-0.218	-0.100
163.07645	C10H11O2	-0.303	-0.129	-0.180	-0.050
163.89653	H6O2Cu2		-0.339		
163.90822	C2N3ClCu			0.016	

163.93645	C4H5FeNaS				1.435
163.93676	C2O4NKNa	-0.486	-0.126	-0.618	
163.95135	CH2O6NCa	-0.101			
164.01419	C11H2ON			0.032	
164.02542	C10H2N3	0.076			0.478
164.03532	C8H6O3N		-0.098	-0.047	0.021
164.93495	O6NNaS			-0.002	0.001
164.94660	HO6N2Ca	-0.271			
164.96397	HO7N2Mg	-0.332	-0.127	-0.056	-0.005
164.99820	C11HO2		-0.030	0.012	0.050
165.01933	C8H5O4	0.129	-0.182	-0.084	-0.114
165.03057	C7H5O3N2			-0.193	
165.04046	C5H9O6	2.135		2.237	2.286
165.04089	C3H12O4NK		-0.255		
165.05572	C9H9O3		-0.092	-0.046	-0.004
165.85767	H3OClFe2				-2.600
165.93989	O5N2ClNa			-0.165	
165.95241	C2H2O4NKNa	-0.234			
165.95711	C3O3N3Ca	-0.577			
166.01458	C7H4O4N	-0.486	-0.085	-0.210	-0.217
166.02716	C8H6O4	-0.065			
166.02984	C11H4ON			0.055	
166.04107	C10H4N3	-2.094			
166.05097	C8H8O3N		-0.092	-0.010	0.025
166.89716	CO2N2CaMn			-2.994	
166.91780	O4N2CaCl	-0.575			
166.93517	O5N2ClMg	-0.227		0.231	
166.99860	C7H3O5		-0.073	0.054	0.071
167.00197	C4H7O5S			-0.054	0.011
167.01385	C11H3O2			0.078	0.077
167.02106	C5H3O3N4	1.396		3.264	-0.348
167.02241	C7H5O4N				-0.713
167.02509	C10H3ON2			0.061	
167.03498	C8H7O4	0.103	-0.073	0.115	0.071
167.03836	C5H11O4S			-0.202	
167.07137	C9H11O3		-0.068		0.060
167.83671	Cl3Cu			0.018	0.074
167.87118	CONCl2Fe	-0.179	-0.076	0.049	0.122
168.00055	C14	-0.047	0.011	0.071	0.045
168.00642	C7H4O5	-0.065	-0.061	-0.042	0.040
168.03023	C7H6O4N		-0.097	-0.088	0.016
168.04549	C11H6ON			0.102	
168.83625	Cl3Zn	-0.148	-0.182	0.006	-0.034
168.88994	C2H3O2Mn2	0.278		0.067	0.150
168.90964	O5NCaCl	-0.172	-0.011	-0.057	
168.91415	C2H3O2CaCl2	0.083			
168.94865	C5H2N2CaK	0.196			

168.98124	C3H5O6S			-0.001	
169.00837	C14H	-0.171	-0.049	-0.095	-0.092
169.01425	C7H5O5	0.409	-0.090	0.048	0.062
169.01762	C4H9O5S			0.036	0.056
169.02950	C11H5O2		-0.067	0.066	0.080
169.04074	C10H5ON2	-0.076		0.072	
169.05063	C8H9O4				0.104
169.06589	C12H9O		-0.044		
169.08702	C9H13O3	-0.218	-0.074	-0.145	-0.120
169.12340	C10H17O2	-0.265	-0.086	-0.144	-0.055
169.89487	C2HO2ClMnNa			0.226	
170.00362	C13N	0.024			
170.01620	C14H2			0.089	0.120
170.02475	C10H4O2N	-0.058	-0.037	0.048	0.096
170.88372	O4CaClS	0.088		-0.039	
170.90683	C6HClCu	-0.614		-0.003	0.018
170.93691	C3HO2N2ClK	1.978			
170.94228	H3O7Si2	-0.093		0.021	0.094
170.99716	C4H6O3NNaS				-1.560
171.01145	C13HN	0.346			
171.01214	C7H7O3S			-0.058	-0.022
171.02000	C9H3O2N2	-0.145			
171.02402	C14H3	0.036			
171.02990	C7H7O5			0.106	
171.04515	C11H7O2		-0.037	0.025	0.043
171.06628	C8H11O4		-0.025	0.037	0.066
171.10267	C9H15O3	-0.115	-0.131	-0.078	-0.103
171.13905	C10H19O2	-0.074	-0.161	-0.083	-0.102
171.88866	O3NCaCl2	-0.040	-0.080	-0.079	0.011
171.90207	C5NClCu	-0.028		0.043	
172.02783	C9H4O2N2	-0.923			
172.04040	C10H6O2N		-0.030	-0.004	0.019
172.95793	H5O7Si2	-0.258		-0.215	-0.156
172.97028	C9HO2S	0.060		-0.497	
172.99141	C6H5O4S			-0.185	
173.00329	C13HO			0.001	0.035
173.00916	C6H5O6			-0.294	
173.02442	C10H5O3	-0.131	-0.041	-0.079	0.024
173.04555	C7H9O5			-0.084	
173.06080	C11H9O2		-0.047	-0.078	-0.005
173.08193	C8H13O4		-0.059	-0.077	0.007
173.11832	C9H17O3		-0.025	-0.076	
173.96576	H6O7Si2			-3.150	
173.98946	C3H4O6NMg		0.041	-0.166	
174.00191	C9H4ONS	0.411	0.495	0.478	
174.01111	C13H2O	-0.187	-0.017	-0.176	-0.069
174.01967	C9H4O3N		-0.023	-0.027	0.046

174.87679	O5CuS			-0.105	
174.90174	C5HOClCu			-0.350	0.096
174.93743	H3O7SSi	-0.111		-0.200	-0.144
174.94706	C2H4O5KSi	-0.780			-0.664
175.02481	C6H7O6			-0.089	-0.012
175.02749	C9H5O3N			-0.089	
175.04007	C10H7O3		-0.051	-0.151	0.000
175.88462	HO5CuS			-0.189	-0.002
175.93337	C5H4NClCu				-2.650
176.03532	C9H6O3N		-0.021	-0.064	0.045
176.81734	BrClCu			0.025	
176.83696	OCl3Fe	-0.029	-0.011	-0.087	0.003
176.88417	HO5SZn			-0.108	-0.019
176.91692	HO7S2			-0.135	-0.064
176.92655	C2H2O5KS				-0.561
176.92931	H2O6NaSi2	-0.029			
176.98045	C12HS	0.153			
177.01933	C9H5O4	-0.203	-0.059	-0.143	-0.068
177.05572	C10H9O3	-0.005	-0.026	-0.091	0.039
177.09210	C11H13O2		-0.032	-0.039	0.040
177.84479	HOCl3Fe	-0.112		-0.007	0.076
178.00603	C12H2O2		0.044	0.016	0.078
178.01458	C8H4O4N		-0.001	-0.029	0.061
178.05097	C9H8O3N			-0.011	
178.09993	C11H14O2		0.003	-0.020	0.045
178.91780	CO4N2CaCl	0.036			
178.92025	C3HO4NZn			0.701	
178.99860	C8H3O5		0.017	-0.028	0.067
179.01385	C12H3O2				0.089
179.03498	C9H7O4	-0.036	-0.006	-0.055	0.039
179.03836	C6H11O4S			-0.150	
179.05611	C6H11O6		-0.084		
179.07137	C10H11O3		0.016		
179.86030	O2NCl2Zn			-0.043	0.054
179.88262	O6CrS			-0.876	
179.92649	C3H2O3KNaS	0.660			
179.93369	O7N2Ca	-2.347		-3.075	-3.491
179.95844	CH5O5N2Mn		-0.131	-0.256	-0.117
180.00055	C15	-0.012	0.068	-0.010	-0.006
180.03023	C8H6O4N		0.012	0.024	0.089
180.04549	C12H6ON			0.019	
180.06662	C9H10O3N		0.023		
180.94152	HO7N2Ca	0.039	-0.029	-0.044	0.022
180.95358	C6HON2S2	0.548		0.459	0.602
181.00837	C15H	0.023	0.064	0.025	0.050
181.01425	C8H5O5	0.078	0.014	-0.052	0.056
181.01762	C5H9O5S			-0.097	0.012

181.05063	C9H9O4	-0.032	0.019	0.048	0.106
181.89778	CH2O5FeS	-1.224		1.155	-1.111
181.91747	CHO5NCaCl	-0.097			
181.92514	O7NMgS	0.008	0.017	-0.004	0.000
181.93760	C6O2NS2	0.530	0.539	0.568	0.528
181.94712	C2H3O6AlS	0.712	0.457	0.519	0.753
181.99174	C7H4O3NS	-0.211			
182.00950	C7H4O5N		0.049	0.042	0.116
182.01620	C15H2	0.009	0.049	0.076	0.144
182.04588	C8H8O4N		0.043	0.022	0.089
182.90554	H4O4Ca2Cl	-0.531			
182.91272	O5N2CaCl	0.059	0.150		
182.93008	O6N2ClMg	0.076	-0.025	-0.019	-0.010
182.93716	H6N3CaClSSi	-0.028			
182.99689	C4H7O6S			-0.077	
183.00877	C11H3O3			0.087	0.138
183.01214	C8H7O3S			-0.169	-0.102
183.01732	C7H5O5N			-0.175	
183.02402	C15H3	-0.038	0.039	0.006	0.111
183.02990	C8H7O5	-0.126	0.039		
183.03327	C5H11O5S			-0.229	
183.06628	C9H11O4				0.111
183.10267	C10H15O3	-0.065	0.033	-0.095	0.063
183.13905	C11H19O2	-0.147	0.016	-0.061	0.080
183.87703	C2H2O3Mn2	0.262		0.166	0.213
183.91343	CH4O5FeS	-0.504		1.374	-0.880
183.99546	C14O				0.133
184.01927	C14H2N	0.095		0.078	0.155
184.02515	C7H6O5N			0.100	
184.04040	C11H6O2N		0.063	0.068	0.133
184.87668	CH3OCa2ClK	-0.554			
184.89451	C2H2O4FeK	-0.775			
184.92126	CH5O5FeS	-1.094			-1.025
184.99141	C7H5O4S			0.019	0.084
185.00329	C14HO	-0.066	0.081	-0.018	0.046
185.00916	C7H5O6				0.246
185.01452	C13HN2	0.064			
185.02442	C11H5O3		0.070	0.074	0.155
185.03565	C10H5O2N2			0.053	
185.03967	C15H5	0.135			
185.04555	C8H9O5		0.119		
185.06080	C12H9O2		0.053		
185.08193	C9H13O4		0.064	0.017	0.128
185.11832	C10H17O3	-0.146	0.042	-0.031	0.091
185.15470	C11H21O2	0.060	-0.007	0.035	0.027
185.85911	HNCaClS3			0.425	0.541
185.89268	C2H4O3Mn2	0.162		0.114	0.353

185.90233	C2H3O4FeK	-0.537			
185.99854	C13ON	0.190			
186.01967	C10H4O3N	0.066		0.086	0.166
186.03492	C14H4N	0.136			
186.85607	O4ClFeS	0.035		0.088	0.079
186.86412	O3NCl2Mn	0.421	0.087	0.029	0.148
186.87017	H2N2Cl2MnS			-0.088	-1.334
186.88944	CO3N2ClZn			0.062	0.165
186.89764	H2O5NClFe	-0.061	0.124	-0.061	0.095
186.90579	O6N2Cu	-0.060	0.071	-0.082	0.052
186.91482	C4HN2CaCl2	-0.098		-1.125	
187.00706	C7H7O4S			0.002	0.043
187.01894	C14H3O	0.128	0.144	0.013	0.155
187.02481	C7H7O6				0.107
187.04007	C11H7O3	0.064	0.079	0.078	0.155
187.06120	C8H11O5		0.042	0.089	0.166
187.09758	C9H15O4		0.057	0.069	0.151
187.13397	C10H19O3		0.030		
187.86101	O3NCl2Fe	0.129	0.068	0.116	0.095
188.01419	C13H2ON			0.143	
188.03532	C10H6O3N	0.056	0.092	0.074	0.167
188.86713	O2N2ClKMn			-0.004	0.049
188.95308	CH5O7SSi			-1.118	-0.998
188.99820	C13HO2			0.186	0.209
189.01933	C10H5O4	0.101	0.111	0.081	0.183
189.03057	C9H5O3N2			0.102	
189.03459	C14H5O	0.175			
189.05572	C11H9O3		0.094	0.124	0.188
189.94185	CO6N3Ca	0.092			
189.95922	CO7N3Mg	-0.029	0.093		
190.01458	C9H4O4N		0.129	0.119	0.204
190.05097	C10H8O3N		0.123	0.100	0.168
190.91828	H2O5NCaNaS	2.295			
190.99860	C9H3O5		0.116	0.089	0.194
191.01385	C13H3O2	0.269		0.153	0.210
191.01973	C6H7O7			0.053	0.184
191.03498	C10H7O4	0.096	0.110	0.054	0.184
191.05611	C7H11O6				-0.470
191.07137	C11H11O3		0.131		
192.00055	C16	0.135	0.196		0.331
192.03023	C9H6O4N		0.128	0.155	0.227
192.04549	C13H6ON			0.223	
192.87929	CH3O3ClCuS			2.438	1.615
192.92853	CH3O3N3CaTi	2.600			
192.95886	O8N3Na	0.174	0.303	0.409	
193.00837	C16H	0.097	0.152	0.136	0.181
193.01425	C9H5O5	0.123	0.131	0.162	0.227

193.02950	C13H5O2		0.157	0.235	0.269
193.04074	C12H5ON2	0.201		0.168	
193.05063	C10H9O4	0.196	0.146	0.147	0.248
193.05401	C7H13O4S			0.163	
193.06589	C14H9O		0.151		
193.85073	O4ClCuS			0.138	0.190
193.86065	H2O5Fe2	0.073		-0.161	
193.86626	HO3NClSZn				0.283
193.93677	O7N3Ca	0.053			
193.95414	O8N3Mg	0.079	0.130	0.172	0.191
194.00362	C15N	0.306			
194.01620	C16H2	0.281	0.191	0.226	0.326
194.04320	C6H10O7		0.180		
194.04588	C9H8O4N		0.154	0.201	0.285
194.82402	O3ClFe2	0.328			
194.85028	O4ClSZn	0.359		0.258	0.243
194.85567	O3NCl2Cu	0.149	0.144	-0.178	0.012
194.85856	HO4ClCuS			-0.122	0.043
194.88934	C4H2NClSZn			4.215	
194.88941	H3O6Ti2	3.777			
194.90686	C2H5N2CaClCu	-0.821		-0.140	-0.147
194.92749	H3O8S2			0.112	0.167
194.99689	C5H7O6S			0.165	0.224
195.02402	C16H3	0.314			0.327
195.02990	C9H7O5	0.012	0.157	0.248	0.301
195.03327	C6H11O5S			0.079	0.188
195.04515	C13H7O2	0.155	0.137	0.208	0.281
195.06628	C10H11O4	0.181			
195.13905	C12H19O2		0.187	0.190	
195.81090	Cl4Fe	0.453	0.178	0.361	0.289
195.85522	O3NCl2Zn	0.198	0.168	0.209	0.218
195.90211	CH4N3CaClCu			0.022	
195.92861	O8N2Ca	0.214	0.182	0.145	0.265
195.93312	C2H3O5NCaCl	0.066			
195.95325	C7H2O2NS2			0.549	0.612
196.02515	C8H6O5N			0.220	0.271
196.03185	C16H4	0.317			
196.04040	C12H6O2N			0.179	
197.00329	C15HO				0.226
197.00916	C8H5O6				0.216
197.01254	C5H9O6S			0.099	0.216
197.02442	C12H5O3			0.252	
197.02779	C9H9O3S			-0.443	0.247
197.04892	C6H13O5S			0.040	
197.06080	C13H9O2		0.204	0.243	
197.08193	C10H13O4		0.198		
197.15470	C12H21O2		0.213	0.109	0.208

197.90269	O7NCaS	0.240	0.209	0.094	0.196
197.91163	HO7NKS		0.244	0.089	0.181
198.00441	C7H4O6N			0.213	
198.01111	C15H2O	0.317	0.253	0.128	0.208
198.04080	C8H8O5N			0.083	
198.89887	O7NaS2			0.187	
198.90763	O6N2CaCl	0.344	0.217	0.072	0.163
198.91921	H3O7Si3	-0.560			
199.00706	C8H7O4S			0.000	0.124
199.01894	C15H3O			0.231	
199.04007	C12H7O3		0.195	0.167	0.240
199.09758	C10H15O4		0.189	0.113	
199.13397	C11H19O3		0.219	0.180	0.236
199.17035	C12H23O2	0.301	0.204	0.186	0.176
199.85106	O6Cr2	-0.068			
199.88351	C2H2ONCa2S2	0.472			
199.99038	C14O2			0.158	0.236
200.02676	C15H4O	0.308			
200.03532	C11H6O3N			0.060	0.181
200.87166	H3O3Ca2Cl2	-0.303			
200.93486	H5O7Si3	-0.504			
200.98632	C7H5O5S			-0.002	0.118
201.01933	C11H5O4		0.007	0.072	0.191
201.05572	C12H9O3		0.001	0.092	0.210
201.11323	C10H17O4		0.053		
201.87955	CH3O4ClFeS	-1.105			
202.00603	C14H2O2		0.048	0.056	0.188
202.01458	C10H4O4N	0.175		0.046	
202.05097	C11H8O3N		-0.037		
202.84004	CONCl3Fe	0.203			
202.85099	O5ClFeS			0.025	0.169
202.88680	C6H2ClKMn	1.642		1.691	1.814
203.01385	C14H3O2		0.611	0.238	0.165
203.03498	C11H7O4		-0.005	0.056	0.170
203.07137	C12H11O3		0.023		
203.85881	HO5ClFeS			0.006	0.113
203.86897	C3N3Cu2	-3.019			
204.00055	C17	0.127	0.023	0.027	0.100
204.03023	C10H6O4N	0.141	-0.008	0.056	0.153
204.11558	C13H16O2			0.018	
204.86375	HO4NCl2Fe	0.239		-0.378	0.034
204.92459	C9HS3			-0.006	
205.00837	C17H	-0.022	-0.013	-0.025	0.070
205.01425	C10H5O5		0.001	0.063	0.153
205.05063	C11H9O4	0.226	-0.020	0.048	0.153
205.15979	C14H21O		-0.043		
205.93667	H6O9Fe		0.416		

205.93677	CO7N3Ca	0.021			
206.00950	C9H4O5N		-0.029	0.050	0.139
206.01620	C17H2	0.092	0.068	-0.008	0.105
206.04588	C10H8O4N		-0.025	0.031	0.129
206.87927	O5N2ClZn				0.206
206.90973	CH3O6S3			1.266	1.341
206.99351	C9H3O6		-0.091	0.015	0.107
207.00877	C13H3O3				0.064
207.01145	C16HN	0.352		0.353	
207.02402	C17H3	0.130	0.019	0.020	0.145
207.02990	C10H7O5	0.289	-0.015	0.020	0.121
207.06628	C11H11O4		-0.045		
207.06966	C8H15O4S			-0.009	
207.99546	C16O				0.144
208.01927	C16H2N	0.278		0.087	
208.02515	C9H6O5N				0.119
208.93643	CHO8N2Ca	0.145	0.005	0.134	0.071
209.00329	C16HO	0.110	-0.021	0.019	0.070
209.00916	C9H5O6		-0.074	-0.067	0.056
209.01254	C6H9O6S			-0.143	-0.016
209.03565	C12H5O2N2			0.024	
209.03967	C17H5	0.306			
209.04555	C10H9O5		-0.070	0.029	0.084
209.04892	C7H13O5S			-0.114	-0.007
209.06080	C14H9O2		-0.042		
209.85557	H2O6Fe2	0.304		0.138	0.077
209.93168	O8N3Ca	0.812	0.131		0.181
209.94905	O9N3Mg	0.145	-0.065	0.072	0.080
209.96552	C11O2NS			2.578	
210.03492	C16H4N	-0.056			
210.86339	H3O6Fe2	0.138			
210.95291	C8H3O3S2		1.194	1.117	
210.99180	C5H7O7S			-0.068	0.000
211.01894	C16H3O	-0.025	-0.083	-0.063	0.019
211.02819	C6H11O6S			0.018	-0.100
211.04007	C13H7O3	0.141	-0.079	-0.006	0.066
211.07645	C14H11O2	0.098			
211.08769	C13H11ON2			0.028	0.089
211.13397	C12H19O3		-0.048	0.000	0.050
211.17035	C13H23O2		-0.092	-0.042	
211.79237	OCl2Cu2			-0.053	0.066
211.87122	H4O6Fe2	0.023			
212.01419	C15H2ON			-0.027	
212.03532	C12H6O3N		-0.056	0.002	0.072
212.78574	BrCl2Zn			-0.012	0.045
212.83475	C2N2ClCu2			-0.058	
212.87904	H5O6Fe2	-0.044		-3.177	-2.944

212.98632	C8H5O5S			-0.253	
212.99820	C15HO2			-0.028	0.024
213.01933	C12H5O4	0.123	-0.093	-0.009	0.056
213.02271	C9H9O4S			-0.117	-0.019
213.03057	C11H5O3N2			-0.107	
213.03459	C16H5O	-0.018		-0.051	
213.05572	C13H9O3		-0.137		
213.14962	C12H21O3		-0.059	-0.012	
213.18600	C13H25O2	-0.047	-0.112	-0.077	-0.039
213.84241	H3O4ClFe2	-0.157			
213.87504	O7NFeS			-0.007	0.010
213.88308	O6N2ClMn	-0.021	-0.098	-0.091	-0.014
213.88902	C7OClFeNa			0.559	0.561
213.91661	H2O8N2Fe	-0.110	-0.099	-0.118	0.000
214.02716	C12H6O4				-4.365
214.05097	C12H8O3N		-0.164	-0.055	
214.87998	O6N2ClFe	0.095	-0.060	-0.039	0.000
214.91413	H3O8Si3	0.049		-0.062	-0.056
214.92656	H4O8AlSi2	0.346			
214.93007	C7H3O2S3			0.344	
215.00197	C8H7O5S			-0.139	-0.075
215.01385	C15H3O2	0.248	0.039	-0.018	0.004
215.03498	C12H7O4	0.187	-0.106	-0.083	0.003
215.83809	CO2NBrCaK	0.657			
215.91326	H2O8NCaS			-0.157	-0.074
215.92532	C6H2O2NS3			0.352	
216.03023	C11H6O4N			-0.086	0.000
216.04281	C12H8O4			-2.086	-2.482
216.04549	C15H6ON			-0.072	
216.80981	HAlCl2MnS2			0.272	
216.88039	O6N2ClNi			-0.225	
216.90943	H2O8NaS2	1.351		-0.081	-0.021
216.92978	H5O8Si3	-0.115		-0.109	-0.058
216.99312	C14HO3			0.441	
217.00837	C18H	-0.014	-0.115	-0.066	-0.031
217.01425	C11H5O5			-0.052	0.006
217.02950	C15H5O2			-0.070	
217.04074	C14H5ON2	0.248			
217.05063	C12H9O4	-0.176	-0.185	-0.107	-0.059
217.06589	C16H9O	0.050		-0.070	
218.01620	C18H2	-0.168		-0.145	-0.084
218.04588	C11H8O4N				-0.006
218.81047	CdCl3			-0.026	0.058
218.83777	H2O2Ca2Cl3	-0.232			
218.88576	HO6N2KMn			-1.076	-1.016
218.90686	C4H5N2CaClCu	0.681			
219.02402	C18H3	0.137			

219.02990	C11H7O5		-0.177	-0.119	-0.062
219.04515	C15H7O2			-0.050	
219.06628	C12H11O4		-0.132		
219.17544	C15H23O		-0.134	-0.093	
219.84566	CH2O3Cl2FeS	-0.728		-1.059	-1.026
220.02515	C10H6O5N			-0.028	-0.035
220.03185	C18H4	0.645			
220.14688	C14H20O2	0.317	-0.057	0.061	0.005
220.85349	CH3O3Cl2FeS			-0.656	
220.86970	O7NCuS			-0.139	-0.071
221.00329	C17HO				0.001
221.00916	C10H5O6				-0.053
221.03967	C18H5	0.221			
221.04555	C11H9O5		-0.086	-0.064	-0.013
221.06080	C15H9O2		-0.082		
221.80053	CNCl2Cu2	-0.036	-0.165	-0.072	-0.052
221.82493	O4Cl2FeS	0.478		-0.067	-0.002
221.86924	O7NSZn			-0.057	-0.007
221.87464	O6N2ClCu	0.432	-0.059	-0.098	-0.025
221.87752	HO7NCuS			-0.219	
221.88294	CH2O6Ca2S			-0.007	0.110
222.01111	C17H2O	0.116	-0.102	-0.131	-0.079
222.82987	O3NCl3Fe	0.364	-0.007	-0.043	0.008
222.87418	O6N2ClZn	0.207	-0.080	0.048	0.008
222.88788	CH2O5NCa2Cl	0.454		-0.114	
222.95208	C2H3O8N2Ca	0.341	-0.010	-0.059	-0.041
223.02481	C10H7O6			-0.039	-0.024
223.02819	C7H11O6S		0.553	0.252	0.335
223.04007	C14H7O3	0.031	-0.146	-0.124	-0.069
223.06457	C8H15O5S			-0.182	-0.122
224.03532	C13H6O3N			-0.099	
225.00745	C6H9O7S			-0.164	-0.032
225.01933	C13H5O4		0.006	-0.039	-0.045
225.04046	C10H9O6		-0.164		
225.04384	C7H13O6S			-0.327	-0.290
225.05572	C14H9O3	0.190	0.072	-0.083	-0.054
225.18600	C14H25O2		-0.011	-0.138	
225.92660	O9N3Ca	0.068	-0.105	0.011	-0.037
226.00603	C16H2O2		-0.279	-0.173	-0.157
226.90249	C2O6N2CaK	-0.431			
226.99860	C12H3O5				-0.038
227.01385	C16H3O2			-0.182	-0.201
227.03498	C13H7O4	-0.511	-0.231	-0.151	-0.117
227.04622	C12H7O3N2			-0.172	
227.20165	C14H27O2	-0.101	-0.098	-0.055	-0.095
227.92284	C2O6N4Cr	-1.172			
228.00055	C19	-0.606	-0.327	-0.212	-0.177

228.03023	C12H6O4N		-0.191		-0.107
228.82966	C2ON2ClCu2	-0.604			
228.84769	CH3O3Cl2SZn			-0.289	-0.264
229.00837	C19H	-0.490	-0.221	-0.150	-0.137
229.01425	C12H5O5		-0.151	-0.115	-0.084
229.05063	C13H9O4	-0.066	-0.121	-0.092	-0.080
229.07176	C10H13O6			-0.161	-0.128
229.18092	C13H25O3			-0.115	-0.088
229.84294	H2O3NCl2SZn			-0.126	-0.087
229.94822	C4H8O3NCa2S	-0.479	-0.080	0.059	0.083
230.01620	C19H2	-0.109	-0.255	-0.200	-0.161
230.76631	Cl3Cu2			-0.100	-0.093
230.82696	HO4Cl2SZn			-0.168	-0.106
230.87489	O7N2ClFe			0.401	
230.90432	CH5O3NCaMg2S2	-1.606			
231.01145	C18HN	-0.376		-0.060	
231.02402	C19H3	-0.264	-0.179	-0.154	-0.123
231.02990	C12H7O5		-0.089	-0.115	-0.075
231.06628	C13H11O4	-0.450	-0.193		
231.17544	C16H23O		-0.173		-0.101
231.88272	HO7N2ClFe			-0.086	-0.095
231.94354	O9N3Na2	-0.218	-0.051	-0.115	-0.090
232.01927	C18H2N	0.040		-0.096	
232.88337	H2O8KS2			-0.148	-0.095
232.92469	H5O9Si3	-0.388		-0.151	-0.108
232.93736	C4H2O8NaS	-1.547		-1.696	
233.00329	C18HO	0.038	-0.059	-0.089	-0.078
233.03967	C19H5	0.089			
233.04555	C12H9O5		-0.124	-0.110	-0.090
233.06080	C16H9O2	-0.044			
233.15470	C15H21O2	-0.382	-0.138	-0.137	-0.116
233.91648	H3O2N6AsCa	-0.290	0.034	-0.041	
234.01111	C18H2O				-0.074
234.03492	C18H4N	-0.029			
234.81991	O6ClCr2			4.082	4.210
234.95208	C3H3O8N2Ca	-0.162	-0.083		
235.01894	C18H3O	-0.260	-0.135	-0.103	-0.090
235.02481	C11H7O6				-0.077
235.04007	C15H7O3	-0.077	-0.097		
235.06120	C12H11O5		-0.080		
235.95334	C8O5N2S			0.942	0.970
236.03532	C14H6O3N			-0.039	
236.04789	C15H8O3	-0.959			
236.88363	O4N4K3				0.232
236.91984	H5O9SSi2			-0.088	-0.059
236.93135	C2HO9N2Ca		-0.131		
236.98510	C3H5O9N2Mg	-0.412	-0.123	-0.179	-0.084

237.01933	C14H5O4		-0.111	-0.085	-0.117
237.03459	C18H5O	0.162			
237.04384	C8H13O6S			-0.254	-0.218
237.05572	C15H9O3	-0.234	-0.124		
237.08022	C9H17O5S			-0.134	
237.98437	C7H4O7NMg		0.150		
237.99665	C6H6O10		1.133		
239.01385	C17H3O2	-0.032	0.011	-0.058	-0.063
239.02310	C7H11O7S			-0.238	-0.213
239.03498	C14H7O4	0.061	-0.069	-0.070	-0.046
239.05611	C11H11O6			-0.074	-0.121
239.05949	C8H15O6S		0.584	0.257	0.373
239.07137	C15H11O3		-0.073		
239.12888	C13H19O4		-0.095	-0.063	
240.03023	C13H6O4N				-0.001
240.90205	O9N3Mn	-0.076	-0.020	-0.012	-0.012
241.00237	C6H9O8S		-0.079	-0.042	-0.028
241.00837	C20H	-0.042	-0.091	-0.133	-0.069
241.01425	C13H5O5				-0.027
241.05063	C14H9O4		-0.050	-0.020	-0.031
241.21730	C15H29O2	-0.045	0.014	-0.003	-0.022
241.80216	O3NCl2Mn2			0.443	
241.89894	O9N3Fe	-0.029	0.031	0.007	0.004
242.01620	C20H2	-0.061	-0.144	-0.126	-0.115
242.02207	C13H6O5				-0.689
242.85710	H5O6BrCu	0.096	0.074		
242.94152	CH3O9N2MgS		-0.120	-0.236	-0.127
243.01145	C19HN	0.142			
243.02402	C20H3	0.035			-0.073
243.02990	C13H7O5		-0.039	-0.007	-0.020
243.06628	C14H11O4		-0.064		
243.89935	O9N3Ni	0.389	-0.040	-0.004	0.025
243.93677	H2O9N3MgS			-0.068	-0.041
244.03185	C20H4	-0.077			
244.88779	H5O8FeSi2	0.129			
244.92079	HO10N2MgS			-0.069	-0.016
245.04555	C13H9O5		-0.043		0.006
245.06080	C17H9O2	-0.001	-0.031		
246.01111	C19H2O	-0.108	-0.251	-0.030	-0.034
246.96945	C4H3O9N2Mg		0.037		
247.01894	C19H3O				-0.020
247.02481	C12H7O6		0.012		
247.04007	C16H7O3	0.009			
247.06120	C13H11O5	0.228	0.016		
247.09758	C14H15O4	0.086			
247.17035	C16H23O2		-0.018		0.026
247.83895	O8FeS2			0.003	0.033

247.91748	O9N3KNa	0.036	0.012		
247.99038	C18O2		-0.009	0.064	0.026
248.02676	C19H4O	0.033			
248.07170	C16H10O2N			0.046	
248.81943	C2O2N2Cu2K			0.312	0.304
248.84389	O7NClFeS			0.028	0.043
248.89360	O9N3Cu		0.044	0.033	0.055
248.91961	H5O10Si3	-0.109		-0.018	0.008
249.04384	C9H13O6S				-0.349
249.05572	C16H9O3	-0.007	-0.001		
249.84883	O6N2Cl2Fe	-0.125	0.036	0.025	0.077
249.89315	O9N3Zn	0.015	0.076	0.130	0.098
249.90687	O10N3Ti	0.024	0.036	-0.029	-0.006
249.99692	C8H5O7NNa	0.328	0.075	1.197	0.127
250.00603	C18H2O2		0.051	0.054	0.035
250.14487	C14H20O3N		0.014	-0.006	0.025
250.15744	C15H22O3		0.054	-0.006	0.057
251.01385	C18H3O2				0.034
251.03096	C10H7O6N2			-0.239	
251.03498	C15H7O4	0.101			
251.05949	C9H15O6S			-0.055	-0.045
251.07137	C16H11O3	0.077			
252.00055	C21	0.041	-0.068	0.021	0.021
252.80890	O4Cl2NaSZn			0.097	
252.91475	H5O10SSi2			0.108	0.095
252.96265	C3H5O9N2Ca	0.038	0.124	0.006	0.056
253.00837	C21H	-0.080	0.013	0.027	0.049
253.03875	C8H13O7S			-0.106	-0.121
253.05063	C15H9O4	0.086	0.064		0.109
253.08702	C16H13O3		0.040		
253.21730	C16H29O2			0.042	0.051
253.81384	O3NCl3NaZn			0.107	0.102
254.01620	C21H2	-0.062	-0.045	0.018	0.029
255.01145	C20HN	0.230			
255.02402	C21H3	0.116	0.097	0.053	0.050
255.02990	C14H7O5		0.086	0.147	0.120
255.06628	C15H11O4	0.234	0.117		
255.23295	C16H31O2	0.334	0.214	0.271	0.181
255.84144	HO8CuS2			0.070	0.107
255.86560	H7O5Cl3Zn				4.203
255.98963	C13H6ONS2			0.340	
256.01927	C20H2N	0.208		0.118	
256.83232	H3N4Cl2Zn2				1.587
256.84926	H2O8CuS2			0.377	0.263
256.99728	C6H9O9S				0.257
257.00329	C20HO	0.193	0.128	0.129	0.129
257.03967	C21H5	0.244			

257.04555	C14H9O5	0.221	0.131		
257.78527	O3NCl2Cu2				0.201
257.81995	O5N2CaMn2			-2.534	-2.456
257.84630	O4B2Cl4S			-1.323	-1.319
257.89386	O10N3Fe			0.734	0.710
258.88597	H3O9Si4	1.146			
258.90168	HO10N3Fe		0.260	0.272	0.273
258.91907	CH3O9N2CaS	-0.429	0.136	0.183	0.107
259.01293	C6H11O9S			0.059	-0.015
259.01894	C20H3O	0.186	0.136	0.171	0.159
259.02481	C13H7O6		0.213	0.256	0.213
259.06120	C14H11O5	0.271			
259.87488	H4O9FeSi2	0.156			
259.91432	H2O9N3CaS	0.222	0.200	0.856	0.343
260.03532	C16H6O3N			0.229	
260.89833	HO10N2CaS	0.201	0.245	0.225	0.260
260.90162	H5O9Si4	-0.370			
261.01933	C16H5O4				0.120
261.03459	C20H5O	0.207			
261.04046	C13H9O6		0.248	0.352	0.281
261.05572	C17H9O3		0.236		
261.18600	C17H25O2		0.243		
261.89053	H6O9FeSi2	0.097			
262.94700	C4H3O9N2Ca		0.295		
263.01385	C19H3O2			0.275	0.239
263.03498	C16H7O4	0.289	0.257		
263.05611	C13H11O6		0.249		0.270
263.05949	C10H15O6S			0.014	
263.89142	O9N3K2		0.268		
264.16052	C15H22O3N		0.274		
265.00837	C22H	0.200	0.183	0.223	0.240
265.01425	C15H5O5		0.274		
265.02950	C19H5O2	0.453			
265.03875	C9H13O7S			0.066	
265.05063	C16H9O4	0.322			
265.14791	C12H25O4S		0.247		
265.97086	C8H5O7NK		0.323		
266.01218	C17H2O2N2			-0.768	
266.01620	C22H2	0.152	0.123	0.206	0.172
266.77738	H2O3Cl3Fe2	0.213		0.432	
267.02402	C22H3	0.257			0.213
267.05440	C9H15O7S			0.152	0.146
267.06628	C16H11O4	0.344	0.307		
268.03185	C22H4	0.332			
269.04555	C15H9O5	0.396	0.344		0.366
269.08193	C16H13O4	0.500	0.359		
269.21222	C16H29O3		0.369	0.374	

269.24860	C17H33O2	0.246	0.328	0.341	0.331
270.01111	C21H2O	0.396	0.267	0.347	0.339
271.02481	C14H7O6		0.378		0.406
271.06120	C15H11O5	0.515	0.393		
271.83797	H4O8Fe2Si	0.461			
272.82783	CH2O6ClS2Zn				-0.454
272.84580	H5O8Fe2Si	0.213			
272.84678	C2HO8FeS2				-5.475
273.04046	C14H9O6		0.430		
273.86963	O9N3Sr	0.284	0.355	0.589	
273.88359	CH2O9N2FeS			-0.316	-0.293
274.00603	C20H2O2	0.421	0.336	0.443	0.410
274.80710	O7ClS2Zn			0.498	0.448
274.88089	H3O10Si4	0.260		0.322	0.304
274.89332	H4O10AlSi3	0.439			
274.91398	CH3O10N2CaS				0.457
274.93257	C8H3O7S2			3.259	3.353
274.93336	C4H4O10CaNa		0.498		
275.01973	C13H7O7				0.495
275.86286	O10N2FeS			-0.234	-0.188
276.00055	C23	-0.308	-0.342	-0.265	-0.265
276.86780	O9N3ClFe	-0.378	-0.150	-0.228	-0.156
276.89654	H5O10Si4	0.034		-0.235	-0.184
277.00837	C23H	-0.232	-0.255	-0.249	-0.225
277.03538	C13H9O7				-0.063
277.06187	C16H9O3N2	-0.126			
278.01620	C23H2	-0.231	-0.301	-0.261	-0.250
278.82276	H3O4S4Si3			0.491	
278.97826	C11H3O9		-0.093		
279.02402	C23H3	-0.238	-0.236	-0.226	-0.232
279.05440	C10H15O7S			-0.290	-0.299
279.89239	H2O7N4K2S				0.352
280.01927	C22H2N	0.017		-0.067	
280.02702	C8H15O5NCaCl	1.178			
281.00329	C22HO	-0.129	-0.174	-0.126	-0.128
281.03967	C23H5	-0.161			
281.24860	C18H33O2			-0.075	
281.78855	CH2N2Cl3Fe2Na	1.366			
283.01894	C22H3O	-0.109	-0.127	-0.079	-0.094
283.02481	C15H7O6			-0.075	
283.06120	C16H11O5	0.018	-0.052		
283.26425	C18H35O2	-0.204	-0.003	-0.099	-0.047
283.80107	H4O7Fe3	0.036			
284.96741	H5O12N3Na2	1.001			
284.96769	C13HO8		0.160		
285.01933	C18H5O4				-0.131
285.03459	C22H5O	0.037			

285.04046	C15H9O6	0.076	0.002	0.053	
285.05572	C19H9O3		-0.005		
285.07685	C16H13O5	0.375	-0.009		
286.21496	C16H30O4			0.201	
287.01385	C21H3O2			0.043	0.053
287.01973	C14H7O7			0.273	0.105
287.05611	C15H11O6	0.123			
288.00055	C24			0.010	-0.016
288.89745	C5H5O6S4			-0.710	-0.757
289.00837	C24H	-0.024	-0.060	0.013	0.027
289.03538	C14H9O7		0.210	0.295	0.225
290.01620	C24H2	0.015		0.037	
291.02402	C24H3	0.014			
291.05103	C14H11O7		0.212		
291.16018	C17H23O4		0.167		
292.03185	C24H4	0.252			
292.06153	C17H10O4N			0.124	
292.81766	H2O8ClS2Zn			0.160	0.201
292.83775	H5O9Ti3	1.865			
292.89145	H5O11Si4	0.029		0.118	0.175
292.90350	H5O4N6FeSi3	1.337		1.982	
292.95752	C11HO10		0.316		
294.01111	C23H2O	0.213	0.133	0.224	0.199
295.07645	C21H11O2	-0.279			
295.86942	O8N4Ca2S			1.817	1.839
295.99038	C22O2			-0.329	-0.355
297.04046	C16H9O6		-0.343		
297.15299	C16H25O3S			-0.315	-0.341
297.24352	C18H33O3			-0.271	
298.00603	C22H2O2			-0.305	-0.327
298.98334	C14H3O8	-0.236			
299.05611	C16H11O6	-0.406	-0.315		
300.00055	C25		-0.389	-0.371	-0.376
300.98002	C7H5O10N2Mg		-0.219		
301.00837	C25H	-0.385	-0.361	-0.309	-0.305
301.07176	C16H13O6	-0.135	-0.248		
302.01620	C25H2	-0.447		-0.330	-0.331
303.02402	C25H3	-0.356	-0.369	-0.251	-0.280
303.88676	O12N4Fe	-0.370	-0.135	-0.352	-0.196
304.01927	C24H2N	-0.039			
304.70006	CaCl3SZn2			0.008	-0.029
305.00329	C24HO	-0.216	-0.236	-0.182	-0.170
305.03029	C14H9O8			0.344	0.070
305.03967	C25H5	-0.157			
306.20747	C18H28O3N		-0.150		
307.01894	C24H3O	-0.169	-0.179	-0.102	-0.123
307.04594	C14H11O8			0.186	0.008

308.80793	O6N2Ca2Cl3	-0.216			
309.03459	C24H5O	-0.014			
309.96294	C14O8N	0.021			
310.90202	H7O12Si4				-0.176
310.90266	H11O8SSi5			-1.850	
310.90418	O12N4CaNa	-0.289	-0.012	1.029	
311.16864	C17H27O3S			0.008	-0.003
312.88375	Cl3Pb	0.066	0.063	-0.227	0.278
312.98002	C8H5O10N2Mg	-0.006	0.031		
313.00837	C26H	0.001			-0.007
313.02950	C23H5O2			0.091	
313.07176	C17H13O6	-0.024	0.029	0.013	
314.01620	C26H2	-0.154		0.032	
314.02395	C14H7O8B			1.863	
315.02402	C26H3	-0.089			
315.03177	C14H8O8B			-3.657	
315.05103	C16H11O7		0.154		
316.03185	C26H4	-0.413			
316.92113	O12N4Na3	-0.707	-0.518	-0.425	-0.481
316.95756	C7H5O10N2Ca	-0.558	-0.432		
318.01111	C25H2O		-0.543	-0.408	-0.448
319.84893	O10N3CaK2	0.672			
322.00603	C24H2O2		-0.388	-0.321	-0.321
322.71947	H2ONCa2ClSr2				1.079
323.86924	O9N3Ba	-0.401	-0.278	-0.392	-0.303
324.00055	C27				-0.377
325.00837	C27H	-0.498	-0.398	-0.263	-0.294
325.07176	C18H13O6		-0.293		
325.18429	C18H29O3S			-0.196	-0.262
326.01620	C27H2	-0.463		-0.284	-0.361
326.87812	O12N4CaK		-0.219		
327.02402	C27H3	-0.374	-0.395	-0.282	-0.230
328.01927	C26H2N	-0.365		0.109	
328.71630	HNCaCl6Zn			2.262	2.386
328.95756	C8H5O10N2Ca	-0.300	-0.177	0.894	0.288
329.00329	C26HO		-0.253	-0.108	-0.118
329.03967	C27H5	0.009			
329.06668	C17H13O7		-0.125		
330.84181	H3O12CaS3			0.009	-0.028
331.00368	C22H3O4			-0.170	
331.01894	C26H3O	-0.205		-0.121	-0.070
332.89507	O12N4KNa2	-0.301	-0.020		
333.03459	C26H5O	0.035			
335.82689	O9N3Ca2Cl2	-0.314	0.016		
337.00837	C28H				0.082
338.01620	C28H2	-0.206			
338.87894	H7O12Si5	-0.489			

338.89777	O4ClPbS			-0.495	-0.464
339.19994	C19H31O3S			-0.470	
339.90271	O3NCl2Pb	-0.352	-0.489	-0.425	-0.457
340.03185	C28H4	-0.423			
342.01111	C27H2O				-0.426
345.86298	O12N3Mg2S2	0.498		0.627	0.825
346.00603	C26H2O2				-0.314
346.86831	O12N4CaClMg	-0.841	-0.363		
348.86901	O12N4K2Na		-0.327		
349.00837	C29H	-0.550	-0.400	-0.416	-0.288
350.01620	C29H2	-0.428			-0.332
351.02402	C29H3	-0.361	-0.392	-0.148	-0.180
353.00329	C28HO		-0.282		-0.128
353.03967	C29H5	0.003			
354.80771	HO9N3CaS2Zn			1.658	1.789
354.87386	H7O13Si5	-0.241		-0.110	-0.103
355.01894	C28H3O			-0.074	
357.06159	C18H13O8		-0.138	0.074	-0.006
361.84092	O13N3Ca2S	-0.393	0.005	1.007	0.054
362.84586	O12N4Ca2Cl	-0.215	0.025	0.157	0.121
366.92168	O6N2ClPb	-0.837	-0.485	-0.819	-0.408
370.86877	H7O14Si5	-1.126		-0.827	-0.379
373.00837	C31H	-0.615		-0.353	-0.316
373.88675	O10N8K2Mg	0.512			
373.88728	O15N5CaMg		-0.343	-0.496	-0.296
375.02402	C31H3	-0.415	-0.426	-0.056	
379.01894	C30H3O			0.057	
386.09402	C12H20O13N		0.021		
389.86483	O15N5Ca2	0.100	0.078	-0.368	0.100
393.94065	O9N3Pb		-0.413	-0.529	-0.275
395.88177	O15N5CaNa2	-1.305	-0.447		
397.00837	C33H	-0.674		-0.232	-0.306
399.02402	C33H3	-1.124		0.188	
401.89872	O15N5Na4	-0.933	-0.314		
402.08893	C12H20O14N			-0.564	
404.10458	C12H22O14N		-0.252	-0.284	-0.116
411.85571	O15N5CaKNa	-0.983	-0.110		
412.82497	H5O15Si6				-0.244
417.87266	O15N5KNa3	-0.802	0.024		
421.00837	C35H	-0.244		0.389	
427.82965	O15N5CaK2		-0.391		
430.83553	H7O16Si6			0.318	-0.085
433.84660	O15N5K2Na2		-0.261		
449.82054	O15N5K3Na		0.060		
458.86487	O18N6CaMgNa		0.270		
465.79448	O15N5K4		-0.267		
474.83971	O18N5KNa2S	2.921			

474.84241	O18N6Ca2Na	0.326	0.049
480.85936	O18N6CaNa3	0.579	0.115
490.81635	O18N6Ca2K	0.822	0.141
496.83330	O18N6CaKNa2	0.977	0.308
502.85025	O18N6KNa4		0.418
512.80724	O18N6CaK2Na		-0.091
518.82419	O18N6K2Na3		-0.002
528.78118	O18N6CaK3		0.139
534.79812	O18N6K3Na2		0.260
550.77206	O18N6K4Na		0.438
553.80253	O16N10Ca2K2		1.574
559.82124	H5O15N8CaK3Na2	2.929	
575.79341	O16N10CaK3Na	2.649	1.360
597.78430	O16N10K4Na2		1.600

4 Compounds significant for each city

Both cities could be separated by mean of an HCA clustering approach as shown in Figure 3 a (negatively charged ions) and b (positively charged ions) below. The dendrogram shows significant sum formulas for both cities from the complete dataset. The heatmap indicates the intensity of the respective sum formula, which is increased (red) or decreased (blue) significantly in the corresponding city. Different clusters are also shown in red and blue colors.

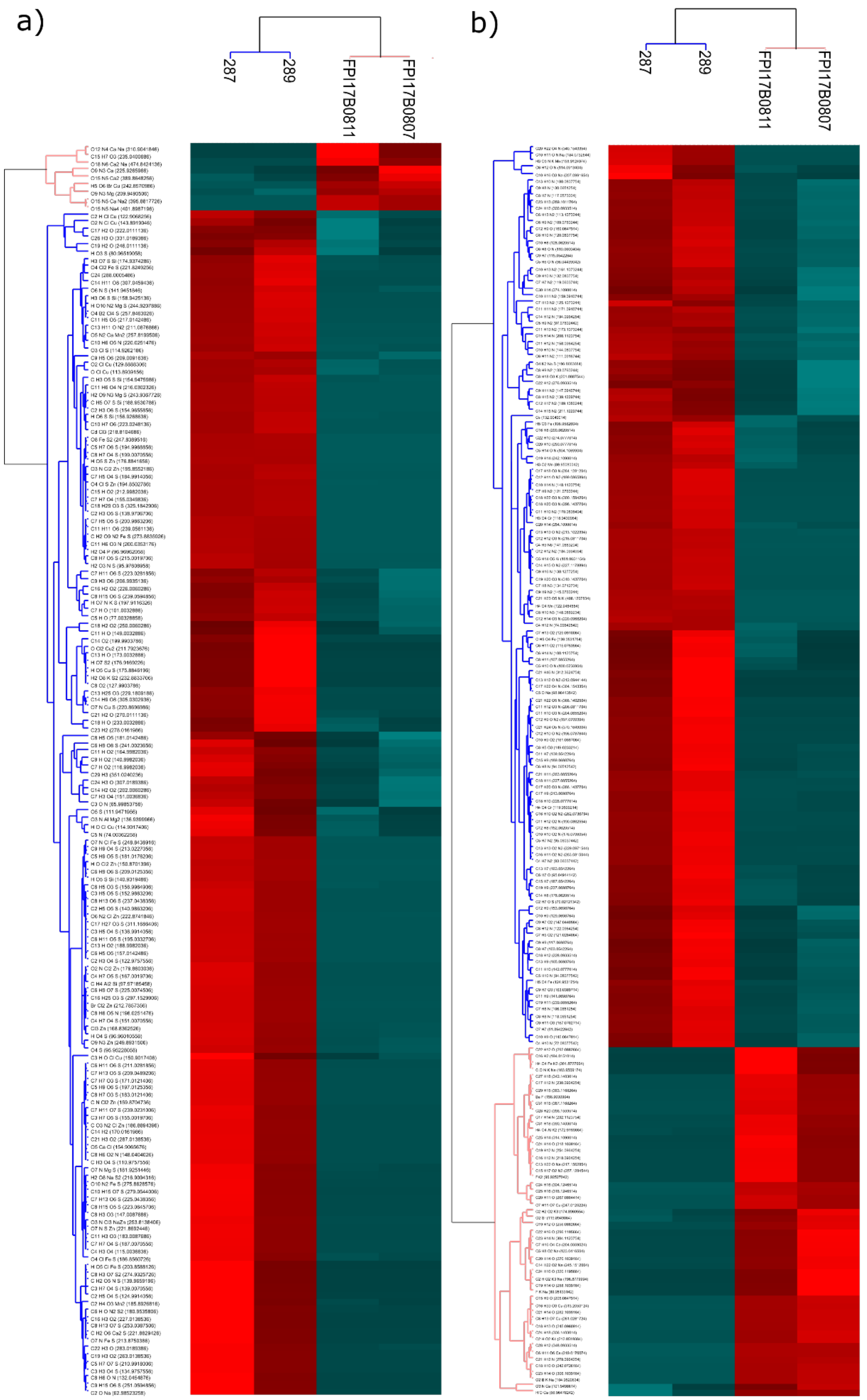

Figure 3: HCA of Teheran and Hangzhou samples for negative- (a) and positive-ion mode (b) measurements. Signal intensities found to be significantly different are shown in heat map in red (z-score above zero) and blue (z-score below zero).

Table 3 shows all sum formulas identified to be significant in a tabulated overview.

Table 3: HCA of Teheran and Hangzhou samples for negative- and positive-ion mode measurements. Signal intensities found to be significantly different are shown in red (z-score above zero) and blue (z-score below zero) with respective sum formula.

287 (-)	289 (-)	FPI17B0811 (-)	FPI17B0807 (-)	Sum formula	287 (+)	289 (+)	FPI17B0811 (+)	FPI17B0807 (+)	Sum formula
				O12 N4 Ca Na					C20 H22 O4 N
				C15 H7 O3					C10 H11 O N Na
				O18 N6 Ca2 Na					H3 O3 N K Mn
				O9 N3 Ca					C6 H12 O N
				O15 N5 Ca2					C10 H16 O3 Na
				H5 O6 Br Cu					C13 H10 N
				O9 N3 Mg					C9 H8 N
				O15 N5 Ca Na2					C8 H7 N
				O15 N5 Na4					C23 H13
				C2 H Cl Cu					C24 H12
				O2 N Cl Cu					C6 H13 N2
				C17 H2 O					C6 H9 N2
				C26 H3 O					C12 H9 O
				C19 H2 O					C8 H10 N
				H O3 S					C10 H8
				H3 O7 S Si					C6 H8 O N
				O4 Cl2 Fe S					C9 H7
				C24					C5 H6 O N
				C14 H11 O8					C10 H13 N2
				O6 N S					C9 H10 N
				H3 O6 S Si					C7 H7 N2
				H O10 N2 Mg S					C30 H14
				O4 B2 Cl4 S					C10 H11 N2
				C11 H5 O5					C7 H13 N2
				C13 H11 O N2					C11 H11 N2
				O5 N2 Ca Mn2					C14 H12 N
				C10 H6 O5 N					C5 H9 N2
				O3 Cl S					C11 H13 N2
				C9 H5 O6					C15 H14 N
				O2 Cl Cu					C11 H12 N
				O Cl Cu					C10 H10 N
				C H3 O5 S Si					C6 H11 N2
				C11 H6 O4 N					O4 K2 Na S
				H2 O9 N3 Mg S					C8 H9 N2
				C H5 O7 S Si					C8 H18 O3 K
				C2 H3 O6 S					C22 H12
				H O6 S Si					C9 H11 N2
				C10 H7 O6					C8 H15 N2
				Cd Cl3					C12 H17 N2
				O8 Fe S2					C14 H15 N2

C5 H7 O6 S
C8 H7 O4 S
H O5 S Zn
O3 N Cl2 Zn
C7 H5 O4 S
O4 Cl S Zn
C15 H O2
C7 H7 O4
C18 H29 O3 S
C2 H3 O5 S
C7 H5 O5 S
C11 H11 O6
C H2 O9 N2 Fe S
C11 H6 O3 N
H2 O4 P
C8 H7 O5 S
H2 O3 N S
C7 H11 O6 S
C9 H3 O6
C16 H2 O2
C8 H15 O6 S
H O7 N K S
C7 H O
C5 H O
C18 H2 O2
C11 H O
C14 O2
O Cl2 Cu2
C13 H O
H O7 S2
H O5 Cu S
H2 O8 K S2
C8 O2
C13 H25 O3
C14 H9 O8
O7 N Cu S
C21 H2 O
C18 H O
C23 H2
C8 H5 O5
C6 H9 O8 S
C11 H O2
C9 H O2
C7 H O2
C29 H3
C24 H3 O
C14 H2 O2

Cs
H5 O3 Fe
C16 H8
C22 H10
C20 H10
C5 H14 O N
C19 H14
H3 O2 Mn
C17 H18 O3 N
C12 H11 O N2
C10 H14 N
C7 H9 N2
C18 H22 O3 N
C18 H20 O3 N
C11 H10 N2
H3 O4 Cr
C20 H14
C13 H13 O N2
C12 H12 O3 N
C4 H9 N6
C12 H12 N2
C5 H14 O5 Al
C14 H15 O N2
C9 H16 N
C19 H20 O3 N
C7 H8 N3
C9 H9 N2
C21 H23 O5 N K
H4 O4 Mn
C8 H10 N3
C12 H14 O3 N
C4 H12 N
C7 H13 O2
C H5 O4 Fe
C6 H11 O2
C6 H14 N
C8 H11
C5 H10 O N
C21 H46 N
C13 H12 O N2
C17 H22 O4 N
C5 O Na
C21 H22 O5 N
C11 H12 O3 N
C11 H10 O3 N
C12 H9 O N2
C21 H24 O5 N

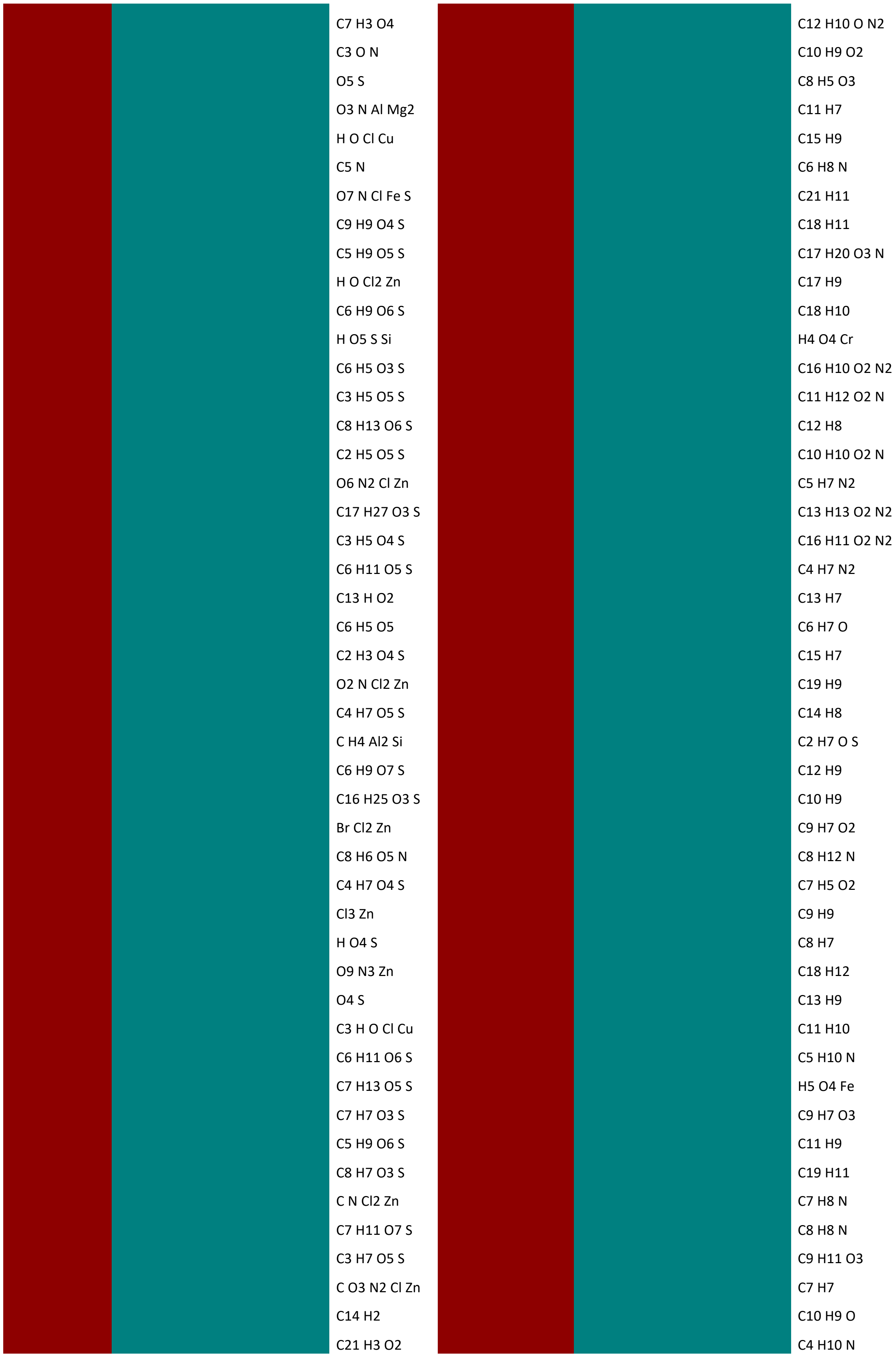
C7 H3 O4
C3 O N
O5 S
O3 N Al Mg2
H O Cl Cu
C5 N
O7 N Cl Fe S
C9 H9 O4 S
C5 H9 O5 S
H O Cl2 Zn
C6 H9 O6 S
H O5 S Si
C6 H5 O3 S
C3 H5 O5 S
C8 H13 O6 S
C2 H5 O5 S
O6 N2 Cl Zn
C17 H27 O3 S
C3 H5 O4 S
C6 H11 O5 S
C13 H O2
C6 H5 O5
C2 H3 O4 S
O2 N Cl2 Zn
C4 H7 O5 S
C H4 Al2 Si
C6 H9 O7 S
C16 H25 O3 S
Br Cl2 Zn
C8 H6 O5 N
C4 H7 O4 S
Cl3 Zn
H O4 S
O9 N3 Zn
O4 S
C3 H O Cl Cu
C6 H11 O6 S
C7 H13 O5 S
C7 H7 O3 S
C5 H9 O6 S
C8 H7 O3 S
C N Cl2 Zn
C7 H11 O7 S
C3 H7 O5 S
C O3 N2 Cl Zn
C14 H2
C21 H3 O2
C12 H10 O N2
C10 H9 O2
C8 H5 O3
C11 H7
C15 H9
C6 H8 N
C21 H11
C18 H11
C17 H20 O3 N
C17 H9
C18 H10
H4 O4 Cr
C16 H10 O2 N2
C11 H12 O2 N
C12 H8
C10 H10 O2 N
C5 H7 N2
C13 H13 O2 N2
C16 H11 O2 N2
C4 H7 N2
C13 H7
C6 H7 O
C15 H7
C19 H9
C14 H8
C2 H7 O S
C12 H9
C10 H9
C9 H7 O2
C8 H12 N
C7 H5 O2
C9 H9
C8 H7
C18 H12
C13 H9
C11 H10
C5 H10 N
H5 O4 Fe
C9 H7 O3
C11 H9
C19 H11
C7 H8 N
C8 H8 N
C9 H11 O3
C7 H7
C10 H9 O
C4 H10 N

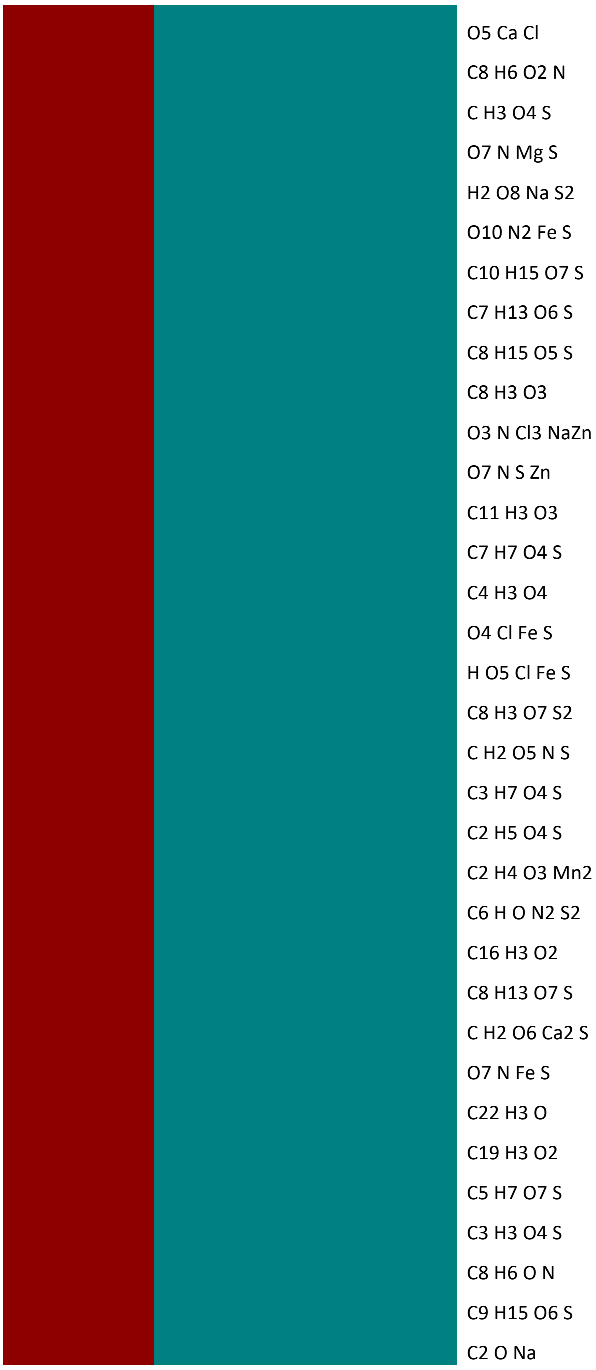
O5 Ca Cl
C8 H6 O2 N
C H3 O4 S
O7 N Mg S
H2 O8 Na S2
O10 N2 Fe S
C10 H15 O7 S
C7 H13 O6 S
C8 H15 O5 S
C8 H3 O3
O3 N Cl3 NaZn
O7 N S Zn
C11 H3 O3
C7 H7 O4 S
C4 H3 O4
O4 Cl Fe S
H O5 Cl Fe S
C8 H3 O7 S2
C H2 O5 N S
C3 H7 O4 S
C2 H5 O4 S
C2 H4 O3 Mn2
C6 H O N2 S2
C16 H3 O2
C8 H13 O7 S
C H2 O6 Ca2 S
O7 N Fe S
C22 H3 O
C19 H3 O2
C5 H7 O7 S
C3 H3 O4 S
C8 H6 O N
C9 H15 O6 S
C2 O Na

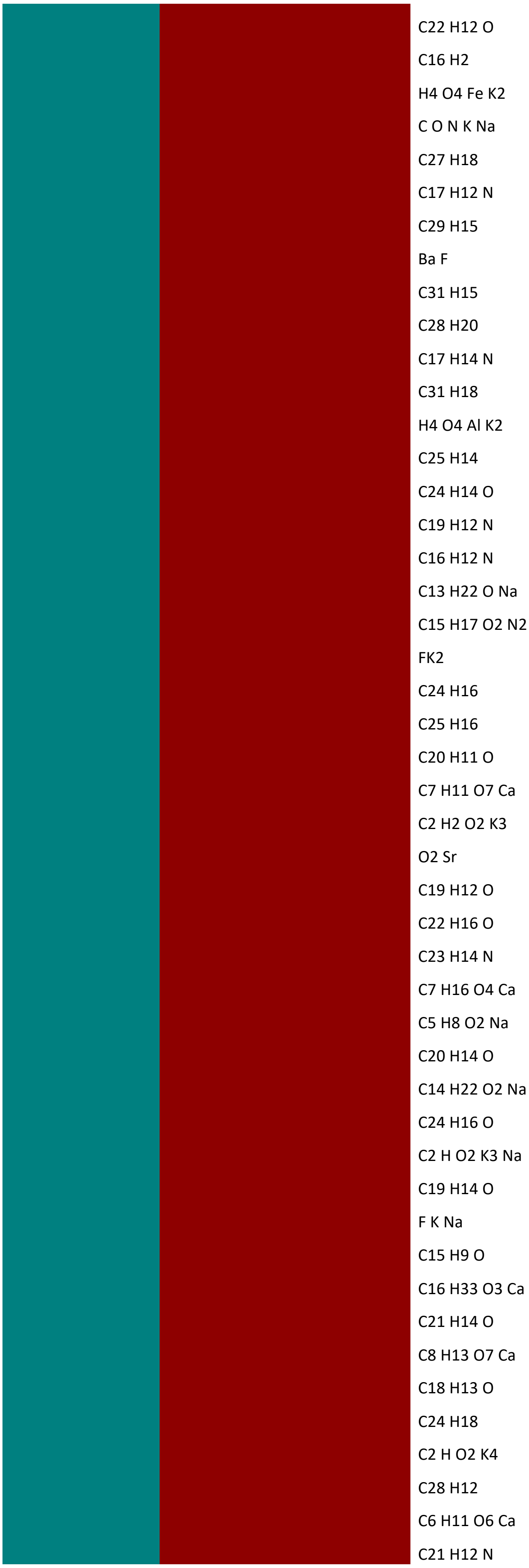
C22 H12 O
C16 H2
H4 O4 Fe K2
C O N K Na
C27 H18
C17 H12 N
C29 H15
Ba F
C31 H15
C28 H20
C17 H14 N
C31 H18
H4 O4 Al K2
C25 H14
C24 H14 O
C19 H12 N
C16 H12 N
C13 H22 O Na
C15 H17 O2 N2
FK2
C24 H16
C25 H16
C20 H11 O
C7 H11 O7 Ca
C2 H2 O2 K3
O2 Sr
C19 H12 O
C22 H16 O
C23 H14 N
C7 H16 O4 Ca
C5 H8 O2 Na
C20 H14 O
C14 H22 O2 Na
C24 H16 O
C2 H O2 K3 Na
C19 H14 O
F K Na
C15 H9 O
C16 H33 O3 Ca
C21 H14 O
C8 H13 O7 Ca
C18 H13 O
C24 H18
C2 H O2 K4
C28 H12
C6 H11 O6 Ca
C21 H12 N

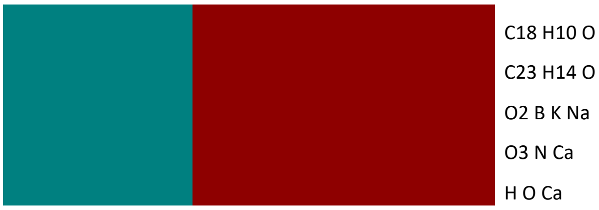
C18 H10 O
C23 H14 O
O2 B K Na
O3 N Ca
H O Ca
